# Supplementary material for: Bowel movement frequency and risks of major vascular and non-vascular diseases: a population-based cohort study among Chinese adults
Source: BMJ Open. 2020 Jan 9;10(1):e031028. doi: 10.1136/bmjopen-2019-031028 (PMC6955483; doi:10.1136/bmjopen-2019-031028)
Supplement: Supplementary data [file bmjopen-2019-031028supp001.pdf]

# Supplementary Information

## Bowel movement frequency and risks of major vascular and non-vascular diseases: a population-based cohort study among Chinese adults

Yang, S. *et al*

**Supplementary Tables** ........ **1**
Supplementary Table 1 | HRs (95% CIs) for associations between BMF and multiple vascular and non-vascular diseases with stepwise adjustment ........ 1
Supplementary Table 2 | HRs (95% CIs) for associations between BMF and multiple vascular and non-vascular diseases stratified by sex ........ 3
Supplementary Table 3 | HRs (95% CIs) for associations between BMF and multiple vascular and non-vascular diseases stratified by baseline age ........ 5
Supplementary Table 4 | HRs (95% CIs) for associations between BMF and multiple vascular and non-vascular diseases stratified by baseline hypertension ........ 7
**Supplementary Note** ........ **9**
1. CKB collaborative group ........ 9
2. Food frequency questionnaire ........ 11

# Supplementary Tables

## Supplementary Table 1 | HRs (95% CIs) for associations between BMF and multiple vascular and non-vascular diseases with stepwise adjustment

| Diseases | More than once a day | Once a day | Once every 2 to 3 days | Less than three times a week | *P* value for trend* |
|---|---|---|---|---|---|
| **Vascular diseases** | | | | | |
| **IHD** | | | | | |
| No. of participants | 46 426 | 373 054 | 46 570 | 21 148 | |
| No. of cases | 4668 | 30 470 | 3448 | 1761 | |
| Cases/PYs (1/1000) | 10.43 | 8.45 | 7.60 | 8.59 | |
| Model 1 | 1.16 (1.13-1.20) | 1.00 | 0.96 (0.93-0.99) | 1.03 (0.98-1.08) | <0.001 |
| Model 2 | 1.16 (1.13-1.20) | 1.00 | 0.96 (0.93-1.00) | 1.03 (0.98-1.08) | <0.001 |
| Model 3 | 1.17 (1.13-1.20) | 1.00 | 0.95 (0.92-0.99) | 1.01 (0.96-1.06) | <0.001 |
| Model 4 | 1.12 (1.09-1.16) | 1.00 | 1.00 (0.96-1.03) | 1.07 (1.02-1.12) | 0.008 |
| **MCEs** | | | | | |
| No. of participants | 46 426 | 373 054 | 46 570 | 21 148 | |
| No. of cases | 871 | 5960 | 765 | 376 | |
| Cases/PYs (1/1000) | 1.88 | 1.61 | 1.64 | 1.78 | |
| Model 1 | 1.05 (0.98-1.13) | 1.00 | 1.12 (1.04-1.21) | 1.28 (1.16-1.43) | <0.001 |
| Model 2 | 1.05 (0.98-1.13) | 1.00 | 1.09 (1.01-1.18) | 1.23 (1.10-1.36) | 0.005 |
| Model 3 | 1.05 (0.98-1.13) | 1.00 | 1.08 (1.00-1.16) | 1.19 (1.07-1.32) | 0.023 |
| Model 4 | 1.03 (0.96-1.11) | 1.00 | 1.10 (1.02-1.19) | 1.22 (1.10-1.36) | 0.002 |
| **Haemorrhagic stroke** | | | | | |
| No. of participants | 46 426 | 373 054 | 46 570 | 21 148 | |
| No. of cases | 1028 | 6856 | 723 | 336 | |
| Cases/PYs (1/1000) | 2.22 | 1.85 | 1.56 | 1.60 | |
| Model 1 | 1.02 (0.95-1.09) | 1.00 | 0.95 (0.88-1.03) | 1.03 (0.92-1.15) | 0.576 |
| Model 2 | 1.01 (0.94-1.08) | 1.00 | 0.93 (0.86-1.01) | 1.00 (0.89-1.11) | 0.306 |
| Model 3 | 0.99 (0.93-1.06) | 1.00 | 0.93 (0.86-1.01) | 0.98 (0.88-1.10) | 0.340 |
| Model 4 | 0.97 (0.90-1.03) | 1.00 | 0.98 (0.91-1.06) | 1.07 (0.95-1.19) | 0.256 |
| **Ischaemic stroke** | | | | | |
| No. of participants | 46 426 | 373 054 | 46 570 | 21 148 | |
| No. of cases | 4044 | 28 594 | 3291 | 1650 | |
| Cases/PYs (1/1000) | 9.00 | 7.91 | 7.24 | 8.03 | |
| Model 1 | 1.07 (1.04-1.11) | 1.00 | 0.99 (0.96-1.03) | 1.05 (1.00-1.10) | 0.178 |
| Model 2 | 1.07 (1.03-1.10) | 1.00 | 0.99 (0.95-1.03) | 1.04 (0.99-1.09) | 0.135 |
| Model 3 | 1.06 (1.03-1.10) | 1.00 | 0.99 (0.95-1.02) | 1.03 (0.98-1.08) | 0.073 |
| Model 4 | 1.01 (0.98-1.05) | 1.00 | 1.04 (1.00-1.08) | 1.10 (1.05-1.16) | 0.002 |
| **Heart failure** | | | | | |
| No. of participants | 46 426 | 373 054 | 46 570 | 21 148 | |
| No. of cases | 535 | 3068 | 398 | 203 | |

| Diseases | More than once a day | Once a day | Once every 2 to 3 days | Less than three times a week | *P* value for trend* |
|---|---|---|---|---|---|
| Cases/PYs (1/1000) | 1.16 | 0.83 | 0.86 | 0.96 | |
| Model 1 | 1.35 (1.23-1.48) | 1.00 | 0.98 (0.88-1.09) | 1.04 (0.90-1.21) | <0.001 |
| Model 2 | 1.34 (1.23-1.48) | 1.00 | 0.97 (0.87-1.08) | 1.02 (0.88-1.17) | <0.001 |
| Model 3 | 1.35 (1.23-1.48) | 1.00 | 0.95 (0.85-1.05) | 0.98 (0.85-1.13) | <0.001 |
| Model 4 | 1.33 (1.22-1.46) | 1.00 | 0.96 (0.86-1.06) | 0.99 (0.86-1.15) | <0.001 |
| **Non-vascular diseases** | | | | | |
| **COPD** | | | | | |
| No. of participants | 42 122 | 347 771 | 43 350 | 19 414 | |
| No. of cases | 1518 | 8167 | 926 | 443 | |
| Cases/PYs (1/1000) | 3.63 | 2.37 | 2.14 | 2.29 | |
| Model 1 | 1.26 (1.19-1.33) | 1.00 | 0.93 (0.87-1.00) | 1.00 (0.91-1.11) | <0.001 |
| Model 2 | 1.25 (1.19-1.33) | 1.00 | 0.92 (0.86-0.99) | 0.97 (0.88-1.07) | <0.001 |
| Model 3 | 1.26 (1.19-1.34) | 1.00 | 0.91 (0.85-0.97) | 0.94 (0.86-1.04) | <0.001 |
| Model 4 | 1.28 (1.22-1.36) | 1.00 | 0.89 (0.83-0.95) | 0.92 (0.84-1.02) | <0.001 |
| **T2DM** | | | | | |
| No. of participants | 43 703 | 353 573 | 44 037 | 19 724 | |
| No. of cases | 2207 | 11 514 | 1077 | 483 | |
| Cases/PYs (1/1000) | 5.14 | 3.30 | 2.46 | 2.46 | |
| Model 1 | 1.44 (1.38-1.51) | 1.00 | 0.73 (0.69-0.78) | 0.68 (0.62-0.75) | <0.001 |
| Model 2 | 1.42 (1.36-1.49) | 1.00 | 0.73 (0.69-0.78) | 0.68 (0.62-0.75) | <0.001 |
| Model 3 | 1.43 (1.36-1.50) | 1.00 | 0.73 (0.69-0.78) | 0.68 (0.62-0.74) | <0.001 |
| Model 4 | 1.20 (1.15-1.26) | 1.00 | 0.86 (0.81-0.92) | 0.84 (0.77-0.92) | <0.001 |
| **CKD** | | | | | |
| No. of participants | 45 706 | 368 119 | 45 846 | 20 749 | |
| No. of cases | 821 | 4786 | 605 | 314 | |
| Cases/PYs (1/1000) | 1.81 | 1.31 | 1.33 | 1.52 | |
| Model 1 | 1.20 (1.11-1.29) | 1.00 | 1.05 (0.96-1.14) | 1.20 (1.07-1.35) | 0.754 |
| Model 2 | 1.20 (1.11-1.29) | 1.00 | 1.05 (0.96-1.14) | 1.20 (1.07-1.35) | 0.755 |
| Model 3 | 1.20 (1.12-1.30) | 1.00 | 1.03 (0.95-1.12) | 1.16 (1.04-1.31) | 0.360 |
| Model 4 | 1.15 (1.07-1.24) | 1.00 | 1.07 (0.98-1.17) | 1.20 (1.07-1.35) | 0.577 |

PYs: person-years; BMF: bowel movement frequency; IHD: ischaemic heart disease; MCEs: major coronary events; COPD: chronic obstructive pulmonary disease; T2DM: type 2 diabetes mellitus; CKD: chronic kidney disease.

Model 1 was adjusted for sex.

Model 2 was additionally adjusted for level of education, occupation, household income, marital status, smoking status, and total physical activity level, and family history (for corresponding disease only; no adjustment for heart failure, COPD, and CKD).

Model 3 was additionally adjusted for alcohol consumption, and intake frequency of fresh vegetables, fresh fruits, and red meat.

Model 4 was additionally adjusted for body mass index, waist circumference, prevalent hypertension and diabetes at baseline (no adjustment for T2DM).

*Assessed by assigning consecutive integers to four BMF categories in a separate model. A Bonferroni corrected threshold was used, $\alpha$=0.00625.



Yang S, *et al. BMJ Open* 2020; 10:e031028. doi: 10.1136/bmjopen-2019-031028

**Supplementary Table 2 | HRs (95% CIs) for associations between BMF and multiple vascular and non-vascular diseases stratified by sex**

| Diseases | Subgroups | More than once a day | | Once every 1 to 3 days* | | Less than three times a week | | *P* value for interaction† |
|---|---|---|---|---|---|---|---|---|
| | | Cases | *HR (95%CI)* | Cases | *HR (95%CI)* | Cases | *HR (95%CI)* | |
| **Vascular diseases** | | | | | | | | |
| **IHD** | | | | | | | | 0.099 |
| | Men | 2335 | 1.09 (1.04-1.14) | 13 822 | 1.00 | 385 | 1.11 (1.00-1.23) | |
| | Women | 2333 | 1.16 (1.11-1.21) | 20 096 | 1.00 | 1376 | 1.05 (0.99-1.11) | |
| **MCEs** | | | | | | | | 0.756 |
| | Men | 553 | 1.00 (0.91-1.09) | 3762 | 1.00 | 137 | 1.26 (1.06-1.50) | |
| | Women | 318 | 1.05 (0.93-1.18) | 2963 | 1.00 | 239 | 1.20 (1.05-1.37) | |
| **Haemorrhagic stroke** | | | | | | | | 0.322 |
| | Men | 645 | 0.97 (0.89-1.05) | 3950 | 1.00 | 117 | 1.19 (0.99-1.43) | |
| | Women | 383 | 0.96 (0.86-1.07) | 3629 | 1.00 | 219 | 1.01 (0.88-1.16) | |
| **Ischaemic stroke** | | | | | | | | 0.006 |
| | Men | 2234 | 1.01 (0.97-1.06) | 14 229 | 1.00 | 460 | 1.25 (1.13-1.37) | |
| | Women | 1810 | 1.01 (0.96-1.06) | 17 656 | 1.00 | 1190 | 1.04 (0.98-1.11) | |
| **Heart failure** | | | | | | | | 0.559 |
| | Men | 268 | 1.26 (1.11-1.44) | 1575 | 1.00 | 53 | 0.99 (0.75-1.30) | |
| | Women | 267 | 1.42 (1.25-1.62) | 1891 | 1.00 | 150 | 0.98 (0.83-1.16) | |
| **Non-vascular diseases** | | | | | | | | |
| **COPD** | | | | | | | | 0.212 |
| | Men | 885 | 1.25 (1.16-1.35) | 4390 | 1.00 | 120 | 1.00 (0.83-1.20) | |
| | Women | 633 | 1.37 (1.26-1.49) | 4703 | 1.00 | 323 | 0.89 (0.80-1.00) | |
| **T2DM** | | | | | | | | 0.997 |
| | Men | 1035 | 1.20 (1.12-1.29) | 4721 | 1.00 | 74 | 0.89 (0.70-1.12) | |
| | Women | 1172 | 1.22 (1.15-1.30) | 7870 | 1.00 | 409 | 0.84 (0.76-0.93) | |
| **CKD** | | | | | | | | 0.249 |
| | Men | 423 | 1.13 (1.01-1.25) | 2250 | 1.00 | 75 | 1.38 (1.09-1.74) | |
| | Women | 398 | 1.16 (1.05-1.29) | 3141 | 1.00 | 239 | 1.13 (0.99-1.29) | |



Yang S, *et al. BMJ Open* 2020; 10:e031028. doi: 10.1136/bmjopen-2019-031028

BMF: bowel movement frequency; IHD: ischaemic heart disease; MCEs: major coronary events; COPD: chronic obstructive pulmonary disease; T2DM: type 2 diabetes mellitus; CKD: chronic kidney disease.

Multivariable models adjusted for same set of covariates as Table 2, except for the stratified variable of interest.

* Combined group including “once a day” and “once every 2 to 3 days”.

† A Bonferroni corrected threshold was used, $\alpha$=0.00625.



Yang S, *et al. BMJ Open* 2020; 10:e031028. doi: 10.1136/bmjopen-2019-031028

**Supplementary Table 3 | HRs (95% CIs) for associations between BMF and multiple vascular and non-vascular diseases stratified by baseline age**

| Diseases | Subgroups | More than once a day | | Once every 1 to 3 days* | | Less than three times a week | | *P* value for interaction† |
|---|---|---|---|---|---|---|---|---|
| | | Cases | *HR (95%CI)* | Cases | *HR (95%CI)* | Cases | *HR (95%CI)* | |
| **Vascular diseases** | | | | | | | | |
| **IHD** | | | | | | | | 0.362 |
| | <60 years | 2531 | 1.14 (1.09-1.19) | 18 064 | 1.00 | 810 | 1.04 (0.97-1.12) | |
| | ⩾60 years | 2137 | 1.10 (1.06-1.16) | 15 854 | 1.00 | 951 | 1.09 (1.02-1.17) | |
| **MCEs** | | | | | | | | 0.014 |
| | <60 years | 357 | 1.08 (0.96-1.21) | 2377 | 1.00 | 81 | 1.08 (0.86-1.35) | |
| | ⩾60 years | 514 | 0.97 (0.88-1.06) | 4348 | 1.00 | 295 | 1.25 (1.11-1.41) | |
| **Haemorrhagic stroke** | | | | | | | | 0.094 |
| | <60 years | 521 | 0.96 (0.87-1.05) | 3568 | 1.00 | 106 | 0.93 (0.77-1.14) | |
| | ⩾60 years | 507 | 0.97 (0.88-1.06) | 4011 | 1.00 | 230 | 1.15 (1.01-1.32) | |
| **Ischaemic stroke** | | | | | | | | 0.902 |
| | <60 years | 2212 | 1.02 (0.97-1.06) | 16 347 | 1.00 | 717 | 1.08 (1.00-1.16) | |
| | ⩾60 years | 1832 | 1.00 (0.95-1.05) | 15 538 | 1.00 | 933 | 1.11 (1.04-1.19) | |
| **Heart failure** | | | | | | | | 0.761 |
| | <60 years | 204 | 1.38 (1.19-1.61) | 1269 | 1.00 | 52 | 0.88 (0.66-1.16) | |
| | ⩾60 years | 331 | 1.31 (1.16-1.47) | 2197 | 1.00 | 151 | 1.04 (0.88-1.23) | |
| **Non-vascular diseases** | | | | | | | | |
| **COPD** | | | | | | | | 0.309 |
| | <60 years | 706 | 1.27 (1.17-1.38) | 4180 | 1.00 | 182 | 0.96 (0.82-1.11) | |
| | ⩾60 years | 812 | 1.33 (1.24-1.44) | 4913 | 1.00 | 261 | 0.91 (0.81-1.04) | |
| **T2DM** | | | | | | | | 0.331 |
| | <60 years | 1549 | 1.19 (1.13-1.26) | 8826 | 1.00 | 308 | 0.83 (0.74-0.94) | |
| | ⩾60 years | 658 | 1.25 (1.15-1.36) | 3765 | 1.00 | 175 | 0.92 (0.79-1.07) | |
| **CKD** | | | | | | | | 0.567 |
| | <60 years | 537 | 1.20 (1.10-1.32) | 3446 | 1.00 | 189 | 1.19 (1.02-1.38) | |

| Diseases | Subgroups | More than once a day | | Once every 1 to 3 days* | | Less than three times a week | | *P* value for interaction† |
|---|---|---|---|---|---|---|---|---|
| | | Cases | *HR (95%CI)* | Cases | *HR (95%CI)* | Cases | *HR (95%CI)* | |
| | ⩾60 years | 284 | 1.06 (0.94-1.20) | 1945 | 1.00 | 125 | 1.17 (0.98-1.41) | |

BMF: bowel movement frequency; IHD: ischaemic heart disease; MCEs: major coronary events; COPD: chronic obstructive pulmonary disease; T2DM: type 2 diabetes mellitus; CKD: chronic kidney disease.

Multivariable models adjusted for same set of covariates as Table 2, except for the stratified variable of interest.

* Combined group including "once a day" and "once every 2 to 3 days".

† A Bonferroni corrected threshold was used, α=0.00625.



Yang S, *et al. BMJ Open* 2020; 10:e031028. doi: 10.1136/bmjopen-2019-031028

**Supplementary Table 4 | HRs (95% CIs) for associations between BMF and multiple vascular and non-vascular diseases stratified by baseline hypertension**

| Diseases | Subgroups | More than once a day | | Once every 1 to 3 days* | | Less than three times a week | | *P* value for |
|---|---|---|---|---|---|---|---|---|
| | | Cases | *HR (95%CI)* | Cases | *HR (95%CI)* | Cases | *HR (95%CI)* | interaction† |
| **Vascular diseases** | | | | | | | | |
| **IHD** | | | | | | | | 0.192 |
| | No | 2019 | 1.17 (1.12-1.23) | 16 300 | 1.00 | 995 | 1.07 (1.00-1.14) | |
| | Yes | 2649 | 1.09 (1.04-1.13) | 17 618 | 1.00 | 766 | 1.06 (0.99-1.14) | |
| **MCEs** | | | | | | | | 0.102 |
| | No | 328 | 1.12 (1.00-1.26) | 2498 | 1.00 | 157 | 1.22 (1.04-1.44) | |
| | Yes | 543 | 0.96 (0.88-1.05) | 4227 | 1.00 | 219 | 1.20 (1.05-1.38) | |
| **Haemorrhagic stroke** | | | | | | | | 0.059 |
| | No | 266 | 1.00 (0.87-1.13) | 2108 | 1.00 | 128 | 1.17 (0.98-1.41) | |
| | Yes | 762 | 0.95 (0.88-1.03) | 5471 | 1.00 | 208 | 1.01 (0.88-1.17) | |
| **Ischaemic stroke** | | | | | | | | 0.134 |
| | No | 1441 | 1.03 (0.97-1.09) | 13 429 | 1.00 | 845 | 1.10 (1.02-1.18) | |
| | Yes | 2603 | 0.99 (0.95-1.03) | 18 456 | 1.00 | 805 | 1.09 (1.02-1.17) | |
| **Heart failure** | | | | | | | | 0.432 |
| | No | 230 | 1.47 (1.27-1.69) | 1570 | 1.00 | 102 | 0.95 (0.78-1.16) | |
| | Yes | 305 | 1.25 (1.10-1.41) | 1896 | 1.00 | 101 | 1.05 (0.85-1.28) | |
| **Non-vascular diseases** | | | | | | | | |
| **COPD** | | | | | | | | 0.654 |
| | No | 779 | 1.29 (1.20-1.40) | 5191 | 1.00 | 278 | 0.92 (0.81-1.04) | |
| | Yes | 739 | 1.30 (1.20-1.41) | 3902 | 1.00 | 165 | 0.96 (0.82-1.13) | |
| **T2DM** | | | | | | | | 0.011 |
| | No | 925 | 1.26 (1.18-1.36) | 6063 | 1.00 | 264 | 0.82 (0.72-0.93) | |
| | Yes | 1282 | 1.16 (1.10-1.24) | 6528 | 1.00 | 219 | 0.96 (0.84-1.10) | |
| **CKD** | | | | | | | | 0.255 |
| | No | 355 | 1.20 (1.08-1.35) | 2605 | 1.00 | 170 | 1.12 (0.96-1.32) | |



Yang S, *et al. BMJ Open* 2020; 10:e031028. doi: 10.1136/bmjopen-2019-031028

| **Diseases** | **Subgroups** | **More than once a day** | | **Once every 1 to 3 days*** | | **Less than three times a week** | | ***P* value for interaction†** |
|---|---|---|---|---|---|---|---|---|
| | | **Cases** | ***HR (95%CI)*** | **Cases** | ***HR (95%CI)*** | **Cases** | ***HR (95%CI)*** | |
| | Yes | 466 | 1.10 (0.99-1.21) | 2786 | 1.00 | 144 | 1.29 (1.09-1.53) | |

BMF: bowel movement frequency; IHD: ischaemic heart disease; MCEs: major coronary events; COPD: chronic obstructive pulmonary disease; T2DM: type 2 diabetes mellitus; CKD: chronic kidney disease..

Multivariable models adjusted for same set of covariates as Table 2, except for the stratified variable of interest.

* Combined group including “once a day” and “once every 2 to 3 days”.

† A Bonferroni corrected threshold was used, α=0.00625.



Yang S, *et al. BMJ Open* 2020; 10:e031028. doi: 10.1136/bmjopen-2019-031028

# Supplementary Note

## 1. CKB collaborative group

**International Steering Committee:** Junshi Chen, Zhengming Chen (PI), Robert Clarke, Rory Collins, Yu Guo, Liming Li (PI), Jun Lv, Richard Peto, Robin Walters. **International Co-ordinating Centre, Oxford:** Daniel Avery, Ruth Boxall, Derrick Bennett, Yumei Chang, Yiping Chen, Zhengming Chen, Robert Clarke, Huaidong Du, Simon Gilbert, Alex Hacker, Mike Hill, Michael Holmes, Andri Iona, Christiana Kartsonaki, Rene Kerosi, Ling Kong, Om Kurmi, Garry Lancaster, Sarah Lewington, Kuang Lin, John McDonnell, Iona Millwood, Qunhua Nie, Jayakrishnan Radhakrishnan, Paul Ryder, Sam Sansome, Dan Schmidt, Paul Sherliker, Rajani Sohoni, Becky Stevens, Iain Turnbull, Robin Walters, Jenny Wang, Lin Wang, Neil Wright, Ling Yang, Xiaoming Yang. **National Co-ordinating Centre, Beijing:** Zheng Bian, Yu Guo, Xiao Han, Can Hou, Jun Lv, Pei Pei, Chao Liu, Yunlong Tan, Canqing Yu. **10 Regional Co-ordinating Centres: Qingdao CDC:** Zengchang Pang, Ruqin Gao, Shanpeng Li, Shaojie Wang, Yongmei Liu, Ranran Du, Yajing Zang, Liang Cheng, Xiaocao Tian, Hua Zhang, Yaoming Zhai, Feng Ning, Xiaohui Sun, Feifei Li. **Licang CDC:** Silu Lv, Junzheng Wang, Wei Hou. **Heilongjiang Provincial CDC:** Mingyuan Zeng, Ge Jiang, Xue Zhou. **Nangang CDC:** Liqiu Yang, Hui He, Bo Yu, Yanjie Li, Qinai Xu,Quan Kang, Ziyan Guo. **Hainan Provincial CDC:** Dan Wang, Ximin Hu, Jinyan Chen, Yan Fu, Zhenwang Fu, Xiaohuan Wang. **Meilan CDC:** Min Weng, Zhendong Guo, Shukuan Wu,Yilei Li, Huimei Li, Zhifang Fu. **Jiangsu Provincial CDC:** Ming Wu, Yonglin Zhou, Jinyi Zhou, Ran Tao, Jie Yang, Jian Su. **Suzhou CDC:** Fang liu, Jun Zhang, Yihe Hu, Yan Lu, , Liangcai Ma, Aiyu Tang, Shuo Zhang, Jianrong Jin, Jingchao Liu. **Guangxi Provincial CDC:** Zhenzhu Tang, Naying Chen, Ying Huang. **Liuzhou CDC:** Mingqiang Li, Jinhuai Meng, Rong Pan, Qilian Jiang, Jian Lan,Yun Liu, Liuping Wei, Liyuan Zhou, Ningyu Chen Ping Wang, Fanwen Meng, Yulu Qin,, Sisi Wang. **Sichuan Provincial CDC:** Xianping Wu,



Yang S, *et al. BMJ Open* 2020; 10:e031028. doi: 10.1136/bmjopen-2019-031028

Ningmei Zhang, Xiaofang Chen,Weiwei Zhou. **Pengzhou CDC:** Guojin Luo, Jianguo Li, Xiaofang Chen, Xunfu Zhong, Jiaqiu Liu, Qiang Sun. **Gansu Provincial CDC:** Pengfei Ge, Xiaolan Ren, Caixia Dong. **Maiji CDC:** Hui Zhang, Enke Mao, Xiaoping Wang, Tao Wang, Xi zhang. **Henan Provincial CDC:** Ding Zhang, Gang Zhou, Shixian Feng, Liang Chang, Lei Fan. **Huixian CDC:** Yulian Gao, Tianyou He, Huarong Sun, Pan He, Chen Hu, Xukui Zhang, Huifang Wu, Pan He. **Zhejiang Provincial CDC:** Min Yu, Ruying Hu, Hao Wang. Tongxiang CDC: Yijian Qian, Chunmei Wang, Kaixu Xie, Lingli Chen, Yidan Zhang, Dongxia Pan, Qijun Gu. **Hunan Provincial CDC:** Yuelong Huang, Biyun Chen, Li Yin, Huilin Liu, Zhongxi Fu, Qiaohua Xu. **Liuyang CDC:** Xin Xu, Hao Zhang, Huajun Long, Xianzhi Li, Libo Zhang, Zhe Qiu.

## 2. Food frequency questionnaire

**Section 5: Diet**

**5.1 During the past 12 months, about how often did you eat the following foods?**

| | Daily | 4 to 6 days per week | 1 to 3 days per week | Monthly | Never /rarely |
|---|---|---|---|---|---|
| Rice | | | | | |
| Wheat | | | | | |
| Other staple food (corn, millet etc.) | | | | | |
| Meat | | | | | |
| Poultry | | | | | |
| Fish/sea food | | | | | |
| Fresh eggs | | | | | |
| Fresh vegetables | | | | | |
| Soybean products | | | | | |
| Preserved vegetables | | | | | |
| Fresh fruit | | | | | |
| Dairy products (milk, yogurt) | | | | | |

**5.2 During the past 12 months, have you taken the following supplements regularly?**

| Yes | No | |
|---|---|---|
| | | Fish oil/cod liver oil |
| | | Vitamins |
| | | Calcium/iron/zinc |
| | | Ginseng (at least 5 or more times during a year) |
| | | Other herbal products |

(5.3 to 5.6 are omitted)

**5.7 During the past month, about how often did you eat hot spicy food?**

- ☐ Never or almost never → Go to section 6
- ☐ Only occasionally → Go to section 6
- ☐ 1 to 2 days/week
- ☐ 3 to 5 days/week
- ☐ Daily or almost every day

The complete questionnaire is available below:
*https://www.ckbiobank.org/site/Study+Resources/Baseline+Data*

The categories of covariates in Cox models: intake frequency of rice, wheat, other staple food (daily, 4 to 6 days/week, 1 to 3 days/week, monthly or rarely or never); spicy food (6 to 7, 3 to 5, 1 to 2 days/week, occasionally, never); consumption of nutritional supplements (fish oil/cod liver oil, vitamins, calcium/iron/zinc, ginseng and other herbal products; yes or no);



Yang S, *et al. BMJ Open* 2020; 10:e031028. doi: 10.1136/bmjopen-2019-031028
