# Supplementary material for: Bowel movement frequency and risks of major vascular and non-vascular diseases: a population-based cohort study among Chinese adults
Source: BMJ Open. 2020 Jan 9;10(1):e031028. doi: 10.1136/bmjopen-2019-031028 (PMC6955483; doi:10.1136/bmjopen-2019-031028)
Supplement: Author's manuscript [file bmjopen-2019-031028.draft_revisions.pdf]

BMJ Open is committed to open peer review. As part of this commitment we make the peer review history of every article we publish publicly available.

When an article is published we post the peer reviewers' comments and the authors' responses online. We also post the versions of the paper that were used during peer review. These are the versions that the peer review comments apply to.

The versions of the paper that follow are the versions that were submitted during the peer review process. They are not the versions of record or the final published versions. They should not be cited or distributed as the published version of this manuscript.

BMJ Open is an open access journal and the full, final, typeset and author-corrected version of record of the manuscript is available on our site with no access controls, subscription charges or pay-per-view fees (http://bmjopen.bmj.com).

If you have any questions on BMJ Open's open peer review process please email info.bmjopen@bmj.com

BMJ Open

Bowel movement frequency and risks of major vascular and nonvascular diseases: population based cohort study among 0.5 million Chinese adults

Journal:	*BMJ Open*
Manuscript ID	bmjopen-2019-031028
Article Type:	Research
Date Submitted by the Author:	12-Apr-2019

Complete List of Authors:	Yang, Songchun; Peking University Health Science Center, Epidemiology & Biostatistics<br>Yu, Canqing; Peking University Health Science Center, Epidemiology & Biostatistics<br>Guo, Yu; Chinese Academy of Medical Sciences,<br>Bian, Zheng; Chinese Academy of Medical Sciences,<br>Fan, Mengyu; Peking University Health Science Center, Epidemiology & Biostatistics<br>Yang, Ling; University of Oxford, Nuffield Department of Population Health<br>Du, Huaidong; University of Oxford, Nuffield Department of Population Health<br>Chen, Yiping; University of Oxford, Nuffield Department of Population Health<br>Yan, Shichun; Heilongjiang Center for Disease Control and Prevention<br>Zang, Yajing; Qingdao Center for Disease Control and Prevention<br>Chen, Junshi; China National Center for Food Safety Risk Assessment<br>Chen, Zhengming; University of Oxford, Nuffield Department of Population Health<br>Lv, Jun; Peking University Health Science Center, Epidemiology & Biostatistics<br>LI, Li-ming; Peking University School of Public Health, Epidemiology and Biostatistics

Keywords:	EPIDEMIOLOGY, bowel movement, cohort study, chronic diseases

SCHOLARONE™
Manuscripts

Bowel movement frequency and risks of major vascular and nonvascular diseases: population based cohort study among 0.5 million Chinese adults

Songchun Yang [1], Canqing Yu [1] , Yu Guo [2], Zheng Bian [2], Mengyu Fan [1], Ling Yang [3,4], Huaidong Du [3,4], Yiping Chen [3,4], Shichun Yan [5], Yajing Zang [6], Junshi Chen [7], Zhengming Chen [4], Jun Lv [1,8,9] *, Liming Li [1] and on behalf of the China Kadoorie Biobank Collaborative Group [†]

[1] Department of Epidemiology and Biostatistics, School of Public Health, Peking University Health Science Center, Beijing 100191, China;

[2] Chinese Academy of Medical Sciences, Beijing 100730, China;

[3] Medical Research Council Population Health Research Unit, Nuffield Department of Population Health, University of Oxford, Oxford OX3 7LF, United Kingdom;

[4] Clinical Trial Service Unit and Epidemiological Studies Unit, Nuffield Department of Population Health, University of Oxford, Oxford OX3 7LF, United Kingdom;

[5] NCDs Prevention and Control Department, Heilongjiang Center for Disease Control and Prevention, Harbin 150030, Heilongjiang, China;

[6] Qingdao Center for Disease Control and Prevention, Qingdao 266033, Shandong, China;

[7] China National Center for Food Safety Risk Assessment, Beijing 100738, China;

[8] Key Laboratory of Molecular Cardiovascular Sciences (Peking University), Ministry of Education, Beijing 100191, China

[9] Peking University Institute of Environmental Medicine, Beijing 100191, China

[†] The members of steering committee and collaborative group are listed in the Appendix.

Address for correspondence

Jun Lv
Department of Epidemiology and Biostatistics
School of Public Health
Peking University Health Science Center
38 Xueyuan Road, Haidian District
Beijing 100191
China
Email: lvjun@bjmu.edu.cn
Tel: +86-10-82801528 (ext. 322)

Word count: Abstract 247, Main text 2973
(3 tables and 4 supplementary tables)

11 April 2019

For peer review only

ABSTRACT

Background: Gastrointestinal symptomatology and gut microbiota have been related to human systemic diseases. However, solid epidemiological evidence is still lacking.

Methods: Between 2004 and 2008, the China Kadoorie Biobank study recruited 0.5 million adults aged 30 to 79y from 10 diverse areas across China. A total of 487 198 participants without cancer, heart disease or stroke at baseline were included and followed up for a median 10 years. The usual bowel movement frequency was self-reported once at baseline. Information on disease incidence until end of 2016 was collected through linkages with mortality and morbidity registries and health insurance databases.

Results: After adjustment for potential confounders, participants having bowel movements "more than once a day" had higher risks of ischaemic heart disease, heart failure, chronic obstructive pulmonary disease, type 2 diabetes mellitus, and chronic kidney disease when compared with the reference group ("once a day"). The respective HRs (95% CIs) were 1.12 (1.09 to 1.16), 1.33 (1.22 to 1.46), 1.28 (1.22 to 1.36), 1.20 (1.15 to 1.26) and 1.15 (1.07, to 1.24). The lowest bowel movement frequency ("less than 3 times a week") was also associated with increased risks of ischaemic heart disease, major coronary events, ischaemic stroke, and chronic kidney disease. The respective HRs (95% CIs) were 1.07 (1.02 to 1.12), 1.22 (1.10 to 1.36), 1.11 (1.05 to 1.16), and 1.20 (1.07 to 1.35).

Conclusion: In this large epidemiological study in Chinese adults, bowel movement frequency was associated with future risks of multiple vascular and nonvascular diseases.

Strengths and limitations of this study:

Strengths:

- Prospective design, a large sample size and long-term follow-up.
- A detailed collection of lifestyle factors and other covariates.

Limitations

For peer review only - http://bmjopen.bmj.com/site/about/guidelines.xhtml

- Bowel movement frequency was self-reported once at baseline and lack of other detailed information about intestinal health.
- Residual confounding may still exist, such as total energy intake and laxative use.

What is already known on this subject?

- Gastrointestinal symptomatology and gut microbiota have been related to human systemic diseases. However, solid epidemiological evidence is still lacking.
- There is no published prospective study that has examined the association between BMF and chronic diseases outside the digestive system other than Parkinson's disease and CVDs. The findings from four prospective studies conducted in American women and Japanese that had examined the association between BMF and CVDs were inconclusive.

What this study adds?

- BMF was associated with the risk of multiple vascular and nonvascular diseases outside the digestive system independent of traditional lifestyle and intermediate risk factors for common non-communicable diseases.
- Easily assessed BMF may be helpful to identify a change from usual pattern to abnormal bowel habits, one of the health signals that deserve attention.

For peer review only

For peer review only - http://bmjopen.bmj.com/site/about/guidelines.xhtml

INTRODUCTION

Properly functioning gastrointestinal tract plays an essential role in health. Diseases of the digestion system, such as cholelithiasis, non-alcoholic liver disease, and ulcerative colitis, have been prospectively associated with cardiovascular diseases (CVDs),[1-3] type 2 diabetes mellitus (T2DM),[4] and chronic kidney disease (CKD),[5] Also, recent advancements in gut microbiota have enhanced our understanding of the potential relationship of their composition, diversity, and metabolites to diseases.[6-10]

Although gut transit time is a good indicator of gastrointestinal function, measuring it in a large epidemiology survey is quite difficult.[11] The frequency of bowel movement has been correlated with colonic transit time and may represent a simple quantifiable indicator of adequate colonic function.[12] Alternatively, bowel movement frequency (BMF) may serve as a reflection of lifestyle factors (e.g., diet and exercise)[13 14] and individual characteristics (e.g., gut microbiota).[15 16] A systematic review and meta-analysis that included four cohort studies and five case-control studies confirmed the association between constipation and subsequent diagnosis of Parkinson's disease.[17] For the association between BMF and CVDs, only four prospective studies were conducted in American women and Japanese, but findings remained inconclusive.[18-21] To our knowledge, there is no published prospective study that has examined the association between BMF and chronic diseases outside the digestive system other than Parkinson's disease and CVDs.

In the present study, we prospectively examined the associations of BMF with multiple vascular and nonvascular diseases outside the digestive system in the China Kadoorie Biobank (CKB) study of 0.5 million adults.

METHODS

Study population

CKB is an ongoing large prospective cohort study of over 0.5 million adults from 10 geographically diverse sites across China. The baseline survey was conducted during

For peer review only - http://bmjopen.bmj.com/site/about/guidelines.xhtml

2004-2008 and follow-up was initiated soon afterward. Details of the study have been described previously.[22 23] Briefly, a total of 512 715 adults aged 30-79 years had valid baseline data including the complete questionnaire, physical measurements and a written informed consent form. This study was approved by the Ethical Review Committee of the Chinese Center for Disease Control and Prevention (Beijing, China) and the Oxford Tropical Research Ethics Committee, University of Oxford (UK).

In the current study, we firstly excluded participants with previously diagnosed cancer (n=2578), heart disease (n=15 472) or stroke (n=8884) at baseline, as well as those lost to follow-up soon after baseline (n=1) and those with missing body mass index (BMI; n=2), leaving 487 198 eligible participants. Some participants with disease of interest at baseline were further excluded when conducting analyses of particular disease outcome: (i) excluding participants who had a self-reported history of emphysema, bronchitis or pulmonary heart disease (n=13 288), who was spirometry-measured to have airflow obstruction (AFO)[24] at baseline (n=23 767) for the analysis of chronic obstructive pulmonary disease (COPD); (ii) excluding participants who had a self-reported history of diabetes (n=16 162) or screen-detected diabetes at baseline (n=14 138) for the analysis of T2DM; (iii) excluding participants who had a self-reported history of kidney disease (n=7575) for the analysis of CKD.

Patient and Public Involvement

There was no patient nor public involvement in this study.

Assessment of exposure

At the baseline survey, trained staff asked study participants about BMF: “About how often do you have bowel movements each week?” The response options were: more than once a day, once a day, once every 2-3 days, or less than 3 times a week. After completion of the baseline survey in July 2008, we randomly selected about 5% of the surviving participants for resurvey in the same year. To test the reproducibility of the BMF question, we included 1300 participants who completed the same questionnaire twice at an interval of few than 1.5 years (median 1.4 years). The linearly and quadratically weighted kappa coefficients[25] between the two questions were 0.40 and

For peer review only - http://bmjopen.bmj.com/site/about/guidelines.xhtml

0.46 respectively.

Assessment of covariates

The baseline questionnaire collected covariate information: sociodemographic characteristics, lifestyle behavior, and personal and family medical history. Daily level of physical activity was calculated by multiplying the metabolic equivalent tasks (METs) value for a particular type of physical activity by hours spent on that activity per day and summing the MET-hours for all activities.[26]

Height, body weight, and waist circumference (WC) were measured with calibrated instruments. BMI was calculated as weight in kilograms divided by height in meters squared. Prevalent diabetes at baseline was defined as self-reported diabetes diagnosed by a physician before the baseline survey or screen-detected diabetes, which was defined as[27]: random blood glucose≥7.0 mmol/L and fasting time≥8h; random blood glucose≥11.1 mmol/L and fasting time<8h; or fasting blood glucose≥ 7.0 mmol/L on subsequent testing. Forced expiratory volume in 1 second (FEV1) and forced vital capacity (FVC) were measured using a hand held Micro (MS01) Spirometer (CareFusion Corp). Pre-bronchodilator FEV1/FVC<0.7 was considered as screen-detected AFO[24].

Assessment of outcomes

Information on disease incidence was identified by linking to local disease and death registries and national health insurance (HI) system, and by active follow-up. Until the end of 2016, nearly 97% of surviving participants had a successful linkage with HI data; the proportion was similar across 10 survey sites. Trained staff blinded to baseline information coded all diagnoses using the International Classification of Diseases, Tenth Revision (ICD-10).

The study outcomes of the present analysis included a range of vascular and nonvascular diseases: (i) ischaemic heart disease (IHD) (I20-I25); (ii) major coronary events (MCEs) (including non-fatal I21-I23 and fatal I21-I25); (iii) heart failure (I50); (iv) haemorrhagic stroke (I61); (v) ischaemic stroke (I63); (vi) COPD (J41-J44); (vii) T2DM (E11, E14); (viii) CKD: including diabetes mellitus with renal complications

For peer review only - http://bmjopen.bmj.com/site/about/guidelines.xhtml

(E10.2, E11.2, E12.2, E13.2, and E14.2), hypertensive renal disease (I12 and I13), glomerular disease (N03, N04, N05, and N07), renal tubulo-interstitial disease (N11, N12, N13, N14, and N15), and renal failure (N18 and N19).

Statistical analysis

We compared baseline characteristics across different BMF categories by using analysis of covariance for continuous variables and logistic regression for categorical variables, adjusting for age (years), sex and 10 survey sites as appropriate. We calculated person-years at risk from baseline until the diagnosis of the study outcome of interest, death, loss to follow-up, or 31 December 2016, whichever came first.

We used stratified Cox proportional hazards regression models to estimate the hazard ratios (HRs) and 95% confidence intervals (CIs), with age as the underlying time scale and stratified by 5-year groups and 10 survey sites. The proportional hazards assumption for the Cox model was checked by log-log plots; no apparent violation was found. The covariates adjusted in the multivariable models were determined according to previous literatures and biological relevance and included sex (for the whole cohort analysis); level of education; occupation; household income; marital status; family history of certain diseases (for corresponding disease analysis only); smoking status; total physical activity level; alcohol consumption; intake frequency of fresh vegetables, fresh fruit, and red meat; BMI; waist circumference; and prevalent hypertension and diabetes at baseline (no adjustment for T2DM). We tested the linear trend of diseases risk across various metrics of BMF by modeling the levels of ordered categorical variables as a continuous variable in a separate model.

In the sensitivity analyses, we excluded cases which occurred during the first two years to reduce the possibility of reverse causation. We also further adjusted for the following factors one by one as potential confounders: intake frequency of rice, wheat, other staple food, and spicy food; tea consumption frequency; consumption of nutritional supplements; medications for cardiovascular diseases; medical history of peptic ulcer, cirrhosis or chronic hepatitis, and gallstone or gallbladder disease at baseline.

For peer review only - http://bmjopen.bmj.com/site/about/guidelines.xhtml

To explore whether the associations between BMF and study outcomes were consistent across several baseline characteristics, we stratified the analyses by following variables: sex (men or women); age (<60 yr, ≥60 yr); obesity status defined jointly by BMI and WC (presence: BMI≥28.0 kg/m^2 or WC≥90.0cm (men)/≥85.0cm (women); absence); hypertension (presence or absence). The definition of obesity status was used because of the unique significance of abdominal obesity for the rapid growth of health risk in Asian adults. The tests for multiplicative interaction were performed using likelihood ratio tests by comparing models with and without cross-product terms between the stratifying variable and BMF categories.

P values are presented as unadjusted for multiple testing unless otherwise indicated. For testing of multiple primary outcomes, a Bonferroni-correction was applied to the significance level, α = 0.05 (two-sided), that divided 0.05 by the number of outcomes examined (5 vascular outcomes and 3 nonvascular outcomes). Analyses were conducted with Stata 15.0.

RESULTS

Baseline characteristics of study participants

Distributions of sociodemographic and lifestyle factors and health status at baseline across four categories of BMF are displayed in **Table 1**. Of the 487 198 participants analyzed, the mean age was 51.5 years; 59.1% were women, and 43.1% resided in urban areas. Participants who defecated more frequently were more likely to be men and rural residents, have higher BMI and WC on average, and more likely to be hypertensive. Among men, consuming alcohol regularly was also related to frequent bowel movements.

Associations of BMF with risks of multiple vascular and nonvascular diseases

During a median follow-up of 10.1 years (interquartile range 2.0 years), we documented 40 347 IHD, 7972 MCEs, 8943 haemorrhagic stroke, 37 579 ischaemic

For peer review only - http://bmjopen.bmj.com/site/about/guidelines.xhtml

stroke, 4204 heart failure, 11 054 COPD, 15 281 T2DM, and 6526 CKD. In the whole cohort, after multivariable adjustment, participants who reported having bowel movements "more than once a day" were associated with higher risks of IHD, heart failure, COPD, T2DM, and CKD when compared with the reference group ("once a day"). The corresponding HRs (95% CIs) were 1.12 (1.09 to 1.16), 1.33 (1.22 to 1.46), 1.28 (1.22 to 1.36), 1.20 (1.15 to 1.26) and 1.15 (1.07 to 1.24) respectively (all *P* values <0.00625; **Table 2**). At the same time, the lowest BMF ("less than 3 times a week") was associated with increased risks of IHD, MCEs, ischaemic stroke, and CKD. The corresponding HRs (95% CIs) were 1.07 (1.02 to 1.12), 1.22 (1.10 to 1.36), 1.11 (1.05 to 1.16), and 1.20 (1.07 to 1.35), respectively (all *P* values < 0.00625). We observed linear trends between BMF and risk of certain outcomes, with a positive association for heart failure, COPD, and T2DM, and a negative association for MCEs and ischaemic stroke (all *P* values for trend <0.00625) (**Table 2**). Results of stepwise adjusted models for each disease outcomes are presented in **Supplementary Table 1**. The association between BMF and T2DM was obviously attenuated after additional adjustment for BMI and waist circumference.

Sensitive analyses

In the sensitivity analyses, the associations of BMF with multiple disease outcomes did not change appreciably after excluding cases occurred in the first two years. The HRs (95% CIs) almost remained unchanged after additional adjustment for other potential confounders (data not shown).

Subgroup analyses

We found that BMF had a statistically significant multiplicative interaction with the obesity status for MCEs (*P* for interaction = 0.001). Compared to participants with BMF of once every 1-3 days, the increased risk of MCEs associated with lower BMF was only observed among obese participants (**Table 3**). There was no heterogeneity between men and women in the association between BMF and most disease outcomes except ischaemic stroke (*P* for interaction = 0.006) (**Supplementary Table 2**). The associations between BMF and all study outcomes seemed to be consistent across

For peer review only - http://bmjopen.bmj.com/site/about/guidelines.xhtml

different age groups or hypertension status. (**Supplementary Table 3** and **Supplementary Table 4**).

DISCUSSION

In this large prospective cohort study of Chinese adults, BMF was associated with the risk of multiple vascular and nonvascular diseases outside the digestive system independent of traditional lifestyle and intermediate risk factors for common non-communicable diseases. Compared with BMF of once a day, constipation was associated with an increased risk of MCEs and ischaemic stroke; more frequent bowel movements were associated with an increased risk of heart failure, COPD, and T2DM. The increase in the risk of IHD and CKD was observed in both participants who were constipated, or those had frequent bowel movements. BMF was not associated with the risk of hemorrhagic stroke. Our findings persisted after exclusion of cases occurred in the first two years.

Comparison with other studies and potential mechanism

Several prospective studies have examined the association of BMF with CVDs.[18-21] Women's Health Initiative observational study (WHI) followed 73 047 post-menopausal women for 6.4 years on average and had asked women to self-rate the severity of constipation at baseline.[18] In this study, women with severe constipation had a 23% higher risk of death from a composite of cardiovascular events (HR=1.23; 95% CI: 1.03-1.47) compared with women with no constipation. Separate analysis for each type of cardiovascular events was limited due to a small number of events. Findings from the Nurse's Health Study (NHS) of 86 289 women aged 30-55 years followed-up for nearly 30 years showed that BMF was not associated with the incidence of coronary heart disease and stroke and cardiovascular mortality.[21] Compared with women with daily bowel movements, those with BMF > 1 time/day showed a modest increase in the risk of total mortality (HR=1.10; 95% CI: 1.06-1.15).

The Ohsaki cohort, 45 112 Japanese aged 40-79 years with a follow-up of 13.3 years, has shown that the risk of overall CVD mortality increased in participants

For peer review only - http://bmjopen.bmj.com/site/about/guidelines.xhtml

reporting BMF of 1 time/2-3 days (HR=1.21; 95%: 1.08-1.35) and ≤1 time/4 days (HR=1.39; 95% CI: 1.06-1.81) compared with those reporting ≥1 time/day.[19] The respective HRs (95% CI) for death from ischaemic stroke were 1.27 (1.00-1.61) and 1.97 (1.21-3.21). The study was underpowered for analyses of IHD and other subtypes of stroke mortality and found a null association. Another similar study based on the Japan Collaborative Cohort (JACC) of 72 014 Japanese aged 40-79 years did not observe statistically significant association between BMF and death from coronary heart disease, ischaemic and haemorrhagic stroke, but was too underpowered to allow a firm conclusion.[20] However, this study indicated that laxative use, a potential reflection of severe constipation, was associated with increased risk of dying from coronary heart disease and ischaemic stroke.

In the present study, we associated constipation with increased risk of MCEs and ischaemic stroke, in line with most previous findings. With regard to the associations between BMF and other outcomes, to our knowledge, no report has yet been made in published prospective studies. Previous studies linked constipation or high frequency of bowel movements to the alterations of composition, diversity, and functions of gut microbiota.[16 28 29] Altered gut microbiota has been suggested to interact with the host through both metabolism dependent pathways and metabolism independent processes and contribute to the development and progression of diseases such as atherosclerosis, heart failure, CKD, and T2DM.[30] Similarly, the gastrointestinal tract and respiratory tract, although separate organs, are part of a shared mucosal immune system termed the gut-lung axis.[31] The microbiota and metabolites in the gut and the lungs can modulate systemic and local immunity and influence the pathogenesis of respiratory diseases.[31] Also, slow transit constipation is accompanied by reduced fermentation efficacy of complex carbohydrates, which diminishes the production of the short-chain fatty acid, an energy source for colonocytes, leading to oxidative stress.[32] Oxidative stress has also been related, with different degrees of importance, to the onset and progression of atherosclerosis, cardiovascular diseases, neurological diseases, and kidney disease.[33] Despite several biologically plausible mechanisms underlying these associations have been suggested, further research is warranted to

For peer review only - http://bmjopen.bmj.com/site/about/guidelines.xhtml

provide convincing evidence of causal connection.

Several established risk factors for chronic diseases, such as unhealthy diets, physical inactivity, and obesity may also affect BMF.[13 14] However, in the present study, the relative risk estimates for multiple disease outcomes, except T2DM, did not change appreciably after adjusting for these lifestyle factors. It indicates that the associations between BMF and multiple diseases were independent of traditional risk factors. Besides, most of the associations between BMF and multiple disease outcomes were consistent across baseline subgroups defined by sex, age, obesity status, and hypertension status in the present analyses. The significant increase in the risk of MCEs associated with constipation was only observed among obese participants. We assume that both obesity- and constipation-related oxidative stress act synergistically to increase the MCEs risk. It is still unclear what contributed to the sex difference in the association between constipation and ischaemic stroke.

Strengths and limitations

To our knowledge, this is the first prospective study examining the associations of BMF with a range of disease outcomes outside the digestive system collected through linkage to multiple data systems. The present study has a large sample size and long-term follow-up, and hence was well powered for analyses of cardiovascular subtypes and other outcomes. Participants included both men and women geographically spread across urban and rural areas of China. Prospective design and long-term follow-up minimize the potential for reverse causation. A detailed collection of lifestyle factors and other covariates allowed adjustment for several potential confounders.

Some limitations deserve attention. First, BMF was self-reported once at baseline, which may cause nondifferential misclassification and potentially attenuate true associations. Second, although we have controlled for several potential confounders, residual confounding may still exist. For example, we failed to adjust for total energy intake and laxative use. However, the prevalence of laxative use was as low as 5% in the Chinese population,[34] that may not cause a significant influence on

For peer review only - http://bmjopen.bmj.com/site/about/guidelines.xhtml

the overall results. Third, due to lack of detailed information about intestinal health, such as colonic transit time, stool consistency, and gut microbiota, a more comprehensive understanding of the relationship between gut and multiple diseases is limited.

CONCLUSION

In conclusion, we observed that BMF was associated with future risk of multiple vascular and nonvascular diseases, including CVD, COPD, T2DM, and CKD. Our findings highlight that gastrointestinal health is related to the health of multiple systems and provide preliminary epidemiological evidence for potential systemic effects of gut microbiota. Easily assessed BMF may be helpful to identify a change from usual pattern to abnormal bowel habits, one of the health signals that deserve attention. Future studies, aiming at elucidating the mechanisms underlying the associations between gastrointestinal health and a number of systemic diseases, may be strongly facilitated by our findings.

Acknowledgements: The most important acknowledgment is to the participants in the study and the members of the survey teams in each of the 10 regional centers, as well as to the project development and management teams based at Beijing, Oxford and the 10 regional centers. The members of the steering committee and collaborative group are listed in the supplemental material.

Contributors: JL and LL conceived and designed the paper. LL, ZC, and JC, as members of the CKB steering committee, designed and supervised the conduct of the whole study, obtained funding, and together with YG, ZB, LY, HD, YC, SYan, and YZ acquired the data. SYang, MF, CY, and JL analyzed the data. SYang and JL drafted the manuscript. CY, YG, ZB, MF, LY, HD, YC, SYan, YZ, JC, ZC, JL, and LL contributed to the interpretation of the results and critical revision of the manuscript for important intellectual content. All authors contributed to and approved the final manuscript. J.L. is the study guarantor.

For peer review only - http://bmjopen.bmj.com/site/about/guidelines.xhtml

Funding: This work was supported by grants (2016YFC0900500, 2016YFC0900501, 2016YFC0900504) from the National Key R&D Program of China. The CKB baseline survey and the first re-survey were supported by a grant from the Kadoorie Charitable Foundation in Hong Kong. The long-term follow-up is supported by grants from the UK Wellcome Trust (202922/Z/16/Z, 088158/Z/09/Z, 104085/Z/14/Z), National Natural Science Foundation of China (81390540, 81390541, 81390544), and Chinese Ministry of Science and Technology (2011BAI09B01). The funders had no role in the study design, data collection, data analysis and interpretation, writing of the report, or the decision to submit the article for publication.

Competing interests: None declared.

Patient consent: Obtained.

Ethics approval: The Ethical Review Committee of the Chinese Center for Disease Control and Prevention (Beijing, China) and the Oxford Tropical Research Ethics Committee, University of Oxford (UK).

Data sharing statement: See study website (www.ckbiobank.org) for access policy and procedures.

REFERENCES

1. Lee SB, Park G-M, Lee J-Y, et al. Association between non-alcoholic fatty liver disease and subclinical coronary atherosclerosis: An observational cohort study. *J Hepatol* 2018;68(5):1018-24. doi:10.1016/j.jhep.2017.12.012
2. Keller JJ, Wang J, Huang Y-L, et al. Increased risk of stroke among patients with ulcerative colitis: a population-based matched cohort study. *Int J Colorectal Dis* 2014;29(7):805-12. doi:10.1007/s00384-014-1862-6
3. Fan LL, Chen BH, Dai ZJ. The relation between gallstone disease and cardiovascular disease. *Sci Rep* 2017;7(1):15104. doi:10.1038/s41598-017-15430-5
4. Lv J, Yu C, Guo Y, et al. Gallstone Disease and the Risk of Type 2 Diabetes. *Sci Rep* 2017;7(1):15853. doi:10.1038/s41598-017-14801-2
5. Sinn DH, Kang D, Jang HR, et al. Development of chronic kidney disease in patients with non-alcoholic fatty liver disease: A cohort study. *J Hepatol* 2017;67(6):1274-80. doi:10.1016/j.jhep.2017.08.024
6. Lambeth SM, Carson T, Lowe J, et al. Composition, Diversity and Abundance of Gut Microbiome in Prediabetes and Type 2 Diabetes. *J Diabetes Obes* 2015;2(3):1-7. doi:10.15436/2376-0949.15.031
7. Yamashiro K, Tanaka R, Urabe T, et al. Gut dysbiosis is associated with metabolism and systemic inflammation in patients with ischemic stroke. *PLoS One* 2017;12(2):e0171521. doi:10.1371/journal.pone.0171521
8. Heianza Y, Ma W, Manson JE, et al. Gut Microbiota Metabolites and Risk of Major Adverse Cardiovascular

For peer review only - http://bmjopen.bmj.com/site/about/guidelines.xhtml

Disease Events and Death: A Systematic Review and Meta-Analysis of Prospective Studies. *J Am Heart Assoc* 2017;6(7):e004947. doi:10.1161/JAHA.116.004947

9. Xu KY, Xia GH, Lu JQ, et al. Impaired renal function and dysbiosis of gut microbiota contribute to increased trimethylamine-N-oxide in chronic kidney disease patients. *Sci Rep* 2017;7(1):1445. doi:10.1038/s41598-017-01387-y

10. Pasini E, Aquilani R, Testa C, et al. Pathogenic Gut Flora in Patients With Chronic Heart Failure. *JACC Heart Fail* 2016;4(3):220-7. doi:10.1016/j.jchf.2015.10.009

11. Rao SS, Camilleri M, Hasler WL, et al. Evaluation of gastrointestinal transit in clinical practice: position paper of the American and European Neurogastroenterology and Motility Societies. *Neurogastroenterol Motil* 2011;23(1):8-23. doi:10.1111/j.1365-2982.2010.01612.x

12. Tornblom H, Van Oudenhove L, Sadik R, et al. Colonic Transit Time and IBS Symptoms: What's the Link. *Am J Gastroenterol* 2012;107(5):754-60. doi:10.1038/ajg.2012.5

13. Sanjoaquin MA, Appleby PN, Spencer EA, et al. Nutrition and lifestyle in relation to bowel movement frequency: a cross-sectional study of 20,630 men and women in EPIC-Oxford. *Public Health Nutr* 2004;7(1):77-83. doi:10.1079/Phn2003522

14. Nakaji S, Tokunaga S, Sakamoto J, et al. Relationship between lifestyle factors and defecation in a Japanese population. *Eur J Nutr* 2002;41(6):244-48. doi:10.1007/s00394-002-0380-4

15. Hadizadeh F, Walter S, Belheouane M, et al. Stool frequency is associated with gut microbiota composition. *Gut* 2017;66(3):559-60. doi:10.1136/gutjnl-2016-311935

16. Oki K, Toyama M, Banno T, et al. Comprehensive analysis of the fecal microbiota of healthy Japanese adults reveals a new bacterial lineage associated with a phenotype characterized by a high frequency of bowel movements and a lean body type. *BMC Microbiol* 2016;16(1):284. doi:10.1186/s12866-016-0898-x

17. Adams-Carr KL, Bestwick JP, Shribman S, et al. Constipation preceding Parkinson's disease: a systematic review and meta-analysis. *J Neurol Neurosurg Psychiatry* 2016;87(7):710-6. doi:10.1136/jnnp-2015-311680

18. Salmoirago-Blotcher E, Crawford S, Jackson E, et al. Constipation and risk of cardiovascular disease among postmenopausal women. *Am J Med* 2011;124(8):714-23. doi:10.1016/j.amjmed.2011.03.026

19. Honkura K, Tomata Y, Sugiyama K, et al. Defecation frequency and cardiovascular disease mortality in Japan: The Ohsaki cohort study. *Atherosclerosis* 2016:246:51-56. doi:10.1016/j.atherosclerosis.2016.01.007

20. Kubota Y, Iso H, Tamakoshi A, et al. Bowel Movement Frequency, Laxative Use, and Mortality From Coronary Heart Disease and Stroke Among Japanese Men and Women: The Japan Collaborative Cohort (JACC) Study. *J Epidemiol* 2016;26(5):242-48. doi:10.2188/jea.JE20150123

21. Ma WJ, Li YP, Heianza Y, et al. Associations of Bowel Movement Frequency with Risk of Cardiovascular Disease and Mortality among US Women. *Sci Rep* 2016:6:33005. doi:10.1038/srep33005

22. Chen ZM, Lee L, Chen JS, et al. Cohort profile: The Kadoorie study of chronic disease in China (KSCDC). *Int J Epidemiol* 2005;34(6):1243-49. doi:10.1093/ije/dyi1d74

23. Chen ZM, Chen JS, Collins R, et al. China Kadoorie Biobank of 0.5 million people: survey methods, baseline characteristics and long-term follow-up. *Int J Epidemiol* 2011;40(6):1652-66. doi:10.1093/ije/dyr120

24. Kurmi OP, Li LM, Wang J, et al. COPD and its association with smoking in the Mainland China: a cross-sectional analysis of 0.5 million men and women from ten diverse areas. *Int J Chronic Obstr* 2015;10:655-65. doi:10.2147/Copd.S75454

25. Li P. A Note on the Linearly and Quadratically Weighted Kappa Coefficients. *Psychometrika* 2016;81(3):795-801. doi:10.1007/s11336-016-9501-5

26. Du HD, Li LM, Whitlock G, et al. Patterns and socio-demographic correlates of domain-specific physical activities and their associations with adiposity in the China Kadoorie Biobank study. *BMC Public Health*

For peer review only

For peer review only - http://bmjopen.bmj.com/site/about/guidelines.xhtml

2014;14:826. doi:10.1186/1471-2458-14-826

27. Bragg F, Holmes MV, Iona A, et al. Association Between Diabetes and Cause-Specific Mortality in Rural and Urban Areas of China. *J Am Med Assoc* 2017;317(3):280-89. doi:10.1001/jama.2016.19720

28. Zhuang X, Tian Z, Li L, et al. Fecal Microbiota Alterations Associated With Diarrhea-Predominant Irritable Bowel Syndrome. *Front Microbiol* 2018;9:1600. doi:10.3389/fmicb.2018.01600

29. Mancabelli L, Milani C, Lugli GA, et al. Unveiling the gut microbiota composition and functionality associated with constipation through metagenomic analyses. *Sci Rep* 2017;7(1):9879. doi:10.1038/s41598-017-10663-w

30. Tang WHW, Kitai T, Hazen SL. Gut Microbiota in Cardiovascular Health and Disease. *Circ Res* 2017;120(7):1183-96. doi:10.1161/Circresaha.117.309715

31. Budden KF, Gellatly SL, Wood DLA, et al. Emerging pathogenic links between microbiota and the gut-lung axis. *Nat Rev Microbiol* 2017;15(1):55-63. doi:10.1038/nrmicro.2016.142

32. Vermorken AJ, Andres E, Cui Y. Bowel movement frequency, oxidative stress and disease prevention. *Mol Clin Oncol* 2016;5(4):339-42. doi:10.3892/mco.2016.987

33. Pizzino G, Irrera N, Cucinotta M, et al. Oxidative Stress: Harms and Benefits for Human Health. *Oxid Med Cell Longev* 2017;2017:8416763. doi:10.1155/2017/8416763

34. Wald A, Mueller-Lissner S, Kamm MA, et al. Survey of laxative use by adults with self-defined constipation in South America and Asia: a comparison of six countries. *Aliment Pharmacol Ther* 2010;31(2):274-84. doi:10.1111/j.1365-2036.2009.04169.x

For peer review only

For peer review only - http://bmjopen.bmj.com/site/about/guidelines.xhtml

Table 1 | Baseline characteristics according to BMF at baseline for 487 198 participants

	More than once a day	Once a day	Once every 2-3 days	Less than 3 times a week
**Number of participants, n(%)**	46 426 (9.5)	373 054 (76.6)	46 570 (9.6)	21 148 (4.3)
**Women, %**	45.9	57.7	72.6	82.5
**Age, year**	53.0	51.5	50.6	51.0
**Urban area, %**	33.3	43.9	43.7	49.4
**Middle school and above, %**	46.7	49.5	49.1	48.4
**Agricultural and industrial workers, %**	58.2	57.4	57.9	57.5
**Household income ≥20 000 RMB/yr, %**	41.8	43.4	40.6	38.8
**Married, %**	91.4	91.0	90.2	89.2
**Current tobacco smoker, %**				
Men	68.2	67.5	70.4	72.5
Women	2.6	2.7	2.8	3.2
**Current daily alcohol drinker, %**				
Men	25.4	21.0	15.3	14.8
Women	1.0	1.0	0.8	0.7
**Physical activity, MET-h/d**	22.0	21.6	21.3	20.9
**Regular consumption of*, %**				
Red meat	47.0	47.4	46.9	45.3
Fresh vegetables	98.5	98.2	98.5	98.2
Fresh fruits	27.2	28.1	26.7	23.5
**Body mass index, kg/m²**	24.5	23.6	22.8	22.6
**Waist circumference, cm**	82.4	80.2	77.9	77.3
**Hypertension, %**	38.8	34.2	27.5	25.7
**Diabetes, %**	6.0	5.2	5.5	6.4
**Family history of, %**				
Cancer	18.0	16.4	16.6	17.2
Diabetes	8.3	6.7	6.7	6.8
Heart attack	3.6	3.1	3.1	3.6
Stroke	19.4	17.5	17.0	17.2

BMF, bowel movement frequency; MET, metabolic equivalent of task.

Baseline characteristics were presented as mean or percentage. Age and sex were not adjusted, and others were adjusted for age, sex, and survey sites as appropriate. Linear trend was assessed by assigning consecutive integers to four BMF categories in a separate model. All *P* values for trend were <0.001, except for agricultural and industrial workers (*P*=0.359), regular consumption of fresh vegetables (*P*=0.955), prevalence of diabetes (*P*=0.024), family history of cancer (*P*=0.010) and heart attack (*P*=0.486).

*Regular consumption was defined as consumption at least 4-6 days per week.

For peer review only - http://bmjopen.bmj.com/site/about/guidelines.xhtml

Table 2 | HRs (95% CIs) for associations between BMF and multiple vascular and nonvascular diseases

Diseases	More than once a day	Once a day	Once every 2-3 days	Less than 3 times a week	*P* value for trend*
**Vascular diseases**					
**IHD**					
No. of cases	4668	30 470	3448	1761	
Cases/PYs (1/1000)	10.43	8.45	7.60	8.59	
Multivariable adjusted	1.12 (1.09-1.16)	1.00	1.00 (0.96-1.03)	1.07 (1.02-1.12)	0.008
**MCEs**					
No. of cases	871	5960	765	376	
Cases/PYs (1/1000)	1.88	1.61	1.64	1.78	
Multivariable adjusted	1.03 (0.96-1.11)	1.00	1.10 (1.02-1.19)	1.22 (1.10-1.36)	0.002
**Haemorrhagic stroke**					
No. of cases	1028	6856	723	336	
Cases/PYs (1/1000)	2.22	1.85	1.56	1.60	
Multivariable adjusted	0.97 (0.90-1.03)	1.00	0.98 (0.91-1.06)	1.07 (0.95-1.19)	0.256
**Ischaemic stroke**					
No. of cases	4044	28 594	3291	1650	
Cases/PYs (1/1000)	9.00	7.91	7.24	8.03	
Multivariable adjusted	1.01 (0.98-1.05)	1.00	1.04 (1.00-1.08)	1.10 (1.05-1.16)	0.002
**Heart failure**					
No. of cases	535	3068	398	203	
Cases/PYs (1/1000)	1.16	0.83	0.86	0.96	
Multivariable adjusted	1.33 (1.22-1.46)	1.00	0.96 (0.86-1.06)	0.99 (0.86-1.15)	<0.001
**Nonvascular diseases**					
**COPD**					
No. of cases	1518	8167	926	443	
Cases/PYs (1/1000)	3.63	2.37	2.14	2.29	
Multivariable adjusted	1.28 (1.22-1.36)	1.00	0.89 (0.83-0.95)	0.92 (0.84-1.02)	<0.001
**T2DM**					
No. of cases	2207	11 514	1077	483	
Cases/PYs (1/1000)	5.14	3.30	2.46	2.46	
Multivariable adjusted	1.20 (1.15-1.26)	1.00	0.86 (0.81-0.92)	0.84 (0.77-0.92)	<0.001
**CKD**					
No. of cases	821	4786	605	314	
Cases/PYs (1/1000)	1.81	1.31	1.33	1.52	
Multivariable adjusted	1.15 (1.07-1.24)	1.00	1.07 (0.98-1.17)	1.20 (1.07-1.35)	0.577

HR: hazard ratio; CI: confidence interval; BMF: bowel movement frequency; IHD: ischaemic heart disease; MCEs: major coronary events; COPD: chronic obstructive pulmonary disease; T2DM: type 2 diabetes mellitus; CKD: chronic kidney disease.

Cox regression models were stratified by 5-year groups and ten survey sites, with age as the underlying time scale.

Multivariable models were adjusted for sex (men or women); level of education (no formal school, primary school, middle school,

For peer review only

high school, college, or university or higher); occupation (agricultural, industrial, administrative or managerial, professional or technical, sales or service, retired, house wife or husband, self-employed, unemployed, other); household income (<2500, 2500-4999, 5000-9999, 10 000-19 999, 20 000-34 999, ≥35 000 RMB per year); marital status (married, widowed, divorced or separated, or never married); family history of certain diseases (presence, absence, or unclear; for corresponding disease only; no adjustment for heart failure, COPD, and CKD); smoking status (nonsmoker, former smoker, current smoker who smoked <15, 15-24, or ≥25 cigarettes or equivalents per day; those who had stopped smoking because of ill health were included in current smokers to avoid bias); total physical activity level (MET-h/day); alcohol consumption (non-drinker, former weekly drinker, weekly drinker, daily drinker with an intake of <15, 15-29, 30-59, or ≥60 g/day of pure alcohol); intake frequency of fresh vegetables, fresh fruit, and red meat (daily, 4-6 days/week, 1-3 days/week, monthly, or rarely or never); BMI (kg/m2); waist circumference (cm); prevalent hypertension and diabetes at baseline (presence or absence, no adjustment for T2DM).

*Assessed by assigning consecutive integers to four BMF categories in a separate model. A Bonferroni-corrected threshold was used, α=0.00625.

For peer review only

Table 3 | HRs (95% CIs) for associations between BMF and multiple vascular and nonvascular diseases stratified by obesity status

Diseases	Obesity subgroups	More than once a day		Once every 1-3 days*		Less than 3 times a week		*P* value for interaction†
		Cases	*HR (95%CI)*	Cases	*HR (95%CI)*	Cases	*HR (95%CI)*	
**Vascular diseases**								
**IHD**								0.383
	No	2771	1.15 (1.10-1.19)	22 109	1.00	1258	1.04 (0.98-1.11)	
	Yes	1897	1.10 (1.04-1.15)	11 809	1.00	503	1.13 (1.03-1.23)	
**MCEs**								0.001
	No	577	1.07 (0.98-1.17)	4478	1.00	238	1.07 (0.93-1.22)	
	Yes	294	0.91 (0.81-1.03)	2247	1.00	138	1.57 (1.31-1.86)	
**Haemorrhagic stroke**								0.624
	No	706	0.98 (0.90-1.06)	5543	1.00	269	1.08 (0.96-1.23)	
	Yes	322	0.93 (0.82-1.04)	2036	1.00	67	1.05 (0.82-1.34)	
**Ischaemic stroke**								0.688
	No	2367	1.02 (0.98-1.07)	20 903	1.00	1221	1.10 (1.03-1.16)	
	Yes	1677	1.01 (0.96-1.06)	10 982	1.00	429	1.07 (0.97-1.18)	
**Heart failure**								0.356
	No	360	1.36 (1.22-1.52)	2493	1.00	152	0.96 (0.81-1.13)	
	Yes	175	1.25 (1.06-1.47)	973	1.00	51	1.22 (0.92-1.62)	
**Nonvascular diseases**								
**COPD**								0.353
	No	1132	1.29 (1.21-1.37)	7070	1.00	371	0.98 (0.88-1.09)	
	Yes	386	1.21 (1.08-1.35)	2023	1.00	72	0.90 (0.71-1.14)	
**T2DM**								0.506
	No	1048	1.35 (1.27-1.44)	7069	1.00	338	0.80 (0.72-0.90)	

For peer review only - http://bmjopen.bmj.com/site/about/guidelines.xhtml
For peer review only

Diseases	Obesity	More than once a day		Once every 1-3 days*		Less than 3 times a week		*P* value for
	Yes	1159	1.25 (1.17-1.33)	5522	1.00	142	0.77 (0.65-0.91)	
**CKD**								0.051
	No	545	1.22 (1.11-1.33)	3789	1.00	252	1.23 (1.08-1.40)	
	Yes	276	1.01 (0.88-1.15)	1602	1.00	62	1.05 (0.81-1.35)	

HR: hazard ratio; CI: confidence interval; BMF: bowel movement frequency; IHD: ischaemic heart disease; MCEs: major coronary events; COPD: chronic obstructive pulmonary disease; T2DM: type 2 diabetes mellitus; CKD: chronic kidney disease; BMI: body mass index; WC: waist circumference.

Multivariable models adjusted for the same set of covariates as Table 2, except for the stratified variable of interest.. Obesity status was defined according to BMI and WC, with “yes” as BMI≥ 28.0 kg/m^2 or WC≥90.0 cm (men)/≥85.0 cm (women).

*Combined group including “once a day” and “once every 2-3 days”.

†A Bonferroni-corrected threshold was used, α=0.00625.

For peer review only - http://bmjopen.bmj.com/site/about/guidelines.xhtml

Supplementary Table 1 | HRs (95% CIs) for associations between BMF and multiple vascular and nonvascular diseases with stepwise adjustment

Diseases	More than once a day	Once a day	Once every 2-3 days	Less than 3 times a week	*P* value for trend*
**Vascular diseases**					
**IHD**					
No. of participants	46 426	373 054	46 570	21 148	
No. of cases	4668	30 470	3448	1761	
Cases/PYs (1/1000)	10.43	8.45	7.60	8.59	
Model 1	1.16 (1.13-1.20)	1.00	0.96 (0.93-0.99)	1.03 (0.98-1.08)	<0.001
Model 2	1.16 (1.13-1.20)	1.00	0.96 (0.93-1.00)	1.03 (0.98-1.08)	<0.001
Model 3	1.17 (1.13-1.20)	1.00	0.95 (0.92-0.99)	1.01 (0.96-1.06)	<0.001
Model 4	1.12 (1.09-1.16)	1.00	1.00 (0.96-1.03)	1.07 (1.02-1.12)	0.008
**MCEs**					
No. of participants	46 426	373 054	46 570	21 148	
No. of cases	871	5960	765	376	
Cases/PYs (1/1000)	1.88	1.61	1.64	1.78	
Model 1	1.05 (0.98-1.13)	1.00	1.12 (1.04-1.21)	1.28 (1.16-1.43)	<0.001
Model 2	1.05 (0.98-1.13)	1.00	1.09 (1.01-1.18)	1.23 (1.10-1.36)	0.005
Model 3	1.05 (0.98-1.13)	1.00	1.08 (1.00-1.16)	1.19 (1.07-1.32)	0.023
Model 4	1.03 (0.96-1.11)	1.00	1.10 (1.02-1.19)	1.22 (1.10-1.36)	0.002
**Haemorrhagic stroke**					
No. of participants	46 426	373 054	46 570	21 148	
No. of cases	1028	6856	723	336	
Cases/PYs (1/1000)	2.22	1.85	1.56	1.60	
Model 1	1.02 (0.95-1.09)	1.00	0.95 (0.88-1.03)	1.03 (0.92-1.15)	0.576
Model 2	1.01 (0.94-1.08)	1.00	0.93 (0.86-1.01)	1.00 (0.89-1.11)	0.306
Model 3	0.99 (0.93-1.06)	1.00	0.93 (0.86-1.01)	0.98 (0.88-1.10)	0.340
Model 4	0.97 (0.90-1.03)	1.00	0.98 (0.91-1.06)	1.07 (0.95-1.19)	0.256
**Ischaemic stroke**					
No. of participants	46 426	373 054	46 570	21 148	
No. of cases	4044	28 594	3291	1650	
Cases/PYs (1/1000)	9.00	7.91	7.24	8.03	
Model 1	1.07 (1.04-1.11)	1.00	0.99 (0.96-1.03)	1.05 (1.00-1.10)	0.178
Model 2	1.07 (1.03-1.10)	1.00	0.99 (0.95-1.03)	1.04 (0.99-1.09)	0.135
Model 3	1.06 (1.03-1.10)	1.00	0.99 (0.95-1.02)	1.03 (0.98-1.08)	0.073
Model 4	1.01 (0.98-1.05)	1.00	1.04 (1.00-1.08)	1.10 (1.05-1.16)	0.002
**Heart failure**					
No. of participants	46 426	373 054	46 570	21 148	
No. of cases	535	3068	398	203	
Cases/PYs (1/1000)	1.16	0.83	0.86	0.96	
Model 1	1.35 (1.23-1.48)	1.00	0.98 (0.88-1.09)	1.04 (0.90-1.21)	<0.001

For peer review only - http://bmjopen.bmj.com/site/about/guidelines.xhtml

Diseases	More than once a day	Once a day	Once every 2-3 days	Less than 3 times a week	*P* value for trend*
Model 2	1.34 (1.23-1.48)	1.00	0.97 (0.87-1.08)	1.02 (0.88-1.17)	<0.001
Model 3	1.35 (1.23-1.48)	1.00	0.95 (0.85-1.05)	0.98 (0.85-1.13)	<0.001
Model 4	1.33 (1.22-1.46)	1.00	0.96 (0.86-1.06)	0.99 (0.86-1.15)	<0.001
**Nonvascular diseases**					
**COPD**					
No. of participants	42 122	347 771	43 350	19 414	
No. of cases	1518	8167	926	443	
Cases/PYs (1/1000)	3.63	2.37	2.14	2.29	
Model 1	1.26 (1.19-1.33)	1.00	0.93 (0.87-1.00)	1.00 (0.91-1.11)	<0.001
Model 2	1.25 (1.19-1.33)	1.00	0.92 (0.86-0.99)	0.97 (0.88-1.07)	<0.001
Model 3	1.26 (1.19-1.34)	1.00	0.91 (0.85-0.97)	0.94 (0.86-1.04)	<0.001
Model 4	1.28 (1.22-1.36)	1.00	0.89 (0.83-0.95)	0.92 (0.84-1.02)	<0.001
**T2DM**					
No. of participants	43 703	353 573	44 037	19 724	
No. of cases	2207	11 514	1077	483	
Cases/PYs (1/1000)	5.14	3.30	2.46	2.46	
Model 1	1.44 (1.38-1.51)	1.00	0.73 (0.69-0.78)	0.68 (0.62-0.75)	<0.001
Model 2	1.42 (1.36-1.49)	1.00	0.73 (0.69-0.78)	0.68 (0.62-0.75)	<0.001
Model 3	1.43 (1.36-1.50)	1.00	0.73 (0.69-0.78)	0.68 (0.62-0.74)	<0.001
Model 4	1.20 (1.15-1.26)	1.00	0.86 (0.81-0.92)	0.84 (0.77-0.92)	<0.001
**CKD**					
No. of participants	45 706	368 119	45 846	20 749	
No. of cases	821	4786	605	314	
Cases/PYs (1/1000)	1.81	1.31	1.33	1.52	
Model 1	1.20 (1.11-1.29)	1.00	1.05 (0.96-1.14)	1.20 (1.07-1.35)	0.754
Model 2	1.20 (1.11-1.29)	1.00	1.05 (0.96-1.14)	1.20 (1.07-1.35)	0.755
Model 3	1.20 (1.12-1.30)	1.00	1.03 (0.95-1.12)	1.16 (1.04-1.31)	0.360
Model 4	1.15 (1.07-1.24)	1.00	1.07 (0.98-1.17)	1.20 (1.07-1.35)	0.577

HR: hazard ratio; CI: confidence interval; BMF: bowel movement frequency; IHD: ischaemic heart disease; MCEs: major coronary events; COPD: chronic obstructive pulmonary disease; T2DM: type 2 diabetes mellitus; CKD: chronic kidney disease.

Model 1 was adjusted for sex.

Model 2 was additionally adjusted for level of education, occupation, household income, marital status, smoking status, and total physical activity level, and family history (for corresponding disease only; no adjustment for heart failure, COPD, and CKD).

Model 3 was additionally adjusted for alcohol consumption, and intake frequency of fresh vegetables, fresh fruits, and red meat.

Model 4 was additionally adjusted for body mass index, waist circumference, prevalent hypertension and diabetes at baseline (no adjustment for T2DM).

*Assessed by assigning consecutive integers to four BMF categories in a separate model. A Bonferroni-corrected threshold was used, α=0.00625.

For peer review only - http://bmjopen.bmj.com/site/about/guidelines.xhtml

Supplementary Table 2 | HRs (95% CIs) for associations between BMF and multiple vascular and nonvascular diseases stratified by sex

Diseases	Subgroups	More than once a day		Once every 1-3 days*		Less than 3 times a week		*P* value for interaction†
		Cases	*HR (95%CI)*	Cases	*HR (95%CI)*	Cases	*HR (95%CI)*	
**Vascular diseases**								
**IHD**								0.099
	Men	2335	1.09 (1.04-1.14)	13 822	1.00	385	1.11 (1.00-1.23)	
	Women	2333	1.16 (1.11-1.21)	20 096	1.00	1376	1.05 (0.99-1.11)	
**MCEs**								0.756
	Men	553	1.00 (0.91-1.09)	3762	1.00	137	1.26 (1.06-1.50)	
	Women	318	1.05 (0.93-1.18)	2963	1.00	239	1.20 (1.05-1.37)	
**Haemorrhagic stroke**								0.322
	Men	645	0.97 (0.89-1.05)	3950	1.00	117	1.19 (0.99-1.43)	
	Women	383	0.96 (0.86-1.07)	3629	1.00	219	1.01 (0.88-1.16)	
**Ischaemic stroke**								0.006
	Men	2234	1.01 (0.97-1.06)	14 229	1.00	460	1.25 (1.13-1.37)	
	Women	1810	1.01 (0.96-1.06)	17 656	1.00	1190	1.04 (0.98-1.11)	
**Heart failure**								0.559
	Men	268	1.26 (1.11-1.44)	1575	1.00	53	0.99 (0.75-1.30)	
	Women	267	1.42 (1.25-1.62)	1891	1.00	150	0.98 (0.83-1.16)	
**Nonvascular diseases**								
**COPD**								0.212
	Men	885	1.25 (1.16-1.35)	4390	1.00	120	1.00 (0.83-1.20)	
	Women	633	1.37 (1.26-1.49)	4703	1.00	323	0.89 (0.80-1.00)	
**T2DM**								0.997
	Men	1035	1.20 (1.12-1.29)	4721	1.00	74	0.89 (0.70-1.12)	
	Women	1172	1.22 (1.15-1.30)	7870	1.00	409	0.84 (0.76-0.93)	
**CKD**								0.249
	Men	423	1.13 (1.01-1.25)	2250	1.00	75	1.38 (1.09-1.74)	
	Women	398	1.16 (1.05-1.29)	3141	1.00	239	1.13 (0.99-1.29)	

For peer review only - http://bmjopen.bmj.com/site/about/guidelines.xhtml

HR: hazard ratio; CI: confidence interval; BMF: bowel movement frequency; IHD: ischaemic heart disease; MCEs: major coronary events; COPD: chronic obstructive pulmonary disease; T2DM: type 2 diabetes mellitus; CKD: chronic kidney disease.

Multivariable models adjusted for same set of covariates as Table 2, except for the stratified variable of interest.

* Combined group including "once a day" and "once every 2-3 days".

† A Bonferroni-corrected threshold was used, α=0.00625.

For peer review only

Supplementary Table 3 | HRs (95% CIs) for associations between BMF and multiple vascular and nonvascular diseases stratified by baseline age

Diseases	Subgroups	More than once a day		Once every 1-3 days*		Less than 3 times a week		*P* value for interaction†
		Cases	*HR (95%CI)*	Cases	*HR (95%CI)*	Cases	*HR (95%CI)*	
**Vascular diseases**								
**IHD**								0.362
	<60 yr	2531	1.14 (1.09-1.19)	18 064	1.00	810	1.04 (0.97-1.12)	
	⩾60 yr	2137	1.10 (1.06-1.16)	15 854	1.00	951	1.09 (1.02-1.17)	
**MCEs**								0.014
	<60 yr	357	1.08 (0.96-1.21)	2377	1.00	81	1.08 (0.86-1.35)	
	⩾60 yr	514	0.97 (0.88-1.06)	4348	1.00	295	1.25 (1.11-1.41)	
**Haemorrhagic stroke**								0.094
	<60 yr	521	0.96 (0.87-1.05)	3568	1.00	106	0.93 (0.77-1.14)	
	⩾60 yr	507	0.97 (0.88-1.06)	4011	1.00	230	1.15 (1.01-1.32)	
**Ischaemic stroke**								0.902
	<60 yr	2212	1.02 (0.97-1.06)	16 347	1.00	717	1.08 (1.00-1.16)	
	⩾60 yr	1832	1.00 (0.95-1.05)	15 538	1.00	933	1.11 (1.04-1.19)	
**Heart failure**								0.761
	<60 yr	204	1.38 (1.19-1.61)	1269	1.00	52	0.88 (0.66-1.16)	
	⩾60 yr	331	1.31 (1.16-1.47)	2197	1.00	151	1.04 (0.88-1.23)	
**Nonvascular diseases**								
**COPD**								0.309
	<60 yr	706	1.27 (1.17-1.38)	4180	1.00	182	0.96 (0.82-1.11)	
	⩾60 yr	812	1.33 (1.24-1.44)	4913	1.00	261	0.91 (0.81-1.04)	
**T2DM**								0.331
	<60 yr	1549	1.19 (1.13-1.26)	8826	1.00	308	0.83 (0.74-0.94)	
	⩾60 yr	658	1.25 (1.15-1.36)	3765	1.00	175	0.92 (0.79-1.07)	
**CKD**								0.567
	<60 yr	537	1.20 (1.10-1.32)	3446	1.00	189	1.19 (1.02-1.38)	

For peer review only - http://bmjopen.bmj.com/site/about/guidelines.xhtml

For peer review only

Diseases	Subgroups	More than once a day		Once every 1-3 days*		Less than 3 times a week		*P* value for interaction†
		Cases	*HR (95%CI)*	Cases	*HR (95%CI)*	Cases	*HR (95%CI)*	
	⩾60 yr	284	1.06 (0.94-1.20)	1945	1.00	125	1.17 (0.98-1.41)	

HR: hazard ratio; CI: confidence interval; BMF: bowel movement frequency; IHD: ischaemic heart disease; MCEs: major coronary events; COPD: chronic obstructive pulmonary disease; T2DM: type 2 diabetes mellitus; CKD: chronic kidney disease.

Multivariable models adjusted for same set of covariates as Table 2, except for the stratified variable of interest.

* Combined group including “once a day” and “once every 2-3 days”.

† A Bonferroni-corrected threshold was used, α=0.00625.

For peer review only

For peer review only - http://bmjopen.bmj.com/site/about/guidelines.xhtml

Supplementary Table 4 | HRs (95% CIs) for associations between BMF and multiple vascular and nonvascular diseases stratified by baseline hypertension

Diseases	Subgroups	More than once a day		Once every 1-3 days*		Less than 3 times a week		*P* value for interaction†
		Cases	*HR (95%CI)*	Cases	*HR (95%CI)*	Cases	*HR (95%CI)*	
**Vascular diseases**								
**IHD**								0.192
	No	2019	1.17 (1.12-1.23)	16 300	1.00	995	1.07 (1.00-1.14)	
	Yes	2649	1.09 (1.04-1.13)	17 618	1.00	766	1.06 (0.99-1.14)	
**MCEs**								0.102
	No	328	1.12 (1.00-1.26)	2498	1.00	157	1.22 (1.04-1.44)	
	Yes	543	0.96 (0.88-1.05)	4227	1.00	219	1.20 (1.05-1.38)	
**Haemorrhagic stroke**								0.059
	No	266	1.00 (0.87-1.13)	2108	1.00	128	1.17 (0.98-1.41)	
	Yes	762	0.95 (0.88-1.03)	5471	1.00	208	1.01 (0.88-1.17)	
**Ischaemic stroke**								0.134
	No	1441	1.03 (0.97-1.09)	13 429	1.00	845	1.10 (1.02-1.18)	
	Yes	2603	0.99 (0.95-1.03)	18 456	1.00	805	1.09 (1.02-1.17)	
**Heart failure**								0.432
	No	230	1.47 (1.27-1.69)	1570	1.00	102	0.95 (0.78-1.16)	
	Yes	305	1.25 (1.10-1.41)	1896	1.00	101	1.05 (0.85-1.28)	
**Nonvascular diseases**								
**COPD**								0.654
	No	779	1.29 (1.20-1.40)	5191	1.00	278	0.92 (0.81-1.04)	
	Yes	739	1.30 (1.20-1.41)	3902	1.00	165	0.96 (0.82-1.13)	
**T2DM**								0.011
	No	925	1.26 (1.18-1.36)	6063	1.00	264	0.82 (0.72-0.93)	
	Yes	1282	1.16 (1.10-1.24)	6528	1.00	219	0.96 (0.84-1.10)	
**CKD**								0.255
	No	355	1.20 (1.08-1.35)	2605	1.00	170	1.12 (0.96-1.32)	

For peer review only - http://bmjopen.bmj.com/site/about/guidelines.xhtml

Diseases	Subgroups	More than once a day		Once every 1-3 days*		Less than 3 times a week		*P* value for interaction†
		Cases	*HR (95%CI)*	Cases	*HR (95%CI)*	Cases	*HR (95%CI)*	
	Yes	466	1.10 (0.99-1.21)	2786	1.00	144	1.29 (1.09-1.53)	

HR: hazard ratio; CI: confidence interval; BMF: bowel movement frequency; IHD: ischaemic heart disease; MCEs: major coronary events; COPD: chronic obstructive pulmonary disease; T2DM: type 2 diabetes mellitus; CKD: chronic kidney disease..

Multivariable models adjusted for same set of covariates as Table 2, except for the stratified variable of interest.

* Combined group including "once a day" and "once every 2-3 days".

† A Bonferroni-corrected threshold was used, α=0.00625.

For peer review only

Supplementary file: CKB collaborative group

International Steering Committee: Junshi Chen, Zhengming Chen (PI), Robert Clarke, Rory Collins, Yu Guo, Liming Li (PI), Jun Lv, Richard Peto, Robin Walters. **International Co-ordinating Centre, Oxford:** Daniel Avery, Ruth Boxall, Derrick Bennett, Yumei Chang, Yiping Chen, Zhengming Chen, Robert Clarke, Huaidong Du, Simon Gilbert, Alex Hacker, Mike Hill, Michael Holmes, Andri Iona, Christiana Kartsonaki, Rene Kerosi, Ling Kong, Om Kurmi, Garry Lancaster, Sarah Lewington, Kuang Lin, John McDonnell, Iona Millwood, Qunhua Nie, Jayakrishnan Radhakrishnan, Paul Ryder, Sam Sansome, Dan Schmidt, Paul Sherliker, Rajani Sohoni, Becky Stevens, Iain Turnbull, Robin Walters, Jenny Wang, Lin Wang, Neil Wright, Ling Yang, Xiaoming Yang. **National Co-ordinating Centre, Beijing:** Zheng Bian, Yu Guo, Xiao Han, Can Hou, Jun Lv, Pei Pei, Chao Liu, Yunlong Tan, Canqing Yu. **10 Regional Co-ordinating Centres: Qingdao CDC:** Zengchang Pang, Ruqin Gao, Shanpeng Li, Shaojie Wang, Yongmei Liu, Ranran Du, Yajing Zang, Liang Cheng, Xiaocao Tian, Hua Zhang, Yaoming Zhai, Feng Ning, Xiaohui Sun, Feifei Li. **Licang CDC:** Silu Lv, Junzheng Wang, Wei Hou. **Heilongjiang Provincial CDC:** Mingyuan Zeng, Ge Jiang, Xue Zhou. **Nangang CDC:** Liqiu Yang, Hui He, Bo Yu, Yanjie Li, Qinai Xu,Quan Kang, Ziyan Guo. **Hainan Provincial CDC:** Dan Wang, Ximin Hu, Jinyan Chen, Yan Fu, Zhenwang Fu, Xiaohuan Wang. **Meilan CDC:** Min Weng, Zhendong Guo, Shukuan Wu,Yilei Li, Huimei Li, Zhifang Fu. **Jiangsu Provincial CDC:** Ming Wu, Yonglin Zhou, Jinyi Zhou, Ran Tao, Jie Yang, Jian Su. **Suzhou CDC:** Fang liu, Jun Zhang, Yihe Hu, Yan Lu, , Liangcai Ma, Aiyu Tang, Shuo Zhang, Jianrong Jin, Jingchao Liu. **Guangxi Provincial CDC:** Zhenzhu Tang, Naying Chen, Ying Huang. **Liuzhou CDC:** Mingqiang Li, Jinhuai Meng, Rong Pan, Qilian Jiang, Jian Lan,Yun Liu, Liuping Wei, Liyuan Zhou, Ningyu Chen Ping Wang, Fanwen Meng, Yulu Qin,, Sisi Wang. **Sichuan Provincial CDC:** Xianping Wu, Ningmei Zhang, Xiaofang Chen,Weiwei Zhou. **Pengzhou CDC:** Guojin Luo, Jianguo Li, Xiaofang Chen, Xunfu Zhong, Jiaqiu Liu, Qiang Sun. **Gansu Provincial CDC:**

For peer review only

Pengfei Ge, Xiaolan Ren, Caixia Dong. **Maiji CDC:** Hui Zhang, Enke Mao, Xiaoping Wang, Tao Wang, Xi zhang. **Henan Provincial CDC:** Ding Zhang, Gang Zhou, Shixian Feng, Liang Chang, Lei Fan. **Huixian CDC:** Yulian Gao, Tianyou He, Huarong Sun, Pan He, Chen Hu, Xukui Zhang, Huifang Wu, Pan He. **Zhejiang Provincial CDC:** Min Yu, Ruying Hu, Hao Wang. Tongxiang CDC: Yijian Qian, Chunmei Wang, Kaixu Xie, Lingli Chen, Yidan Zhang, Dongxia Pan, Qijun Gu. **Hunan Provincial CDC:** Yuelong Huang, Biyun Chen, Li Yin, Huilin Liu, Zhongxi Fu, Qiaohua Xu. **Liuyang CDC:** Xin Xu, Hao Zhang, Huajun Long, Xianzhi Li, Libo Zhang, Zhe Qiu.

For peer review only

For peer review only - http://bmjopen.bmj.com/site/about/guidelines.xhtml

STROBE Statement—Checklist of items that should be included in reports of ***cohort studies***

	Item No	Recommendation	Page No
**Title and abstract**	1	(*a*) Indicate the study's design with a commonly used term in the title or the abstract	1
		(*b*) Provide in the abstract an informative and balanced summary of what was done and what was found	2
**Introduction**			
Background/rationale	2	Explain the scientific background and rationale for the investigation being reported	4
Objectives	3	State specific objectives, including any prespecified hypotheses	4
**Methods**			
Study design	4	Present key elements of study design early in the paper	4, 5
Setting	5	Describe the setting, locations, and relevant dates, including periods of recruitment, exposure, follow-up, and data collection	5, 6
Participants	6	(*a*) Give the eligibility criteria, and the sources and methods of selection of participants. Describe methods of follow-up	5, 6
		(*b*) For matched studies, give matching criteria and number of exposed and unexposed	N/A
Variables	7	Clearly define all outcomes, exposures, predictors, potential confounders, and effect modifiers. Give diagnostic criteria, if applicable	5-8
Data sources/ measurement	8*	For each variable of interest, give sources of data and details of methods of assessment (measurement). Describe comparability of assessment methods if there is more than one group	5, 6
Bias	9	Describe any efforts to address potential sources of bias	5, 7
Study size	10	Explain how the study size was arrived at	5
Quantitative variables	11	Explain how quantitative variables were handled in the analyses. If applicable, describe which groupings were chosen and why	7
Statistical methods	12	(*a*) Describe all statistical methods, including those used to control for confounding	7, 8
		(*b*) Describe any methods used to examine subgroups and interactions	8
		(*c*) Explain how missing data were addressed	5
		(*d*) If applicable, explain how loss to follow-up was addressed	6
		(*e*) Describe any sensitivity analyses	7
**Results**			
Participants	13*	(a) Report numbers of individuals at each stage of study—eg numbers potentially eligible, examined for eligibility, confirmed eligible, included in the study, completing follow-up, and analysed	5, 8
		(b) Give reasons for non-participation at each stage	N/A
		(c) Consider use of a flow diagram	—
Descriptive data	14*	(a) Give characteristics of study participants (eg demographic, clinical, social) and information on exposures and potential confounders	8
		(b) Indicate number of participants with missing data for each variable of interest	N/A
		(c) Summarise follow-up time (eg, average and total amount)	8
Outcome data	15*	Report numbers of outcome events or summary measures over time	8

For peer review only

Main results	16	(*a*) Give unadjusted estimates and, if applicable, confounder-adjusted estimates and their precision (eg, 95% confidence interval). Make clear which confounders were adjusted for and why they were included	7, 9
		(*b*) Report category boundaries when continuous variables were categorized	8
		(*c*) If relevant, consider translating estimates of relative risk into absolute risk for a meaningful time period	—
Other analyses	17	Report other analyses done—eg analyses of subgroups and interactions, and sensitivity analyses	9
**Discussion**			
Key results	18	Summarise key results with reference to study objectives	10
Limitations	19	Discuss limitations of the study, taking into account sources of potential bias or imprecision. Discuss both direction and magnitude of any potential bias	12
Interpretation	20	Give a cautious overall interpretation of results considering objectives, limitations, multiplicity of analyses, results from similar studies, and other relevant evidence	10-12
Generalisability	21	Discuss the generalisability (external validity) of the study results	13
**Other information**			
Funding	22	Give the source of funding and the role of the funders for the present study and, if applicable, for the original study on which the present article is based	13 14

*Give information separately for exposed and unexposed groups.

Note: An Explanation and Elaboration article discusses each checklist item and gives methodological background and published examples of transparent reporting. The STROBE checklist is best used in conjunction with this article (freely available on the Web sites of PLoS Medicine at http://www.plosmedicine.org/, Annals of Internal Medicine at http://www.annals.org/, and Epidemiology at http://www.epidem.com/). Information on the STROBE Initiative is available at http://www.strobe-statement.org.

For peer review only

For peer review only - http://bmjopen.bmj.com/site/about/guidelines.xhtml

BMJ Open

Bowel movement frequency and risks of major vascular and nonvascular diseases: population based cohort study among Chinese adults

Journal:	*BMJ Open*
Manuscript ID	bmjopen-2019-031028.R1
Article Type:	Original research
Date Submitted by the Author:	03-Oct-2019
Complete List of Authors:	Yang, Songchun; Peking University Health Science Center, Epidemiology & Biostatistics<br>Yu, Canqing; Peking University Health Science Center, Epidemiology & Biostatistics<br>Guo, Yu; Chinese Academy of Medical Sciences,<br>Bian, Zheng; Chinese Academy of Medical Sciences,<br>Fan, Mengyu; Peking University Health Science Center, Epidemiology & Biostatistics<br>Yang, Ling; University of Oxford, Nuffield Department of Population Health<br>Du, Huaidong; University of Oxford, Nuffield Department of Population Health<br>Chen, Yiping; University of Oxford, Nuffield Department of Population Health<br>Yan, Shichun; Heilongjiang Center for Disease Control and Prevention<br>Zang, Yajing; Qingdao Center for Disease Control and Prevention<br>Chen, Junshi; China National Center for Food Safety Risk Assessment<br>Chen, Zhengming; University of Oxford, Nuffield Department of Population Health<br>Lv, Jun; Peking University Health Science Center, Epidemiology & Biostatistics<br>LI, Li-ming; Peking University School of Public Health, Epidemiology and Biostatistics
**Primary Subject Heading**:	Epidemiology
Secondary Subject Heading:	Public health, Cardiovascular medicine, Diabetes and endocrinology, Renal medicine, Respiratory medicine
Keywords:	EPIDEMIOLOGY, bowel movement, cohort study, chronic diseases

SCHOLARONE™
Manuscripts

Bowel movement frequency and risks of major vascular and nonvascular diseases: population based cohort study among Chinese adults

Songchun Yang [1], Canqing Yu [1] , Yu Guo [2], Zheng Bian [2], Mengyu Fan [1], Ling Yang [3,4], Huaidong Du [3,4], Yiping Chen [3,4], Shichun Yan [5], Yajing Zang [6], Junshi Chen [7], Zhengming Chen [4], Jun Lv [1,8,9] *, Liming Li [1] and on behalf of the China Kadoorie Biobank Collaborative Group †

[1] Department of Epidemiology and Biostatistics, School of Public Health, Peking University Health Science Center, Beijing 100191, China;

[2] Chinese Academy of Medical Sciences, Beijing 100730, China;

[3] Medical Research Council Population Health Research Unit, Nuffield Department of Population Health, University of Oxford, Oxford OX3 7LF, United Kingdom;

[4] Clinical Trial Service Unit and Epidemiological Studies Unit, Nuffield Department of Population Health, University of Oxford, Oxford OX3 7LF, United Kingdom;

[5] NCDs Prevention and Control Department, Heilongjiang Center for Disease Control and Prevention, Harbin 150030, Heilongjiang, China;

[6] Qingdao Center for Disease Control and Prevention, Qingdao 266033, Shandong, China;

[7] China National Center for Food Safety Risk Assessment, Beijing 100738, China;

[8] Key Laboratory of Molecular Cardiovascular Sciences (Peking University), Ministry of Education, Beijing 100191, China

[9] Peking University Institute of Environmental Medicine, Beijing 100191, China

† The members of steering committee and collaborative group are listed in the Appendix.

Address for correspondence

Jun Lv

Department of Epidemiology and Biostatistics

School of Public Health

Peking University Health Science Center

38 Xueyuan Road, Haidian District

Beijing 100191

China

Email: lvjun@bjmu.edu.cn

Tel: +86-10-82801528 (ext. 322)

Word count: Abstract 255, Main text 3230

(3 tables and 4 supplementary tables)

3 Oct 2019

For peer review only

ABSTRACT

Objective: To prospectively examine the associations of BMF with major vascular and nonvascular diseases outside the digestive system in a large population based prospective cohort study in Chinese adults.

Methods: Between 2004 and 2008, the China Kadoorie Biobank study recruited 0.5 million adults aged 30 to 79y from 10 diverse areas across China. A total of 487 198 participants without cancer, heart disease or stroke at baseline were included and followed up for a median 10 years. The usual bowel movement frequency was self-reported once at baseline. Information on disease incidence until end of 2016 was collected through linkages with mortality and morbidity registries and health insurance databases.

Results: After adjustment for potential confounders, participants having bowel movements “more than once a day” had higher risks of ischaemic heart disease, heart failure, chronic obstructive pulmonary disease, type 2 diabetes mellitus, and chronic kidney disease when compared with the reference group (“once a day”). The respective HRs (95% CIs) were 1.12 (1.09 to 1.16), 1.33 (1.22 to 1.46), 1.28 (1.22 to 1.36), 1.20 (1.15 to 1.26) and 1.15 (1.07, to 1.24). The lowest bowel movement frequency (“less than 3 times a week”) was also associated with increased risks of ischaemic heart disease, major coronary events, ischaemic stroke, and chronic kidney disease. The respective HRs (95% CIs) were 1.07 (1.02 to 1.12), 1.22 (1.10 to 1.36), 1.11 (1.05 to 1.16), and 1.20 (1.07 to 1.35).

For peer review only - http://bmjopen.bmj.com/site/about/guidelines.xhtml

Conclusions: In this large epidemiological study in Chinese adults, bowel movement frequency was associated with future risks of multiple vascular and nonvascular diseases.

For peer review only

For peer review only - http://bmjopen.bmj.com/site/about/guidelines.xhtml

INTRODUCTION

Properly functioning gastrointestinal tract plays an essential role in health. Diseases of the digestion system, such as cholelithiasis, non-alcoholic liver disease, and ulcerative colitis, have been prospectively associated with cardiovascular diseases (CVDs),[1-3] type 2 diabetes mellitus (T2DM),[4] and chronic kidney disease (CKD),[5] Also, recent advancements in gut microbiota have enhanced our understanding of the potential relationship of their composition, diversity, and metabolites to diseases.[6-10]

Although gut transit time is a good indicator of gastrointestinal function, measuring it in a large epidemiology survey is quite difficult.[11] The frequency of bowel movement has been correlated with colonic transit time and may represent a simple quantifiable indicator of adequate colonic function.[12] Our recent study has linked higher bowel movement frequency (BMF) to an increased risk of colorectal cancer (CRC) only in the subsequent five years of follow-up, indicating that abnormal increase of BMF may be an early symptom of CRC.[13]

Alternatively, BMF may serve as a reflection of lifestyle factors (e.g., diet and exercise)[14 15] and individual characteristics (e.g., gut microbiota)[16 17], which have been associated with multiple health outcomes. A systematic review and meta-analysis that included four cohort studies and five case-control studies confirmed the association between constipation and subsequent diagnosis of Parkinson's disease.[18] To our knowledge, only five cohort studies conducted in American and Japanese have examined the associations between BMF and CVDs, but findings remained

For peer review only - http://bmjopen.bmj.com/site/about/guidelines.xhtml

inconclusive.[19-23] For other outcomes, only one retrospective cohort study conducted in United States veterans has suggested that constipation is associated with higher risk of incident CKD and end-stage renal disease.[24] Solid evidences from large population-based cohort study about the associations of BMF with multiple vascular and non-vascular diseases are quite lacking.

In the present study, we prospectively examined the associations of BMF with major vascular and nonvascular diseases outside the digestive system in the China Kadoorie Biobank (CKB) study of 0.5 million adults.

SUBJECTS AND METHODS

Study population

CKB is an ongoing large prospective cohort study of over 0.5 million adults from 10 geographically diverse sites across China. The baseline survey was conducted during 2004-2008 and follow-up was initiated soon afterward. Details of the study have been described previously.[25 26] Briefly, a total of 512 715 adults aged 30-79 years had valid baseline data including the complete questionnaire, physical measurements and a written informed consent form. This study was approved by the Ethical Review Committee of the Chinese Center for Disease Control and Prevention (Beijing, China) and the Oxford Tropical Research Ethics Committee, University of Oxford (UK).

In the current study, we firstly excluded participants with previously diagnosed cancer (n=2578), heart disease (n=15 472) or stroke (n=8884) at baseline, as well as

For peer review only - http://bmjopen.bmj.com/site/about/guidelines.xhtml

those lost to follow-up soon after baseline (n=1) and those with missing body mass index (BMI; n=2), leaving 487 198 eligible participants. Some participants with disease of interest at baseline were further excluded when conducting analyses of particular disease outcome: (i) excluding participants who had a self-reported history of emphysema, bronchitis or pulmonary heart disease (n=13 288), who was spirometry-measured to have airflow obstruction (AFO)[27] at baseline (n=23 767) for the analysis of chronic obstructive pulmonary disease (COPD); (ii) excluding participants who had a self-reported history of diabetes (n=16 162) or screen-detected diabetes at baseline (n=14 138) for the analysis of T2DM; (iii) excluding participants who had a self-reported history of kidney disease (n=7575) for the analysis of CKD.

Assessment of exposure

At the baseline survey, trained staff asked study participants about BMF: “About how often do you have bowel movements each week?” The response options were: more than once a day, once a day, once every 2-3 days, or less than 3 times a week. After completion of the baseline survey in July 2008, we randomly selected about 5% of the surviving participants for resurvey in the same year. To test the reproducibility of the BMF question, we included 1300 participants who completed the same questionnaire twice at an interval of few than 1.5 years (median 1.4 years). The linearly and quadratically weighted kappa coefficients[28] between the two questions were 0.40 and 0.46 respectively.

For peer review only

For peer review only - http://bmjopen.bmj.com/site/about/guidelines.xhtml

Assessment of covariates

The baseline questionnaire collected covariate information: sociodemographic characteristics, lifestyle behavior, dietary habits, and personal and family medical history. Daily level of physical activity was calculated by multiplying the metabolic equivalent tasks (METs) value for a particular type of physical activity by hours spent on that activity per day and summing the MET-hours for all activities.[29] Dietary habits in the past 12 months were assessed using a short validated qualitative food frequency questionnaire (Supplementary File).[30]

Height, body weight, and waist circumference (WC) were measured with calibrated instruments. BMI was calculated as weight in kilograms divided by height in meters squared. Prevalent diabetes at baseline was defined as self-reported diabetes diagnosed by a physician before the baseline survey or screen-detected diabetes, which was defined as[31]: random blood glucose≥7.0 mmol/L and fasting time≥8h; random blood glucose≥11.1 mmol/L and fasting time<8h; or fasting blood glucose≥ 7.0 mmol/L on subsequent testing. Forced expiratory volume in 1 second (FEV1) and forced vital capacity (FVC) were measured using a hand held Micro (MS01) Spirometer (CareFusion Corp). Pre-bronchodilator FEV1/FVC<0.7 was considered as screen-detected AFO[27].

Assessment of outcomes

Information on disease incidence was identified by linking to local disease and death registries and national health insurance (HI) system, and by active follow-up. Until

For peer review only - http://bmjopen.bmj.com/site/about/guidelines.xhtml

the end of 2016, nearly 97% of surviving participants had a successful linkage with HI data; the proportion was similar across 10 survey sites. Trained staff blinded to baseline information coded all diagnoses using the International Classification of Diseases, Tenth Revision (ICD-10).

The study outcomes of the present analysis included a range of vascular and nonvascular diseases: (i) ischaemic heart disease (IHD) (I20-I25); (ii) major coronary events (MCEs) (including non-fatal I21-I23 and fatal I21-I25); (iii) heart failure (I50); (iv) haemorrhagic stroke (I61); (v) ischaemic stroke (I63); (vi) COPD (J41-J44); (vii) T2DM (E11, E14); (viii) CKD: including diabetes mellitus with renal complications (E10.2, E11.2, E12.2, E13.2, and E14.2), hypertensive renal disease (I12 and I13), glomerular disease (N03, N04, N05, and N07), renal tubulo-interstitial disease (N11, N12, N13, N14, and N15), and renal failure (N18 and N19).

Statistical analysis

We compared baseline characteristics across different BMF categories by using analysis of covariance for continuous variables and logistic regression for categorical variables, adjusting for age (years), sex and 10 survey sites as appropriate. We calculated person-years at risk from baseline until the diagnosis of the study outcome of interest, death, loss to follow-up, or 31 December 2016, whichever came first.

We used stratified Cox proportional hazards regression models to estimate the hazard ratios (HRs) and 95% confidence intervals (CIs), with age as the underlying time scale and stratified by 5-year groups and 10 survey sites. The proportional

For peer review only

For peer review only - http://bmjopen.bmj.com/site/about/guidelines.xhtml

hazards assumption for the Cox model was checked by log-log plots; no apparent violation was found. The covariates adjusted in the multivariable models were determined according to previous literatures and biological relevance and included sex (for the whole cohort analysis); level of education; occupation; household income; marital status; family history of certain diseases (for corresponding disease analysis only); smoking status; total physical activity level; alcohol consumption; intake frequency of fresh vegetables, fresh fruit, and red meat; BMI; waist circumference; and prevalent hypertension and diabetes at baseline (no adjustment for T2DM). We tested the linear trend of diseases risk across various metrics of BMF by modeling the levels of ordered categorical variables as a continuous variable in a separate model.

In the sensitivity analyses, we excluded cases which occurred during the first two years to reduce the possibility of reverse causation. We also further adjusted for the following factors one by one as potential confounders: intake frequency of rice, wheat, other staple food and spicy food; tea consumption frequency; consumption of nutritional supplements (fish oil/cod liver oil, vitamins, calcium/iron/zinc, ginseng and other herbal products); medications for cardiovascular diseases (aspirin, ACE-I, beta-blocker, statins, diuretics and Ca^{2+} antagonist); medical history of peptic ulcer, cirrhosis or chronic hepatitis, and gallstone or gallbladder disease at baseline.

To explore whether the associations between BMF and study outcomes were consistent across several baseline characteristics, we stratified the analyses by following variables: sex (men or women); age (<60 yr, ≥60 yr); obesity status defined

For peer review only - http://bmjopen.bmj.com/site/about/guidelines.xhtml

jointly by BMI and WC (presence: BMI≥28.0 kg/m^2 or WC≥90.0cm (men)/≥85.0cm (women)[32]; absence); hypertension (presence or absence). The definition of obesity status was used because of the unique significance of abdominal obesity for the rapid growth of health risk in Asian adults. The tests for multiplicative interaction were performed using likelihood ratio tests by comparing models with and without cross-product terms between the stratifying variable and BMF categories.

P values are presented as unadjusted for multiple testing unless otherwise indicated. For testing of multiple primary outcomes, a Bonferroni-correction was applied to the significance level, $\alpha = 0.05$ (two-sided), that divided 0.05 by the number of outcomes examined (5 vascular outcomes and 3 nonvascular outcomes). Analyses were conducted with Stata 15.0.

Patient and Public Involvement

Patients were not involved in the present study. The results of the main study were presented to study participants at the website of the CKB study (http://www.ckbiobank.org/site/) and by newsletters annually.

RESULTS

Baseline characteristics of study participants

Distributions of sociodemographic and lifestyle factors and health status at baseline across four categories of BMF are displayed in **Table 1**. Of the 487 198 participants analyzed, the mean age was 51.5 years; 59.1% were women, and 43.1% resided in

For peer review only - http://bmjopen.bmj.com/site/about/guidelines.xhtml

urban areas. Participants who defecated more frequently were more likely to be men and rural residents, have higher BMI and WC on average, and more likely to be hypertensive. Among men, consuming alcohol regularly was also related to frequent bowel movements.

Associations of BMF with risks of multiple vascular and nonvascular diseases

During a median follow-up of 10.1 years (interquartile range 2.0 years), we documented 40 347 IHD, 7972 MCEs, 8943 haemorrhagic stroke, 37 579 ischaemic stroke, 4204 heart failure, 11 054 COPD, 15 281 T2DM, and 6526 CKD. In the whole cohort, after multivariable adjustment, participants who reported having bowel movements "more than once a day" were associated with higher risks of IHD, heart failure, COPD, T2DM, and CKD when compared with the reference group ("once a day"). The corresponding HRs (95% CIs) were 1.12 (1.09 to 1.16), 1.33 (1.22 to 1.46), 1.28 (1.22 to 1.36), 1.20 (1.15 to 1.26) and 1.15 (1.07 to 1.24) respectively (all *P* values <0.00625; **Table 2**). At the same time, the lowest BMF ("less than 3 times a week") was associated with increased risks of IHD, MCEs, ischaemic stroke, and CKD. The corresponding HRs (95% CIs) were 1.07 (1.02 to 1.12), 1.22 (1.10 to 1.36), 1.11 (1.05 to 1.16), and 1.20 (1.07 to 1.35), respectively (all *P* values < 0.00625). We observed linear trends between BMF and risk of certain outcomes, with a positive association for heart failure, COPD, and T2DM, and a negative association for MCEs and ischaemic stroke (all *P* values for trend <0.00625) (**Table 2**). Results

For peer review only - http://bmjopen.bmj.com/site/about/guidelines.xhtml

of stepwise adjusted models for each disease outcomes are presented in **Supplementary Table 1**. The association between BMF and T2DM was obviously attenuated after additional adjustment for BMI and waist circumference.

Sensitive analyses

In the sensitivity analyses, the associations of BMF with multiple disease outcomes did not change appreciably after excluding cases occurred in the first two years. The HRs (95% CIs) almost remained unchanged after additional adjustment for other potential confounders (data not shown).

Subgroup analyses

We found that BMF had a statistically significant multiplicative interaction with the obesity status for MCEs (*P* for interaction = 0.001). Compared to participants with BMF of once every 1-3 days, the increased risk of MCEs associated with lower BMF was only observed among obese participants (**Table 3**). There was no heterogeneity between men and women in the association between BMF and most disease outcomes except ischaemic stroke (*P* for interaction = 0.006) (**Supplementary Table 2**). The associations between BMF and all study outcomes seemed to be consistent across different age groups or hypertension status. (**Supplementary Table 3** and **Supplementary Table 4**).

DISCUSSION

In this large prospective cohort study of Chinese adults, BMF was associated with the

For peer review only - http://bmjopen.bmj.com/site/about/guidelines.xhtml

risk of multiple vascular and nonvascular diseases outside the digestive system independent of traditional lifestyle and intermediate risk factors for common non-communicable diseases. Compared with BMF of once a day, constipation was associated with an increased risk of MCEs and ischaemic stroke; more frequent bowel movements were associated with an increased risk of heart failure, COPD, and T2DM. The increase in the risk of IHD and CKD was observed in both participants who were constipated, or those had frequent bowel movements. BMF was not associated with the risk of hemorrhagic stroke. Our findings persisted after exclusion of cases occurred in the first two years.

Comparison with other studies and potential mechanism

Several prospective studies have examined the association of BMF with CVDs.[19-22] Women's Health Initiative observational study (WHI) followed 73 047 post-menopausal women for 6.4 years on average and had asked women to self-rate the severity of constipation at baseline.[19] In this study, women with severe constipation had a 23% higher risk of death from a composite of cardiovascular events (HR=1.23; 95% CI: 1.03-1.47) compared with women with no constipation. Separate analysis for each type of cardiovascular events was limited due to a small number of events. Another retrospective cohort study conducted in over 3 million American veterans, with 93.2% of men, showed similar results.[23] In this study, constipation was defined as either having ≥2 prescriptions for ≥ 30-day supply of laxatives each or having at least two diagnoses for constipation identified by ICD-9-CM. Compared with

For peer review only - http://bmjopen.bmj.com/site/about/guidelines.xhtml

veterans without constipation, those with constipation had higher incidence of coronary heart disease (HR=1.11; 95% CI: 1.08-1.14) and ischaemic stroke (HR=1.19; 95% CI:1.15-1.22). However, findings from the Nurse's Health Study (NHS) of 86 289 women aged 30-55 years followed-up for nearly 30 years showed that BMF was not associated with the incidence of coronary heart disease and stroke and cardiovascular mortality.[22] Compared with women with daily bowel movements, those with BMF > 1 time/day showed a modest increase in the risk of total mortality (HR=1.10; 95% CI: 1.06-1.15).

The Ohsaki cohort, 45 112 Japanese aged 40-79 years with a follow-up of 13.3 years, has shown that the risk of overall CVD mortality increased in participants reporting BMF of 1 time/2-3 days (HR=1.21; 95%: 1.08-1.35) and ≤1 time/4 days (HR=1.39; 95% CI: 1.06-1.81) compared with those reporting ≥1 time/day.[20] The respective HRs (95% CI) for death from ischaemic stroke were 1.27 (1.00-1.61) and 1.97 (1.21-3.21). The study was underpowered for analyses of IHD and other subtypes of stroke mortality and found a null association. Another similar study based on the Japan Collaborative Cohort (JACC) of 72 014 Japanese aged 40-79 years did not observe statistically significant association between BMF and death from coronary heart disease, ischaemic and haemorrhagic stroke, but was too underpowered to allow a firm conclusion.[21] However, this study indicated that laxative use, a potential reflection of severe constipation, was associated with increased risk of dying from coronary heart disease and ischaemic stroke.

For peer review only

For peer review only - http://bmjopen.bmj.com/site/about/guidelines.xhtml

In the present study, we associated constipation with increased risk of MCEs and ischaemic stroke, in line with most previous findings. With regard to the associations between BMF and other outcomes, to our knowledge, only one retrospective cohort study conducted in American veterans has examined the association between constipation and incident CKD.[24] In this study, veterans with constipation had higher risks of CKD (HR=1.13; 95% CI: 1.11-1.14) and end-stage renal disease (HR=1.09; 95% CI: 1.01-1.18) compared with those without constipation.

Previous studies linked constipation or high frequency of bowel movements to the alterations of composition, diversity, and functions of gut microbiota.[17 33 34] Altered gut microbiota has been suggested to interact with the host through both metabolism dependent pathways and metabolism independent processes and contribute to the development and progression of diseases such as atherosclerosis, heart failure, CKD, and T2DM.[35] Similarly, the gastrointestinal tract and respiratory tract, although separate organs, are part of a shared mucosal immune system termed the gut-lung axis.[36] The microbiota and metabolites in the gut and the lungs can modulate systemic and local immunity and influence the pathogenesis of respiratory diseases.[36] Also, slow transit constipation is accompanied by reduced fermentation efficacy of complex carbohydrates, which diminishes the production of the short-chain fatty acid, an energy source for colonocytes, leading to oxidative stress.[37] Oxidative stress has also been related, with different degrees of importance, to the onset and progression of atherosclerosis, cardiovascular diseases, neurological

For peer review only - http://bmjopen.bmj.com/site/about/guidelines.xhtml

diseases, and kidney disease.[38] Despite several biologically plausible mechanisms underlying these associations have been suggested, further research is warranted to provide convincing evidence of causal connection.

Dietary habits are one of the factors that may influence BMF. Also, previous studies in the CKB have shown that consumption of fresh fruit, fresh egg, and spicy foods was associated with lower risks of cardiovascular diseases[39-41] or diabetes[42]. In the present study, the relative risk estimates for multiple disease outcomes did not change appreciably after adjusting for these dietary factors, indicating that the associations between BMF and multiple diseases might be independent of dietary factors. Other established risk factors for chronic diseases that may affect BMF, such as physical inactivity and obesity[14 15], did not confound these associations either, except for obesity for T2DM. Besides, most of the associations between BMF and multiple disease outcomes were consistent across baseline subgroups defined by sex, age, obesity status, and hypertension status in the present analyses. The significant increase in the risk of MCEs associated with constipation was only observed among obese participants. We assume that both obesity- and constipation-related oxidative stress act synergistically to increase the MCEs risk. It is still unclear what contributed to the sex difference in the association between constipation and ischaemic stroke.

Strengths and limitations of this study

To our knowledge, this is the first prospective study examining the associations of BMF with a range of disease outcomes outside the digestive system collected through

For peer review only - http://bmjopen.bmj.com/site/about/guidelines.xhtml

linkage to multiple data systems. The present study has a large sample size and long-term follow-up, and hence was well powered for analyses of cardiovascular subtypes and other outcomes. Participants included both men and women geographically spread across urban and rural areas of China. Prospective design and long-term follow-up minimize the potential for reverse causation. A detailed collection of lifestyle factors and other covariates allowed adjustment for several potential confounders.

Some limitations deserve attention. First, BMF was self-reported, which may cause nondifferential misclassification and potentially attenuate true associations. BMF and covariate information collected once at baseline might have changed during the follow-up. Second, although we have controlled for several potential confounders, residual confounding may still exist. For example, we failed to adjust for total energy intake, laxative use, opioids use, and other non-cardiovascular medications that might directly affect BMF. Third, due to lack of detailed information about intestinal health, such as colonic transit time, stool consistency, gut microbiota, and blood biomarkers, a more comprehensive understanding of the relationship between gut and multiple diseases is limited.

In conclusion, we observed that BMF was associated with future risk of multiple vascular and nonvascular diseases, including CVD, COPD, T2DM, and CKD. Our findings highlight that gastrointestinal health is related to the health of multiple systems and provide preliminary epidemiological evidence for potential systemic

For peer review only

effects of gut microbiota. Future studies, aiming at elucidating the mechanisms underlying the associations between gastrointestinal health and a number of systemic diseases, may be strongly facilitated by our findings.

Acknowledgements: The most important acknowledgment is to the participants in the study and the members of the survey teams in each of the 10 regional centers, as well as to the project development and management teams based at Beijing, Oxford and the 10 regional centers. The members of the steering committee and collaborative group are listed in the **Supplementary file**.

Contributors: JL and LL conceived and designed the paper. LL, ZC, and JC, as members of the CKB steering committee, designed and supervised the conduct of the whole study, obtained funding, and together with YG, ZB, LY, HD, YC, SYan, and YZ acquired the data. SYang, MF, CY, and JL analyzed the data. SYang and JL drafted the manuscript. CY, YG, ZB, MF, LY, HD, YC, SYan, YZ, JC, ZC, JL, and LL contributed to the interpretation of the results and critical revision of the manuscript for important intellectual content. All authors contributed to and approved the final manuscript. J.L. is the study guarantor.

Funding: This work was supported by grants (2016YFC0900500, 2016YFC0900501, 2016YFC0900504) from the National Key R&D Program of China. The CKB baseline survey and the first re-survey were supported by a grant from the Kadoorie Charitable Foundation in Hong Kong. The long-term follow-up is supported by grants from the UK Wellcome Trust (202922/Z/16/Z, 088158/Z/09/Z, 104085/Z/14/Z), National Natural Science Foundation of China (81390540, 81390541, 81390544), and Chinese Ministry of Science and Technology (2011BAI09B01). The funders had no role in the study design, data collection, data analysis and interpretation, writing of the report, or the decision to submit the article for publication.

Competing interests: None declared.

Patient consent: Obtained.

Ethics approval: The Ethical Review Committee of the Chinese Center for Disease Control and Prevention (Beijing, China) (Ethics ID number of approval: 005/2004) and the Oxford Tropical

For peer review only - http://bmjopen.bmj.com/site/about/guidelines.xhtml

Research Ethics Committee, University of Oxford (UK) (Ethics ID number of approval: OXTREC 025-04).

Data availability statement: The dataset supporting the conclusions of this article is available from the study website (http://www.ckbiobank.org), along with the access policy and procedures.

REFERENCES

1. Lee SB, Park G-M, Lee J-Y, et al. Association between non-alcoholic fatty liver disease and subclinical coronary atherosclerosis: An observational cohort study. *J Hepatol* 2018;68(5):1018-24. doi:10.1016/j.jhep.2017.12.012

2. Keller JJ, Wang J, Huang Y-L, et al. Increased risk of stroke among patients with ulcerative colitis: a population-based matched cohort study. *Int J Colorectal Dis* 2014;29(7):805-12. doi:10.1007/s00384-014-1862-6

3. Fan LL, Chen BH, Dai ZJ. The relation between gallstone disease and cardiovascular disease. *Sci Rep* 2017;7(1):15104. doi:10.1038/s41598-017-15430-5

4. Lv J, Yu C, Guo Y, et al. Gallstone Disease and the Risk of Type 2 Diabetes. *Sci Rep* 2017;7(1):15853. doi:10.1038/s41598-017-14801-2

5. Sinn DH, Kang D, Jang HR, et al. Development of chronic kidney disease in patients with non-alcoholic fatty liver disease: A cohort study. *J Hepatol* 2017;67(6):1274-80. doi:10.1016/j.jhep.2017.08.024

6. Lambeth SM, Carson T, Lowe J, et al. Composition, Diversity and Abundance of Gut Microbiome in Prediabetes and Type 2 Diabetes. *J Diabetes Obes* 2015;2(3):1-7. doi:10.15436/2376-0949.15.031

7. Yamashiro K, Tanaka R, Urabe T, et al. Gut dysbiosis is associated with metabolism and systemic inflammation in patients with ischemic stroke. *PLoS One* 2017;12(2):e0171521. doi:10.1371/journal.pone.0171521

8. Heianza Y, Ma W, Manson JE, et al. Gut Microbiota Metabolites and Risk of Major Adverse Cardiovascular Disease Events and Death: A Systematic Review and Meta-Analysis of Prospective Studies. *J Am Heart Assoc* 2017;6(7):e004947. doi:10.1161/JAHA.116.004947

9. Xu KY, Xia GH, Lu JQ, et al. Impaired renal function and dysbiosis of gut microbiota contribute to increased trimethylamine-N-oxide in chronic kidney disease patients. *Sci Rep* 2017;7(1):1445. doi:10.1038/s41598-017-01387-y

10. Pasini E, Aquilani R, Testa C, et al. Pathogenic Gut Flora in Patients With Chronic Heart Failure. *JACC Heart Fail* 2016;4(3):220-7. doi:10.1016/j.jchf.2015.10.009

11. Rao SS, Camilleri M, Hasler WL, et al. Evaluation of gastrointestinal transit in clinical practice: position paper of the American and European Neurogastroenterology and Motility Societies. *Neurogastroenterol Motil* 2011;23(1):8-23. doi:10.1111/j.1365-2982.2010.01612.x

12. Tornblom H, Van Oudenhove L, Sadik R, et al. Colonic Transit Time and IBS Symptoms: What's the Link. *Am J Gastroenterol* 2012;107(5):754-60. doi:10.1038/ajg.2012.5

13. Yang S, Shen Z, Yu C, et al. Association between the frequency of bowel movements and the risk of colorectal cancer in Chinese adults. *Chin J Epidemiol* 2019;40(4):382-88. doi:10.3760/cma.j.issn.0254-6450.2019.04.003

14. Sanjoaquin MA, Appleby PN, Spencer EA, et al. Nutrition and lifestyle in relation to bowel movement frequency: a cross-sectional study of 20,630 men and women in EPIC-Oxford. *Public Health Nutr* 2004;7(1):77-

For peer review only - http://bmjopen.bmj.com/site/about/guidelines.xhtml

83. doi:10.1079/Phn2003522
15. Nakaji S, Tokunaga S, Sakamoto J, et al. Relationship between lifestyle factors and defecation in a Japanese population. *Eur J Nutr* 2002;41(6):244-48. doi:10.1007/s00394-002-0380-4
16. Hadizadeh F, Walter S, Belheouane M, et al. Stool frequency is associated with gut microbiota composition. *Gut* 2017;66(3):559-60. doi:10.1136/gutjnl-2016-311935
17. Oki K, Toyama M, Banno T, et al. Comprehensive analysis of the fecal microbiota of healthy Japanese adults reveals a new bacterial lineage associated with a phenotype characterized by a high frequency of bowel movements and a lean body type. *BMC Microbiol* 2016;16(1):284. doi:10.1186/s12866-016-0898-x
18. Adams-Carr KL, Bestwick JP, Shribman S, et al. Constipation preceding Parkinson's disease: a systematic review and meta-analysis. *J Neurol Neurosurg Psychiatry* 2016;87(7):710-6. doi:10.1136/jnnp-2015-311680
19. Salmoirago-Blotcher E, Crawford S, Jackson E, et al. Constipation and risk of cardiovascular disease among postmenopausal women. *Am J Med* 2011;124(8):714-23. doi:10.1016/j.amjmed.2011.03.026
20. Honkura K, Tomata Y, Sugiyama K, et al. Defecation frequency and cardiovascular disease mortality in Japan: The Ohsaki cohort study. *Atherosclerosis* 2016:246:51-56. doi:10.1016/j.atherosclerosis.2016.01.007
21. Kubota Y, Iso H, Tamakoshi A, et al. Bowel Movement Frequency, Laxative Use, and Mortality From Coronary Heart Disease and Stroke Among Japanese Men and Women: The Japan Collaborative Cohort (JACC) Study. *J Epidemiol* 2016;26(5):242-48. doi:10.2188/jea.JE20150123
22. Ma WJ, Li YP, Heianza Y, et al. Associations of Bowel Movement Frequency with Risk of Cardiovascular Disease and Mortality among US Women. *Sci Rep* 2016:6:33005. doi:10.1038/srep33005
23. Sumida K, Molnar MZ, Potukuchi PK, et al. Constipation and risk of death and cardiovascular events. *Atherosclerosis* 2019;281:114-20. doi:10.1016/j.atherosclerosis.2018.12.021
24. Sumida K, Molnar MZ, Potukuchi PK, et al. Constipation and Incident CKD. *J Am Soc Nephrol* 2017;28(4):1248-58. doi:10.1681/ASN.2016060656
25. Chen ZM, Lee L, Chen JS, et al. Cohort profile: The Kadoorie study of chronic disease in China (KSCDC). *Int J Epidemiol* 2005;34(6):1243-49. doi:10.1093/ije/dyi1d74
26. Chen ZM, Chen JS, Collins R, et al. China Kadoorie Biobank of 0.5 million people: survey methods, baseline characteristics and long-term follow-up. *Int J Epidemiol* 2011;40(6):1652-66. doi:10.1093/ije/dyr120
27. Kurmi OP, Li LM, Wang J, et al. COPD and its association with smoking in the Mainland China: a cross-sectional analysis of 0.5 million men and women from ten diverse areas. *Int J Chronic Obstr* 2015;10:655-65. doi:10.2147/Copd.S75454
28. Li P. A Note on the Linearly and Quadratically Weighted Kappa Coefficients. *Psychometrika* 2016;81(3):795-801. doi:10.1007/s11336-016-9501-5
29. Du HD, Li LM, Whitlock G, et al. Patterns and socio-demographic correlates of domain-specific physical activities and their associations with adiposity in the China Kadoorie Biobank study. *Bmc Public Health* 2014;14:826. doi:10.1186/1471-2458-14-826
30. Shen Q, Yu C, Guo Y, et al. Habitual Tea Consumption and Risk of Fracture in 0.5 Million Chinese Adults: A Prospective Cohort Study. *Nutrients* 2018;10(11) doi:10.3390/nu10111633
31. Bragg F, Holmes MV, Iona A, et al. Association Between Diabetes and Cause-Specific Mortality in Rural and Urban Areas of China. *J Am Med Assoc* 2017;317(3):280-89. doi:10.1001/jama.2016.19720
32. Division of Disease Control, Ministry of Health of the People's Republic of China. Guideline for prevention and control of overweight and obesity in Chinese adults. Beijing: People's Medical Publishing House 2006.
33. Zhuang X, Tian Z, Li L, et al. Fecal Microbiota Alterations Associated With Diarrhea-Predominant Irritable

For peer review only - http://bmjopen.bmj.com/site/about/guidelines.xhtml

Bowel Syndrome. *Front Microbiol* 2018;9:1600. doi:10.3389/fmicb.2018.01600

34. Mancabelli L, Milani C, Lugli GA, et al. Unveiling the gut microbiota composition and functionality associated with constipation through metagenomic analyses. *Sci Rep* 2017;7(1):9879. doi:10.1038/s41598-017-10663-w

35. Tang WHW, Kitai T, Hazen SL. Gut Microbiota in Cardiovascular Health and Disease. *Circ Res* 2017;120(7):1183-96. doi:10.1161/Circresaha.117.309715

36. Budden KF, Gellatly SL, Wood DLA, et al. Emerging pathogenic links between microbiota and the gut-lung axis. *Nat Rev Microbiol* 2017;15(1):55-63. doi:10.1038/nrmicro.2016.142

37. Vermorken AJ, Andres E, Cui Y. Bowel movement frequency, oxidative stress and disease prevention. *Mol Clin Oncol* 2016;5(4):339-42. doi:10.3892/mco.2016.987

38. Pizzino G, Irrera N, Cucinotta M, et al. Oxidative Stress: Harms and Benefits for Human Health. *Oxid Med Cell Longev* 2017;2017:8416763. doi:10.1155/2017/8416763

39. Lv J, Qi L, Yu C, et al. Consumption of spicy foods and total and cause specific mortality: population based cohort study. *BMJ* 2015;351:h3942. doi:10.1136/bmj.h3942

40. Qin C, Lv J, Guo Y, et al. Associations of egg consumption with cardiovascular disease in a cohort study of 0.5 million Chinese adults. *Heart* 2018;104(21):1756-63. doi:10.1136/heartjnl-2017-312651

41. Du H, Li L, Bennett D, et al. Fresh Fruit Consumption and Major Cardiovascular Disease in China. *N Engl J Med* 2016;374(14):1332-43. doi:10.1056/NEJMoa1501451

42. Du H, Li L, Bennett D, et al. Fresh fruit consumption in relation to incident diabetes and diabetic vascular complications: A 7-y prospective study of 0.5 million Chinese adults. *PLoS Med* 2017;14(4):e1002279. doi:10.1371/journal.pmed.1002279

For peer review only

For peer review only - http://bmjopen.bmj.com/site/about/guidelines.xhtml

Table 1 | Baseline characteristics according to BMF at baseline for 487 198 participants

	More than once a day	Once a day	Once every 2-3 days	Less than 3 times a week
**Number of participants, n(%)**	46 426 (9.5)	373 054 (76.6)	46 570 (9.6)	21 148 (4.3)
**Women, %**	45.9	57.7	72.6	82.5
**Age, year**	53.0	51.5	50.6	51.0
**Urban area, %**	33.3	43.9	43.7	49.4
**Middle school and above, %**	46.7	49.5	49.1	48.4
**Agricultural and industrial workers, %**	58.2	57.4	57.9	57.5
**Household income ≥20 000 RMB/yr, %**	41.8	43.4	40.6	38.8
**Married, %**	91.4	91.0	90.2	89.2
**Current tobacco smoker, %**				
Men	68.2	67.5	70.4	72.5
Women	2.6	2.7	2.8	3.2
**Current daily alcohol drinker, %**				
Men	25.4	21.0	15.3	14.8
Women	1.0	1.0	0.8	0.7
**Physical activity, MET-h/d**	22.0	21.6	21.3	20.9
**Regular consumption of*, %**				
Red meat	47.0	47.4	46.9	45.3
Fresh vegetables	98.5	98.2	98.5	98.2
Fresh fruits	27.2	28.1	26.7	23.5
**Body mass index, kg/m^2**	24.5	23.6	22.8	22.6
**Waist circumference, cm**	82.4	80.2	77.9	77.3
**Hypertension, %**	38.8	34.2	27.5	25.7
**Diabetes, %**	6.0	5.2	5.5	6.4
**Family history of, %**				
Cancer	18.0	16.4	16.6	17.2
Diabetes	8.3	6.7	6.7	6.8
Heart attack	3.6	3.1	3.1	3.6
Stroke	19.4	17.5	17.0	17.2

BMF, bowel movement frequency; MET, metabolic equivalent of task.

Baseline characteristics were presented as mean or percentage. Age and sex were not adjusted, and others were adjusted for age, sex, and survey sites as appropriate. Linear trend was assessed by assigning consecutive integers to four BMF categories in a separate model. All *P* values for trend were <0.001, except for agricultural and industrial workers (*P*=0.359), regular consumption of fresh vegetables (*P*=0.955), prevalence of diabetes (*P*=0.024), family history of cancer (*P*=0.010) and heart attack (*P*=0.486).

*Regular consumption was defined as consumption at least 4-6 days per week.

For peer review only - http://bmjopen.bmj.com/site/about/guidelines.xhtml

Table 2 | HRs (95% CIs) for associations between BMF and multiple vascular and nonvascular diseases

Diseases	More than once a day	Once a day	Once every 2-3 days	Less than 3 times a week	*P* value for trend*
**Vascular diseases**					
**IHD**					
No. of cases	4668	30 470	3448	1761	
Cases/PYs (1/1000)	10.43	8.45	7.60	8.59	
Multivariable adjusted	1.12 (1.09-1.16)	1.00	1.00 (0.96-1.03)	1.07 (1.02-1.12)	0.008
**MCEs**					
No. of cases	871	5960	765	376	
Cases/PYs (1/1000)	1.88	1.61	1.64	1.78	
Multivariable adjusted	1.03 (0.96-1.11)	1.00	1.10 (1.02-1.19)	1.22 (1.10-1.36)	0.002
**Haemorrhagic stroke**					
No. of cases	1028	6856	723	336	
Cases/PYs (1/1000)	2.22	1.85	1.56	1.60	
Multivariable adjusted	0.97 (0.90-1.03)	1.00	0.98 (0.91-1.06)	1.07 (0.95-1.19)	0.256
**Ischaemic stroke**					
No. of cases	4044	28 594	3291	1650	
Cases/PYs (1/1000)	9.00	7.91	7.24	8.03	
Multivariable adjusted	1.01 (0.98-1.05)	1.00	1.04 (1.00-1.08)	1.10 (1.05-1.16)	0.002
**Heart failure**					
No. of cases	535	3068	398	203	
Cases/PYs (1/1000)	1.16	0.83	0.86	0.96	
Multivariable adjusted	1.33 (1.22-1.46)	1.00	0.96 (0.86-1.06)	0.99 (0.86-1.15)	<0.001
**Nonvascular diseases**					
**COPD**					
No. of cases	1518	8167	926	443	
Cases/PYs (1/1000)	3.63	2.37	2.14	2.29	
Multivariable adjusted	1.28 (1.22-1.36)	1.00	0.89 (0.83-0.95)	0.92 (0.84-1.02)	<0.001
**T2DM**					
No. of cases	2207	11 514	1077	483	
Cases/PYs (1/1000)	5.14	3.30	2.46	2.46	
Multivariable adjusted	1.20 (1.15-1.26)	1.00	0.86 (0.81-0.92)	0.84 (0.77-0.92)	<0.001
**CKD**					
No. of cases	821	4786	605	314	
Cases/PYs (1/1000)	1.81	1.31	1.33	1.52	
Multivariable adjusted	1.15 (1.07-1.24)	1.00	1.07 (0.98-1.17)	1.20 (1.07-1.35)	0.577

HR: hazard ratio; CI: confidence interval; BMF: bowel movement frequency; IHD: ischaemic heart disease; MCEs: major coronary events; COPD: chronic obstructive pulmonary disease; T2DM: type 2 diabetes mellitus; CKD: chronic kidney disease.

Cox regression models were stratified by 5-year groups and ten survey sites, with age as the underlying time scale.

Multivariable models were adjusted for sex (men or women); level of education (no formal school, primary school, middle school,

For peer review only

For peer review only - http://bmjopen.bmj.com/site/about/guidelines.xhtml

high school, college, or university or higher); occupation (agricultural, industrial, administrative or managerial, professional or technical, sales or service, retired, house wife or husband, self-employed, unemployed, other); household income (<2500, 2500-4999, 5000-9999, 10 000-19 999, 20 000-34 999, ≥35 000 RMB per year); marital status (married, widowed, divorced or separated, or never married); family history of certain diseases (presence, absence, or unclear; for corresponding disease only; no adjustment for heart failure, COPD, and CKD); smoking status (nonsmoker, former smoker, current smoker who smoked <15, 15-24, or ≥25 cigarettes or equivalents per day; those who had stopped smoking because of ill health were included in current smokers to avoid bias); total physical activity level (MET-h/day); alcohol consumption (non-drinker, former weekly drinker, weekly drinker, daily drinker with an intake of <15, 15-29, 30-59, or ≥60 g/day of pure alcohol); intake frequency of fresh vegetables, fresh fruit, and red meat (daily, 4-6 days/week, 1-3 days/week, monthly, or rarely or never); BMI (kg/m2); waist circumference (cm); prevalent hypertension and diabetes at baseline (presence or absence, no adjustment for T2DM).

*Assessed by assigning consecutive integers to four BMF categories in a separate model. A Bonferroni-corrected threshold was used, α=0.00625.

For peer review only

For peer review only - http://bmjopen.bmj.com/site/about/guidelines.xhtml

Table 3 | HRs (95% CIs) for associations between BMF and multiple vascular and nonvascular diseases stratified by obesity status

Diseases	Obesity subgroups	More than once a day		Once every 1-3 days*		Less than 3 times a week		*P* value for interaction†
		Cases	*HR (95%CI)*	Cases	*HR (95%CI)*	Cases	*HR (95%CI)*	
**Vascular diseases**								
**IHD**								0.383
	No	2771	1.15 (1.10-1.19)	22 109	1.00	1258	1.04 (0.98-1.11)	
	Yes	1897	1.10 (1.04-1.15)	11 809	1.00	503	1.13 (1.03-1.23)	
**MCEs**								0.001
	No	577	1.07 (0.98-1.17)	4478	1.00	238	1.07 (0.93-1.22)	
	Yes	294	0.91 (0.81-1.03)	2247	1.00	138	1.57 (1.31-1.86)	
**Haemorrhagic stroke**								0.624
	No	706	0.98 (0.90-1.06)	5543	1.00	269	1.08 (0.96-1.23)	
	Yes	322	0.93 (0.82-1.04)	2036	1.00	67	1.05 (0.82-1.34)	
**Ischaemic stroke**								0.688
	No	2367	1.02 (0.98-1.07)	20 903	1.00	1221	1.10 (1.03-1.16)	
	Yes	1677	1.01 (0.96-1.06)	10 982	1.00	429	1.07 (0.97-1.18)	
**Heart failure**								0.356
	No	360	1.36 (1.22-1.52)	2493	1.00	152	0.96 (0.81-1.13)	
	Yes	175	1.25 (1.06-1.47)	973	1.00	51	1.22 (0.92-1.62)	
**Nonvascular diseases**								
**COPD**								0.353
	No	1132	1.29 (1.21-1.37)	7070	1.00	371	0.98 (0.88-1.09)	
	Yes	386	1.21 (1.08-1.35)	2023	1.00	72	0.90 (0.71-1.14)	
**T2DM**								0.506
	No	1048	1.35 (1.27-1.44)	7069	1.00	338	0.80 (0.72-0.90)	

For peer review only

For peer review only - http://bmjopen.bmj.com/site/about/guidelines.xhtml

Diseases	Obesity	More than once a day		Once every 1-3 days*		Less than 3 times a week		*P* value for
	Yes	1159	1.25 (1.17-1.33)	5522	1.00	142	0.77 (0.65-0.91)	
**CKD**								0.051
	No	545	1.22 (1.11-1.33)	3789	1.00	252	1.23 (1.08-1.40)	
	Yes	276	1.01 (0.88-1.15)	1602	1.00	62	1.05 (0.81-1.35)	

HR: hazard ratio; CI: confidence interval; BMF: bowel movement frequency; IHD: ischaemic heart disease; MCEs: major coronary events; COPD: chronic obstructive pulmonary disease; T2DM: type 2 diabetes mellitus; CKD: chronic kidney disease; BMI: body mass index; WC: waist circumference.

Multivariable models adjusted for the same set of covariates as Table 2, except for BMI (kg/m2) and waist circumference (cm). Obesity status was defined according to BMI and WC, with "yes" as BMI≥28.0 kg/m^2 or WC≥90.0 cm (men)/≥85.0 cm (women).

*Combined group including "once a day" and "once every 2-3 days".

†A Bonferroni-corrected threshold was used, α=0.00625.

For peer review only - http://bmjopen.bmj.com/site/about/guidelines.xhtml

For peer review only

Supplementary Information

Bowel movement frequency and risks of major vascular and nonvascular diseases: population based cohort study among Chinese adults

Yang, S. *et al*

Supplementary Tables 1
Supplementary Table 1 | HRs (95% CIs) for associations between BMF and multiple vascular and nonvascular diseases with stepwise adjustment...1
Supplementary Table 2 | HRs (95% CIs) for associations between BMF and multiple vascular and nonvascular diseases stratified by sex...3
Supplementary Table 3 | HRs (95% CIs) for associations between BMF and multiple vascular and nonvascular diseases stratified by baseline age 5
Supplementary Table 4 | HRs (95% CIs) for associations between BMF and multiple vascular and nonvascular diseases stratified by baseline hypertension 7

Supplementary Note 9
1. CKB collaborative group 9
2. Food frequency questionnaire..11

For peer review only

Supplementary Tables

Supplementary Table 1 | HRs (95% CIs) for associations between BMF and multiple vascular and nonvascular diseases with stepwise adjustment

Diseases	More than once a day	Once a day	Once every 2-3 days	Less than 3 times a week	*P* value for trend*
**Vascular diseases**					
**IHD**					
No. of participants	46 426	373 054	46 570	21 148	
No. of cases	4668	30 470	3448	1761	
Cases/PYs (1/1000)	10.43	8.45	7.60	8.59	
Model 1	1.16 (1.13-1.20)	1.00	0.96 (0.93-0.99)	1.03 (0.98-1.08)	<0.001
Model 2	1.16 (1.13-1.20)	1.00	0.96 (0.93-1.00)	1.03 (0.98-1.08)	<0.001
Model 3	1.17 (1.13-1.20)	1.00	0.95 (0.92-0.99)	1.01 (0.96-1.06)	<0.001
Model 4	1.12 (1.09-1.16)	1.00	1.00 (0.96-1.03)	1.07 (1.02-1.12)	0.008
**MCEs**					
No. of participants	46 426	373 054	46 570	21 148	
No. of cases	871	5960	765	376	
Cases/PYs (1/1000)	1.88	1.61	1.64	1.78	
Model 1	1.05 (0.98-1.13)	1.00	1.12 (1.04-1.21)	1.28 (1.16-1.43)	<0.001
Model 2	1.05 (0.98-1.13)	1.00	1.09 (1.01-1.18)	1.23 (1.10-1.36)	0.005
Model 3	1.05 (0.98-1.13)	1.00	1.08 (1.00-1.16)	1.19 (1.07-1.32)	0.023
Model 4	1.03 (0.96-1.11)	1.00	1.10 (1.02-1.19)	1.22 (1.10-1.36)	0.002
**Haemorrhagic stroke**					
No. of participants	46 426	373 054	46 570	21 148	
No. of cases	1028	6856	723	336	
Cases/PYs (1/1000)	2.22	1.85	1.56	1.60	
Model 1	1.02 (0.95-1.09)	1.00	0.95 (0.88-1.03)	1.03 (0.92-1.15)	0.576
Model 2	1.01 (0.94-1.08)	1.00	0.93 (0.86-1.01)	1.00 (0.89-1.11)	0.306
Model 3	0.99 (0.93-1.06)	1.00	0.93 (0.86-1.01)	0.98 (0.88-1.10)	0.340
Model 4	0.97 (0.90-1.03)	1.00	0.98 (0.91-1.06)	1.07 (0.95-1.19)	0.256
**Ischaemic stroke**					
No. of participants	46 426	373 054	46 570	21 148	
No. of cases	4044	28 594	3291	1650	
Cases/PYs (1/1000)	9.00	7.91	7.24	8.03	
Model 1	1.07 (1.04-1.11)	1.00	0.99 (0.96-1.03)	1.05 (1.00-1.10)	0.178
Model 2	1.07 (1.03-1.10)	1.00	0.99 (0.95-1.03)	1.04 (0.99-1.09)	0.135
Model 3	1.06 (1.03-1.10)	1.00	0.99 (0.95-1.02)	1.03 (0.98-1.08)	0.073
Model 4	1.01 (0.98-1.05)	1.00	1.04 (1.00-1.08)	1.10 (1.05-1.16)	0.002
**Heart failure**					
No. of participants	46 426	373 054	46 570	21 148	
No. of cases	535	3068	398	203	

For peer review only - http://bmjopen.bmj.com/site/about/guidelines.xhtml

Diseases	More than once a day	Once a day	Once every 2-3 days	Less than 3 times a week	*P* value for trend*
Cases/PYs (1/1000)	1.16	0.83	0.86	0.96	
Model 1	1.35 (1.23-1.48)	1.00	0.98 (0.88-1.09)	1.04 (0.90-1.21)	<0.001
Model 2	1.34 (1.23-1.48)	1.00	0.97 (0.87-1.08)	1.02 (0.88-1.17)	<0.001
Model 3	1.35 (1.23-1.48)	1.00	0.95 (0.85-1.05)	0.98 (0.85-1.13)	<0.001
Model 4	1.33 (1.22-1.46)	1.00	0.96 (0.86-1.06)	0.99 (0.86-1.15)	<0.001
**Nonvascular diseases**					
**COPD**					
No. of participants	42 122	347 771	43 350	19 414	
No. of cases	1518	8167	926	443	
Cases/PYs (1/1000)	3.63	2.37	2.14	2.29	
Model 1	1.26 (1.19-1.33)	1.00	0.93 (0.87-1.00)	1.00 (0.91-1.11)	<0.001
Model 2	1.25 (1.19-1.33)	1.00	0.92 (0.86-0.99)	0.97 (0.88-1.07)	<0.001
Model 3	1.26 (1.19-1.34)	1.00	0.91 (0.85-0.97)	0.94 (0.86-1.04)	<0.001
Model 4	1.28 (1.22-1.36)	1.00	0.89 (0.83-0.95)	0.92 (0.84-1.02)	<0.001
**T2DM**					
No. of participants	43 703	353 573	44 037	19 724	
No. of cases	2207	11 514	1077	483	
Cases/PYs (1/1000)	5.14	3.30	2.46	2.46	
Model 1	1.44 (1.38-1.51)	1.00	0.73 (0.69-0.78)	0.68 (0.62-0.75)	<0.001
Model 2	1.42 (1.36-1.49)	1.00	0.73 (0.69-0.78)	0.68 (0.62-0.75)	<0.001
Model 3	1.43 (1.36-1.50)	1.00	0.73 (0.69-0.78)	0.68 (0.62-0.74)	<0.001
Model 4	1.20 (1.15-1.26)	1.00	0.86 (0.81-0.92)	0.84 (0.77-0.92)	<0.001
**CKD**					
No. of participants	45 706	368 119	45 846	20 749	
No. of cases	821	4786	605	314	
Cases/PYs (1/1000)	1.81	1.31	1.33	1.52	
Model 1	1.20 (1.11-1.29)	1.00	1.05 (0.96-1.14)	1.20 (1.07-1.35)	0.754
Model 2	1.20 (1.11-1.29)	1.00	1.05 (0.96-1.14)	1.20 (1.07-1.35)	0.755
Model 3	1.20 (1.12-1.30)	1.00	1.03 (0.95-1.12)	1.16 (1.04-1.31)	0.360
Model 4	1.15 (1.07-1.24)	1.00	1.07 (0.98-1.17)	1.20 (1.07-1.35)	0.577

HR: hazard ratio; CI: confidence interval; BMF: bowel movement frequency; IHD: ischaemic heart disease; MCEs: major coronary events; COPD: chronic obstructive pulmonary disease; T2DM: type 2 diabetes mellitus; CKD: chronic kidney disease.

Model 1 was adjusted for sex.

Model 2 was additionally adjusted for level of education, occupation, household income, marital status, smoking status, and total physical activity level, and family history (for corresponding disease only; no adjustment for heart failure, COPD, and CKD).

Model 3 was additionally adjusted for alcohol consumption, and intake frequency of fresh vegetables, fresh fruits, and red meat.

Model 4 was additionally adjusted for body mass index, waist circumference, prevalent hypertension and diabetes at baseline (no adjustment for T2DM).

*Assessed by assigning consecutive integers to four BMF categories in a separate model. A Bonferroni-corrected threshold was used, α=0.00625.

For peer review only - http://bmjopen.bmj.com/site/about/guidelines.xhtml

Supplementary Table 2 | HRs (95% CIs) for associations between BMF and multiple vascular and nonvascular diseases stratified by sex

Diseases	Subgroups	More than once a day		Once every 1-3 days*		Less than 3 times a week		*P* value for interaction†
		Cases	*HR (95%CI)*	Cases	*HR (95%CI)*	Cases	*HR (95%CI)*	
**Vascular diseases**								
**IHD**								0.099
	Men	2335	1.09 (1.04-1.14)	13 822	1.00	385	1.11 (1.00-1.23)	
	Women	2333	1.16 (1.11-1.21)	20 096	1.00	1376	1.05 (0.99-1.11)	
**MCEs**								0.756
	Men	553	1.00 (0.91-1.09)	3762	1.00	137	1.26 (1.06-1.50)	
	Women	318	1.05 (0.93-1.18)	2963	1.00	239	1.20 (1.05-1.37)	
**Haemorrhagic stroke**								0.322
	Men	645	0.97 (0.89-1.05)	3950	1.00	117	1.19 (0.99-1.43)	
	Women	383	0.96 (0.86-1.07)	3629	1.00	219	1.01 (0.88-1.16)	
**Ischaemic stroke**								0.006
	Men	2234	1.01 (0.97-1.06)	14 229	1.00	460	1.25 (1.13-1.37)	
	Women	1810	1.01 (0.96-1.06)	17 656	1.00	1190	1.04 (0.98-1.11)	
**Heart failure**								0.559
	Men	268	1.26 (1.11-1.44)	1575	1.00	53	0.99 (0.75-1.30)	
	Women	267	1.42 (1.25-1.62)	1891	1.00	150	0.98 (0.83-1.16)	
**Nonvascular diseases**								
**COPD**								0.212
	Men	885	1.25 (1.16-1.35)	4390	1.00	120	1.00 (0.83-1.20)	
	Women	633	1.37 (1.26-1.49)	4703	1.00	323	0.89 (0.80-1.00)	
**T2DM**								0.997
	Men	1035	1.20 (1.12-1.29)	4721	1.00	74	0.89 (0.70-1.12)	
	Women	1172	1.22 (1.15-1.30)	7870	1.00	409	0.84 (0.76-0.93)	
**CKD**								0.249
	Men	423	1.13 (1.01-1.25)	2250	1.00	75	1.38 (1.09-1.74)	
	Women	398	1.16 (1.05-1.29)	3141	1.00	239	1.13 (0.99-1.29)	

For peer review only - http://bmjopen.bmj.com/site/about/guidelines.xhtml

HR: hazard ratio; CI: confidence interval; BMF: bowel movement frequency; IHD: ischaemic heart disease; MCEs: major coronary events; COPD: chronic obstructive pulmonary disease; T2DM: type 2 diabetes mellitus; CKD: chronic kidney disease.

Multivariable models adjusted for same set of covariates as Table 2, except for the stratified variable of interest.

* Combined group including “once a day” and “once every 2-3 days”.

† A Bonferroni-corrected threshold was used, α=0.00625.

For peer review only

Supplementary Table 3 | HRs (95% CIs) for associations between BMF and multiple vascular and nonvascular diseases stratified by baseline age

Diseases	Subgroups	More than once a day		Once every 1-3 days*		Less than 3 times a week		*P* value for interaction†
		Cases	*HR (95%CI)*	Cases	*HR (95%CI)*	Cases	*HR (95%CI)*	
**Vascular diseases**								
**IHD**								0.362
	<60 yr	2531	1.14 (1.09-1.19)	18 064	1.00	810	1.04 (0.97-1.12)	
	⩾60 yr	2137	1.10 (1.06-1.16)	15 854	1.00	951	1.09 (1.02-1.17)	
**MCEs**								0.014
	<60 yr	357	1.08 (0.96-1.21)	2377	1.00	81	1.08 (0.86-1.35)	
	⩾60 yr	514	0.97 (0.88-1.06)	4348	1.00	295	1.25 (1.11-1.41)	
**Haemorrhagic stroke**								0.094
	<60 yr	521	0.96 (0.87-1.05)	3568	1.00	106	0.93 (0.77-1.14)	
	⩾60 yr	507	0.97 (0.88-1.06)	4011	1.00	230	1.15 (1.01-1.32)	
**Ischaemic stroke**								0.902
	<60 yr	2212	1.02 (0.97-1.06)	16 347	1.00	717	1.08 (1.00-1.16)	
	⩾60 yr	1832	1.00 (0.95-1.05)	15 538	1.00	933	1.11 (1.04-1.19)	
**Heart failure**								0.761
	<60 yr	204	1.38 (1.19-1.61)	1269	1.00	52	0.88 (0.66-1.16)	
	⩾60 yr	331	1.31 (1.16-1.47)	2197	1.00	151	1.04 (0.88-1.23)	
**Nonvascular diseases**								
**COPD**								0.309
	<60 yr	706	1.27 (1.17-1.38)	4180	1.00	182	0.96 (0.82-1.11)	
	⩾60 yr	812	1.33 (1.24-1.44)	4913	1.00	261	0.91 (0.81-1.04)	
**T2DM**								0.331
	<60 yr	1549	1.19 (1.13-1.26)	8826	1.00	308	0.83 (0.74-0.94)	
	⩾60 yr	658	1.25 (1.15-1.36)	3765	1.00	175	0.92 (0.79-1.07)	
**CKD**								0.567
	<60 yr	537	1.20 (1.10-1.32)	3446	1.00	189	1.19 (1.02-1.38)	

For peer review only

Diseases	Subgroups	More than once a day		Once every 1-3 days*		Less than 3 times a week		*P* value for interaction†
		Cases	*HR (95%CI)*	Cases	*HR (95%CI)*	Cases	*HR (95%CI)*	
	⩾60 yr	284	1.06 (0.94-1.20)	1945	1.00	125	1.17 (0.98-1.41)	

HR: hazard ratio; CI: confidence interval; BMF: bowel movement frequency; IHD: ischaemic heart disease; MCEs: major coronary events; COPD: chronic obstructive pulmonary disease; T2DM: type 2 diabetes mellitus; CKD: chronic kidney disease.

Multivariable models adjusted for same set of covariates as Table 2, except for the stratified variable of interest.

* Combined group including “once a day” and “once every 2-3 days”.

† A Bonferroni-corrected threshold was used, α=0.00625.

For peer review only

Supplementary Table 4 | HRs (95% CIs) for associations between BMF and multiple vascular and nonvascular diseases stratified by baseline hypertension

Diseases	Subgroups	More than once a day		Once every 1-3 days*		Less than 3 times a week		*P* value for interaction†
		Cases	*HR (95%CI)*	Cases	*HR (95%CI)*	Cases	*HR (95%CI)*	
**Vascular diseases**								
**IHD**								0.192
	No	2019	1.17 (1.12-1.23)	16 300	1.00	995	1.07 (1.00-1.14)	
	Yes	2649	1.09 (1.04-1.13)	17 618	1.00	766	1.06 (0.99-1.14)	
**MCEs**								0.102
	No	328	1.12 (1.00-1.26)	2498	1.00	157	1.22 (1.04-1.44)	
	Yes	543	0.96 (0.88-1.05)	4227	1.00	219	1.20 (1.05-1.38)	
**Haemorrhagic stroke**								0.059
	No	266	1.00 (0.87-1.13)	2108	1.00	128	1.17 (0.98-1.41)	
	Yes	762	0.95 (0.88-1.03)	5471	1.00	208	1.01 (0.88-1.17)	
**Ischaemic stroke**								0.134
	No	1441	1.03 (0.97-1.09)	13 429	1.00	845	1.10 (1.02-1.18)	
	Yes	2603	0.99 (0.95-1.03)	18 456	1.00	805	1.09 (1.02-1.17)	
**Heart failure**								0.432
	No	230	1.47 (1.27-1.69)	1570	1.00	102	0.95 (0.78-1.16)	
	Yes	305	1.25 (1.10-1.41)	1896	1.00	101	1.05 (0.85-1.28)	
**Nonvascular diseases**								
**COPD**								0.654
	No	779	1.29 (1.20-1.40)	5191	1.00	278	0.92 (0.81-1.04)	
	Yes	739	1.30 (1.20-1.41)	3902	1.00	165	0.96 (0.82-1.13)	
**T2DM**								0.011
	No	925	1.26 (1.18-1.36)	6063	1.00	264	0.82 (0.72-0.93)	
	Yes	1282	1.16 (1.10-1.24)	6528	1.00	219	0.96 (0.84-1.10)	
**CKD**								0.255
	No	355	1.20 (1.08-1.35)	2605	1.00	170	1.12 (0.96-1.32)	

For peer review only - http://bmjopen.bmj.com/site/about/guidelines.xhtml

For peer review only

Diseases	Subgroups	More than once a day		Once every 1-3 days*		Less than 3 times a week		*P* value for interaction†
		Cases	*HR (95%CI)*	Cases	*HR (95%CI)*	Cases	*HR (95%CI)*	
	Yes	466	1.10 (0.99-1.21)	2786	1.00	144	1.29 (1.09-1.53)	

HR: hazard ratio; CI: confidence interval; BMF: bowel movement frequency; IHD: ischaemic heart disease; MCEs: major coronary events; COPD: chronic obstructive pulmonary disease; T2DM: type 2 diabetes mellitus; CKD: chronic kidney disease..

Multivariable models adjusted for same set of covariates as Table 2, except for the stratified variable of interest.

* Combined group including "once a day" and "once every 2-3 days".

† A Bonferroni-corrected threshold was used, α=0.00625.

For peer review only

For peer review only - http://bmjopen.bmj.com/site/about/guidelines.xhtml

Supplementary Note

1. CKB collaborative group

International Steering Committee: Junshi Chen, Zhengming Chen (PI), Robert Clarke, Rory Collins, Yu Guo, Liming Li (PI), Jun Lv, Richard Peto, Robin Walters. **International Co-ordinating Centre, Oxford:** Daniel Avery, Ruth Boxall, Derrick Bennett, Yumei Chang, Yiping Chen, Zhengming Chen, Robert Clarke, Huaidong Du, Simon Gilbert, Alex Hacker, Mike Hill, Michael Holmes, Andri Iona, Christiana Kartsonaki, Rene Kerosi, Ling Kong, Om Kurmi, Garry Lancaster, Sarah Lewington, Kuang Lin, John McDonnell, Iona Millwood, Qunhua Nie, Jayakrishnan Radhakrishnan, Paul Ryder, Sam Sansome, Dan Schmidt, Paul Sherliker, Rajani Sohoni, Becky Stevens, Iain Turnbull, Robin Walters, Jenny Wang, Lin Wang, Neil Wright, Ling Yang, Xiaoming Yang. **National Co-ordinating Centre, Beijing:** Zheng Bian, Yu Guo, Xiao Han, Can Hou, Jun Lv, Pei Pei, Chao Liu, Yunlong Tan, Canqing Yu. **10 Regional Co-ordinating Centres: Qingdao CDC:** Zengchang Pang, Ruqin Gao, Shanpeng Li, Shaojie Wang, Yongmei Liu, Ranran Du, Yajing Zang, Liang Cheng, Xiaocao Tian, Hua Zhang, Yaoming Zhai, Feng Ning, Xiaohui Sun, Feifei Li. **Licang CDC:** Silu Lv, Junzheng Wang, Wei Hou. **Heilongjiang Provincial CDC:** Mingyuan Zeng, Ge Jiang, Xue Zhou. **Nangang CDC:** Liqiu Yang, Hui He, Bo Yu, Yanjie Li, Qinai Xu,Quan Kang, Ziyan Guo. **Hainan Provincial CDC:** Dan Wang, Ximin Hu, Jinyan Chen, Yan Fu, Zhenwang Fu, Xiaohuan Wang. **Meilan CDC:** Min Weng, Zhendong Guo, Shukuan Wu,Yilei Li, Huimei Li, Zhifang Fu. **Jiangsu Provincial CDC:** Ming Wu, Yonglin Zhou, Jinyi Zhou, Ran Tao, Jie Yang, Jian Su. **Suzhou CDC:** Fang liu, Jun Zhang, Yihe Hu, Yan Lu, , Liangcai Ma, Aiyu Tang, Shuo Zhang, Jianrong Jin, Jingchao Liu. **Guangxi Provincial CDC:** Zhenzhu Tang, Naying Chen, Ying Huang. **Liuzhou CDC:** Mingqiang Li, Jinhuai Meng, Rong Pan, Qilian Jiang, Jian Lan,Yun Liu, Liuping Wei, Liyuan Zhou, Ningyu Chen Ping Wang, Fanwen Meng, Yulu Qin,, Sisi Wang. **Sichuan Provincial CDC:** Xianping Wu,

For peer review only - http://bmjopen.bmj.com/site/about/guidelines.xhtml

Ningmei Zhang, Xiaofang Chen,Weiwei Zhou. **Pengzhou CDC:** Guojin Luo, Jianguo Li, Xiaofang Chen, Xunfu Zhong, Jiaqiu Liu, Qiang Sun. **Gansu Provincial CDC:** Pengfei Ge, Xiaolan Ren, Caixia Dong. **Maiji CDC:** Hui Zhang, Enke Mao, Xiaoping Wang, Tao Wang, Xi zhang. **Henan Provincial CDC:** Ding Zhang, Gang Zhou, Shixian Feng, Liang Chang, Lei Fan. **Huixian CDC:** Yulian Gao, Tianyou He, Huarong Sun, Pan He, Chen Hu, Xukui Zhang, Huifang Wu, Pan He. **Zhejiang Provincial CDC:** Min Yu, Ruying Hu, Hao Wang. Tongxiang CDC: Yijian Qian, Chunmei Wang, Kaixu Xie, Lingli Chen, Yidan Zhang, Dongxia Pan, Qijun Gu. **Hunan Provincial CDC:** Yuelong Huang, Biyun Chen, Li Yin, Huilin Liu, Zhongxi Fu, Qiaohua Xu. **Liuyang CDC:** Xin Xu, Hao Zhang, Huajun Long, Xianzhi Li, Libo Zhang, Zhe Qiu.

For peer review only

For peer review only - http://bmjopen.bmj.com/site/about/guidelines.xhtml

2. Food frequency questionnaire

Section 5: Diet

5.1 During the past 12 months, about how often did you eat the following foods?

	Daily	4-6 days per week	1-3 days per week	Monthly	Never /rarely
Rice					
Wheat					
Other staple food (corn, millet etc.)					
Meat					
Poultry					
Fish/sea food					
Fresh eggs					
Fresh vegetables					
Soybean products					
Preserved vegetables					
Fresh fruit					
Dairy products (milk, yogurt)					

5.2 During the past 12 months, have you taken the following supplements regularly?

Yes	No	
		Fish oil/cod liver oil
		Vitamins
		Calcium/iron/zinc
		Ginseng (at least 5 or more times during a year)
		Other herbal products

(5.3 to 5.6 are omitted)

5.7 During the past month, about how often did you eat hot spicy food?

- ☐ Never or almost never → Go to section 6
- ☐ Only occasionally → Go to section 6
- ☐ 1-2 days/week
- ☐ 3-5 days/week
- ☐ Daily or almost every day

The complete questionnaire is available below:
https://www.ckbiobank.org/site/Study+Resources/Baseline+Data

The categories of covariates in Cox models: intake frequency of rice, wheat, other staple food (daily, 4-6 days/week, 1-3 days/week, monthly, or rarely or never); spicy food (6-7, 3-5, 1-2 days/week, occasionally, never); consumption of nutritional supplements (fish oil/cod liver oil, vitamins, calcium/iron/zinc, ginseng and other herbal products; yes or no);

For peer review only - http://bmjopen.bmj.com/site/about/guidelines.xhtml

STROBE Statement—Checklist of items that should be included in reports of ***cohort studies***

	Item No	Recommendation	Page No
**Title and abstract**	1	(*a*) Indicate the study's design with a commonly used term in the title or the abstract	1
		(*b*) Provide in the abstract an informative and balanced summary of what was done and what was found	2-3
**Introduction**			
Background/rationale	2	Explain the scientific background and rationale for the investigation being reported	4
Objectives	3	State specific objectives, including any prespecified hypotheses	5
**Methods**			
Study design	4	Present key elements of study design early in the paper	5
Setting	5	Describe the setting, locations, and relevant dates, including periods of recruitment, exposure, follow-up, and data collection	5-7
Participants	6	(*a*) Give the eligibility criteria, and the sources and methods of selection of participants. Describe methods of follow-up	5-7
		(*b*) For matched studies, give matching criteria and number of exposed and unexposed	N/A
Variables	7	Clearly define all outcomes, exposures, predictors, potential confounders, and effect modifiers. Give diagnostic criteria, if applicable	6-8
Data sources/ measurement	8*	For each variable of interest, give sources of data and details of methods of assessment (measurement). Describe comparability of assessment methods if there is more than one group	6, 7
Bias	9	Describe any efforts to address potential sources of bias	6, 8, 9
Study size	10	Explain how the study size was arrived at	5
Quantitative variables	11	Explain how quantitative variables were handled in the analyses. If applicable, describe which groupings were chosen and why	8
Statistical methods	12	(*a*) Describe all statistical methods, including those used to control for confounding	8, 9
		(*b*) Describe any methods used to examine subgroups and interactions	9
		(*c*) Explain how missing data were addressed	5
		(*d*) If applicable, explain how loss to follow-up was addressed	7
		(*e*) Describe any sensitivity analyses	9
**Results**			
Participants	13*	(a) Report numbers of individuals at each stage of study—eg numbers potentially eligible, examined for eligibility, confirmed eligible, included in the study, completing follow-up, and analysed	6, 9
		(b) Give reasons for non-participation at each stage	N/A
		(c) Consider use of a flow diagram	—
Descriptive data	14*	(a) Give characteristics of study participants (eg demographic, clinical, social) and information on exposures and potential confounders	10
		(b) Indicate number of participants with missing data for each variable of interest	N/A
		(c) Summarise follow-up time (eg, average and total amount)	10
Outcome data	15*	Report numbers of outcome events or summary measures over time	10

For peer review only - http://bmjopen.bmj.com/site/about/guidelines.xhtml

Main results	16	(*a*) Give unadjusted estimates and, if applicable, confounder-adjusted estimates and their precision (eg, 95% confidence interval). Make clear which confounders were adjusted for and why they were included	8, 11
		(*b*) Report category boundaries when continuous variables were categorized	9
		(*c*) If relevant, consider translating estimates of relative risk into absolute risk for a meaningful time period	—
Other analyses	17	Report other analyses done—eg analyses of subgroups and interactions, and sensitivity analyses	11
**Discussion**			
Key results	18	Summarise key results with reference to study objectives	12
Limitations	19	Discuss limitations of the study, taking into account sources of potential bias or imprecision. Discuss both direction and magnitude of any potential bias	16
Interpretation	20	Give a cautious overall interpretation of results considering objectives, limitations, multiplicity of analyses, results from similar studies, and other relevant evidence	12-14
Generalisability	21	Discuss the generalisability (external validity) of the study results	16
**Other information**			
Funding	22	Give the source of funding and the role of the funders for the present study and, if applicable, for the original study on which the present article is based	17

*Give information separately for exposed and unexposed groups.

Note: An Explanation and Elaboration article discusses each checklist item and gives methodological background and published examples of transparent reporting. The STROBE checklist is best used in conjunction with this article (freely available on the Web sites of PLoS Medicine at http://www.plosmedicine.org/, Annals of Internal Medicine at http://www.annals.org/, and Epidemiology at http://www.epidem.com/). Information on the STROBE Initiative is available at http://www.strobe-statement.org.

For peer review only

For peer review only - http://bmjopen.bmj.com/site/about/guidelines.xhtml

BMJ Open

Bowel movement frequency and risks of major vascular and nonvascular diseases: a population-based cohort study among Chinese adults

Journal:	*BMJ Open*
Manuscript ID	bmjopen-2019-031028.R2
Article Type:	Original research
Date Submitted by the Author:	25-Nov-2019
Complete List of Authors:	Yang, Songchun; Peking University Health Science Center, Epidemiology & Biostatistics<br>Yu, Canqing; Peking University Health Science Center, Epidemiology & Biostatistics<br>Guo, Yu; Chinese Academy of Medical Sciences,<br>Bian, Zheng; Chinese Academy of Medical Sciences,<br>Fan, Mengyu; Peking University Health Science Center, Epidemiology & Biostatistics<br>Yang, Ling; University of Oxford, Nuffield Department of Population Health<br>Du, Huaidong; University of Oxford, Nuffield Department of Population Health<br>Chen, Yiping; University of Oxford, Nuffield Department of Population Health<br>Yan, Shichun; Heilongjiang Center for Disease Control and Prevention<br>Zang, Yajing; Qingdao Center for Disease Control and Prevention<br>Chen, Junshi; China National Center for Food Safety Risk Assessment<br>Chen, Zhengming; University of Oxford, Nuffield Department of Population Health<br>Lv, Jun; Peking University Health Science Center, Epidemiology & Biostatistics<br>LI, Li-ming; Peking University School of Public Health, Epidemiology and Biostatistics
**Primary Subject Heading**:	Epidemiology
Secondary Subject Heading:	Public health, Cardiovascular medicine, Diabetes and endocrinology, Renal medicine, Respiratory medicine
Keywords:	EPIDEMIOLOGY, cohort study, chronic diseases, PUBLIC HEALTH

SCHOLARONE™
Manuscripts

Bowel movement frequency and risks of major vascular and nonvascular diseases: a population-based cohort study among Chinese adults

Songchun Yang [1], Canqing Yu [1] , Yu Guo [2], Zheng Bian [2], Mengyu Fan [1], Ling Yang [3,4], Huaidong Du [3,4], Yiping Chen [3,4], Shichun Yan [5], Yajing Zang [6], Junshi Chen [7], Zhengming Chen [4], Jun Lv [1,8,9] *, Liming Li [1] and on behalf of the China Kadoorie Biobank Collaborative Group †

[1] Department of Epidemiology and Biostatistics, School of Public Health, Peking University Health Science Centre, Beijing 100191, China;

[2] Chinese Academy of Medical Sciences, Beijing 100730, China;

[3] Medical Research Council Population Health Research Unit, Nuffield Department of Population Health, University of Oxford, Oxford OX3 7LF, United Kingdom;

[4] Clinical Trial Service Unit and Epidemiological Studies Unit, Nuffield Department of Population Health, University of Oxford, Oxford OX3 7LF, United Kingdom;

[5] NCDs Prevention and Control Department, Heilongjiang Centre for Disease Control and Prevention, Harbin 150030, Heilongjiang, China;

[6] Qingdao Centre for Disease Control and Prevention, Qingdao 266033, Shandong, China;

[7] China National Centre for Food Safety Risk Assessment, Beijing 100738, China;

[8] Key Laboratory of Molecular Cardiovascular Sciences (Peking University), Ministry of Education, Beijing 100191, China

[9] Peking University Institute of Environmental Medicine, Beijing 100191, China

† The members of steering committee and collaborative group are listed in the supplementary file.

Address for correspondence

Jun Lv

Department of Epidemiology and Biostatistics

School of Public Health

Peking University Health Science Centre

38 Xueyuan Road, Haidian District

Beijing 100191

China

Email: lvjun@bjmu.edu.cn

Tel: +86-10-82801528 (ext. 322)

Word count: Abstract 273, Main text 3295

(3 tables, 4 supplementary tables, and 1 supplementary file)

25 Nov 2019

For peer review only

ABSTRACT

Objective: The application of bowel movement frequency (BMF) in primary care is limited by the lack of solid evidence about the associations of BMF with health outcomes apart from Parkinson's disease and colorectal cancer. We examined the prospective associations of BMF with major vascular and nonvascular diseases outside the digestive system.

Design: Population-based prospective cohort study.

Setting: The China Kadoorie Biobank in which participants from 10 geographically diverse areas across China were enrolled between 2004 and 2008.

Participants: 487 198 participants aged 30-79 years without cancer, heart disease or stroke at baseline were included and followed up for a median of 10 years. The usual BMF was self-reported once at baseline.

Primary and secondary outcome measures: Incident events of predefined major vascular and nonvascular diseases.

Results: In multivariable-adjusted analyses, participants having bowel movements "more than once a day" had higher risks of ischaemic heart disease (IHD), heart failure, chronic obstructive pulmonary disease, type 2 diabetes mellitus, and chronic kidney disease (CKD) when compared with the reference group ("once a day"). The respective hazard ratios (HRs) (95% confidence intervals [CIs]) were 1.12 (1.09 to 1.16), 1.33 (1.22 to 1.46), 1.28 (1.22 to 1.36), 1.20 (1.15 to 1.26), and 1.15 (1.07, to 1.24). The lowest BMF ("less than 3 times a week") was also associated with higher

For peer review only - http://bmjopen.bmj.com/site/about/guidelines.xhtml

risks of IHD, major coronary events, ischaemic stroke, and CKD. The respective HRs were 1.07 (1.02 to 1.12), 1.22 (1.10 to 1.36), 1.11 (1.05 to 1.16), and 1.20 (1.07 to 1.35).

Conclusion: BMF was associated with future risks of multiple vascular and nonvascular diseases. The integration of BMF assessment and health counselling into primary care should be considered.

Strengths and limitations of this study:

- In this large prospective cohort study of Chinese adults, bowel movement frequency (BMF) was associated with risks of multiple vascular and nonvascular diseases outside the digestive system.
- The results highlight that gastrointestinal health is related to the health of multiple systems and the integration of BMF assessment and health counselling into primary care practice should be considered.
- The prospective cohort design, large sample size, and long-term follow-up allowed the present study to produce reliable risk estimates.
- BMF was self-reported once at baseline and lack of other detailed information about intestinal health.
- Residual confounding may still exist, such as total energy intake, laxative use, and other non-cardiovascular medications that might directly affect BMF.

For peer review only

INTRODUCTION

A properly functioning gastrointestinal tract plays an essential role in health. Diseases of the digestion system have been prospectively associated with cardiovascular diseases (CVDs)[1-3], type 2 diabetes mellitus (T2DM)[4], and chronic kidney disease (CKD)[5]. Recent advancements in gut microbiota have also improved our understanding of the potential relationship of their composition, diversity, and metabolites with diseases.[6-10] Bowel movement is the endpoint of the digestion process. Bowel movement frequency (BMF) may serve as a simple indicator of adequate colonic function[11] and may reflect lifestyle factors (e.g., diet and exercise)[12 13] and individual characteristics (e.g., gut microbiota)[14 15]. It could inform the prevention and early detection of diseases.

A meta-analysis including four cohort and five case-control studies has reported that constipation is associated with subsequent diagnosis of Parkinson's disease.[16] Our recent study has shown that higher BMF is associated with an increased risk of colorectal cancer (CRC) only in the first five years of follow-up, indicating that abnormal increase of BMF may be an early symptom of CRC.[17] To our knowledge, only five cohort studies conducted in the United States and Japan have examined the associations between BMF and CVDs, but findings remained inconclusive.[18-22] For other outcomes, only one retrospective cohort study conducted in the United States has shown that constipation was associated with higher risks of incident CKD and end-stage renal disease.[23] More evidence from large population-based cohort studies

For peer review only - http://bmjopen.bmj.com/site/about/guidelines.xhtml

is warranted before such a message that abnormal BMF may indicate future disease risks could be applied to primary care practice.

Therefore, we prospectively examined the associations of BMF with major vascular and nonvascular diseases outside the digestive system in the China Kadoorie Biobank (CKB) study of 0.5 million adults.

SUBJECTS AND METHODS

Study population

CKB is an ongoing large prospective cohort study of over 0.5 million adults from 10 geographically diverse sites across China. The baseline survey was conducted during 2004-2008 and the follow-up was initiated soon afterward. Details of the study have been described previously.[24 25] Briefly, a total of 512 715 adults aged 30-79 years had valid baseline data including a complete interviewer-administered laptop-based questionnaire, physical measurements, and a written informed consent form. This study was approved by the Ethical Review Committee of the Chinese Centre for Disease Control and Prevention (Beijing, China) and the Oxford Tropical Research Ethics Committee, University of Oxford (UK).

In the main analyses, we excluded participants: (i) who had a previous diagnosis of cancer (n=2578), heart disease (n=15 472) or stroke (n=8884) at baseline; (ii) who were lost to follow-up soon after baseline (n=1); and (iii) who had missing body mass index (BMI; n=2). After these exclusions, 487 198 eligible participants remained. In

For peer review only - http://bmjopen.bmj.com/site/about/guidelines.xhtml

the analyses of specific diseases, we additionally excluded participants: (i) who had a prior diagnosis of emphysema, bronchitis or pulmonary heart disease (n=13 288), and who had airflow obstruction (AFO)[26] diagnosed by spirometry at baseline (n=23 767) (for chronic obstructive pulmonary disease [COPD]); (ii) who had a self-reported (n=16 162) or screen-detected (n=14 138) diabetes at baseline (for T2DM); and (iii) who had a prior diagnosis of kidney disease (n=7575) (for CKD).

Assessment of exposure

BMF was assessed at the baseline survey by the question “About how often do you have bowel movements each week?” The four response options were more than once a day, once a day, once every 2-3 days, or less than 3 times a week. After completion of the baseline survey in July 2008, we randomly selected about 5% of the surviving participants for a resurvey in the same year. To test the reproducibility of the BMF question, we included 1300 participants who completed the same questionnaire twice at an interval of fewer than 1.5 years (median 1.4 years). The linearly and quadratically weighted kappa coefficients[27] between the two questions were 0.40 and 0.46, respectively.

Assessment of covariates

The baseline questionnaire collected covariates information on sociodemographic characteristics, lifestyle behaviours, dietary habits, personal health, and family medical history. The daily level of physical activity was calculated by multiplying the metabolic equivalent tasks (METs) value for a particular type of physical activity by

For peer review only - http://bmjopen.bmj.com/site/about/guidelines.xhtml

hours spent on that activity per day and summing the MET-hours for all activities.[28] A short validated qualitative food frequency questionnaire with five categories of frequency (daily, 4 to 6 days per week, 1 to 3 days per week, monthly, or never or rarely) was used to collect information on 12 major food groups during the previous 12 months (see details in the **Supplementary File**).[29] The amounts of consumption were not collected.

Height, body weight, and waist circumference (WC) were measured with calibrated instruments. BMI was calculated as the measured weight in kilograms divided by the square of the measured height in metres. Prevalent diabetes at baseline was defined as self-reported diabetes diagnosed by a physician before the baseline survey or screen-detected diabetes, which was defined as[30] (i) random blood glucose ≥7.0 mmol/L and fasting time ≥8h, (ii) random blood glucose ≥11.1 mmol/L and fasting time <8h, or (iii) fasting blood glucose ≥7.0 mmol/L on subsequent testing. Forced expiratory volume in 1 second (FEV1) and forced vital capacity (FVC) were measured using a hand-held Micro (MS01) Spirometer (CareFusion Corp). Screen-detected AFO was defined as pre-bronchodilator FEV1/FVC <0.7.[26]

Assessment of outcomes

All participants were followed-up for incident disease outcomes since their enrolment into the study at baseline. Incident diseases were identified by using linkages with local disease and death registries and national health insurance (HI) system, and by active follow-up.[25] Until the end of 2016, nearly 97% participants had been linked to

For peer review only - http://bmjopen.bmj.com/site/about/guidelines.xhtml

the HI databases, with similar proportions of successful linkage for participants across the 10 survey sites. Trained staff blinded to baseline information coded all events using International Classification of Diseases, 10th Revision (ICD-10).

The study outcomes in the present analyses included a range of prespecified vascular and nonvascular diseases: (i) ischaemic heart disease (IHD) (I20-I25); (ii) major coronary events (MCEs) (including non-fatal I21-I23 and fatal I21-I25); (iii) heart failure (I50); (iv) haemorrhagic stroke (I61); (v) ischaemic stroke (I63); (vi) COPD (J41-J44); (vii) T2DM (E11, E14); (viii) CKD: including diabetes mellitus with renal complications (E10.2, E11.2, E12.2, E13.2, and E14.2), hypertensive renal disease (I12 and I13), glomerular disease (N03, N04, N05, and N07), renal tubulointerstitial disease (N11, N12, N13, N14, and N15), and renal failure (N18 and N19).

Statistical analysis

The mean and prevalence values of baseline characteristics were calculated across different BMF categories by using analyses of covariance for continuous variables and logistic regressions for categorical variables, adjusting for age (years), sex, and 10 survey sites where appropriate. Person-years at risk were calculated from the baseline date to the diagnosis of the study outcome of interest, death, loss to follow-up, or 31 December 2016, whichever came first.

Stratified Cox proportional hazards models were used to estimate adjusted hazard ratios (HRs) and 95% confidence intervals (CIs), with age as the underlying

For peer review only - http://bmjopen.bmj.com/site/about/guidelines.xhtml

time scale and stratified jointly by 10 survey sites and age at baseline (5-year intervals). The proportional hazards assumption for the Cox models was checked by log-log plots; no apparent violation was found. The covariates adjusted in the multivariable models were determined according to previous literatures and biological relevance.[12 13 18-23] The covariates included sex (for the whole cohort analysis); education; occupation; household income; marital status; family history of certain diseases (for corresponding disease analysis only); smoking status; total physical activity level; alcohol consumption; intake frequency of fresh vegetables, fresh fruits, and red meat; BMI; waist circumference; and prevalent hypertension and diabetes at baseline (no adjustment for T2DM). The linear trend test of disease risks across various metrics of BMF was performed by modelling the levels of ordered categorical variables as a continuous variable in a separate model.

In sensitivity analyses, we excluded cases that occurred during the first two years of follow-up. We also further adjusted for the following factors one by one as potential confounders: intake frequency of rice, wheat, other staple food, spicy food, and tea; consumption of nutritional supplements (fish oil/cod liver oil, vitamins, calcium/iron/zinc, ginseng and other herbal products); medications for CVDs (aspirin, ACE-I, beta-blocker, statins, diuretics and Ca^{2+} antagonist); medical history of kidney disease, peptic ulcer, cirrhosis or chronic hepatitis, and gallstone or gallbladder disease at baseline.

To explore whether the associations of BMF with the study outcomes differed by

For peer review only - http://bmjopen.bmj.com/site/about/guidelines.xhtml

baseline characteristics, we stratified the analyses by the following factors: sex (men or women); age (<60 yr, ≥60 yr); hypertension (presence or absence); obesity status defined jointly by BMI and WC (presence: BMI ≥28.0 kg/m^2 or WC ≥90.0cm (men)/ ≥85.0cm (women)[31]; absence). The definition of obesity status was used because of the significance of abdominal obesity for the rapid growth of disease risk in Chinese adults.[32] The tests for multiplicative interaction were performed using likelihood ratio tests by comparing models with and without cross-product terms between the stratifying variable and BMF categories.

P-values are presented as unadjusted for multiple testing unless otherwise indicated. For testing of multiple primary outcomes, a Bonferroni-correction was applied to the significance level, α = 0.05 (two-sided), that divided 0.05 by the number of outcomes examined (5 vascular and 3 nonvascular outcomes). The analyses were conducted using Stata (version 15.0, StataCorp）.

Patient and Public Involvement

Patients were not involved in the present study. The results of the main study were presented to study participants at the website of the CKB study (http://www.ckbiobank.org/site/) and by newsletters annually.

RESULTS

Baseline characteristics of study participants

The distributions of sociodemographic factors, lifestyle factors, and health status at

For peer review only - http://bmjopen.bmj.com/site/about/guidelines.xhtml

baseline across four categories of BMF are displayed in **Table 1**. Of the 487 198 participants analysed, the mean age was 51.5 years, 59.1% were women, and 43.1% were from urban areas. Participants who defecated more frequently were more likely to be men and rural residents. They were also more likely to have higher BMI and WC, and to have hypertension. Men who drank alcohol regularly were more likely to have frequent bowel movements.

Associations of BMF with risks of multiple vascular and nonvascular diseases

During a median follow-up of 10.1 years (interquartile range 2.0 years), we documented 40 347 IHD, 7972 MCEs, 8943 haemorrhagic strokes, 37 579 ischaemic strokes, 4204 heart failure, 11 054 COPD, 15 281 T2DM, and 6526 CKD. In the whole cohort, after multivariable adjustment, participants who reported having bowel movements "more than once a day" were associated with higher risks of IHD, heart failure, COPD, T2DM, and CKD when compared with the reference group ("once a day"). The corresponding HRs (95% CIs) were 1.12 (1.09 to 1.16), 1.33 (1.22 to 1.46), 1.28 (1.22 to 1.36), 1.20 (1.15 to 1.26), and 1.15 (1.07 to 1.24), respectively (all P values <0.00625; **Table 2**). At the same time, the lowest BMF ("less than 3 times a week") was associated with increased risks of IHD, MCEs, ischaemic stroke, and CKD. The corresponding HRs (95% CIs) were 1.07 (1.02 to 1.12), 1.22 (1.10 to 1.36), 1.11 (1.05 to 1.16), and 1.20 (1.07 to 1.35), respectively (all P values < 0.00625). We observed linear trends between BMF and the risks of certain outcomes,

For peer review only - http://bmjopen.bmj.com/site/about/guidelines.xhtml

with positive associations for heart failure, COPD, and T2DM, and negative associations for MCEs and ischaemic stroke (all *P* values for trend <0.00625) (**Table 2**). Results of stepwise adjusted models for each disease outcome are presented in **Supplementary Table 1**. The association between BMF and T2DM attenuated after additional adjustment for BMI and waist circumference.

Sensitive analyses

In the sensitivity analyses, the associations of BMF with multiple disease outcomes did not change appreciably after excluding cases that occurred in the first two years of follow-up. The HRs (95% CIs) were almost unchanged after additional adjustment for other potential confounders (data not shown).

Subgroup analyses

There was a significant interaction between BMF and obesity status on the risk of MCEs (*P* for interaction = 0.001). Compared to participants with BMF of once every 1-3 days, the increased risk of MCEs associated with lower BMF was only observed among obese participants (**Table 3**). There was no heterogeneity by sex in the association between BMF and most disease outcomes except for ischaemic stroke (*P* for interaction = 0.006) (**Supplementary Table 2**). The associations between BMF and all study outcomes were consistent across different age groups or hypertension status. (**Supplementary Table 3** and **Supplementary Table 4**).

For peer review only

DISCUSSION

In this large prospective cohort study of Chinese adults, BMF was associated with risks of multiple vascular and nonvascular diseases outside the digestive system independent of traditional lifestyle and intermediate risk factors for common non-communicable diseases. Compared with BMF of once a day, low BMF (<3 times/wk) was associated with increased risks of MCEs and ischaemic stroke; more frequent bowel movements were associated with increased risks of heart failure, COPD, and T2DM. Increased risks of IHD and CKD were observed in participants who were either constipated or had frequent bowel movements. BMF was not associated with the risk of haemorrhagic stroke. Our findings persisted after excluding cases that occurred in the first two years of follow-up.

Several prospective studies have examined the associations of BMF with CVDs.[18-21] The Women's Health Initiative observational study (WHI) followed 73 047 post-menopausal women for 6.4 years and assessed the severity of constipation by self-report at baseline.[18] In that study, women with severe constipation had a 23% higher risk of death from a composite of cardiovascular events (HR=1.23; 95% CI: 1.03-1.47) compared with women with no constipation. Separate analysis for each type of cardiovascular events was limited by the small number of events. Another retrospective cohort study conducted in over three million US veterans (93.2% of men) showed similar results.[22] In that study, constipation was defined as either having ≥2 prescriptions for ≥30-day supply of laxatives each or having ≥2 diagnoses for

For peer review only - http://bmjopen.bmj.com/site/about/guidelines.xhtml

constipation identified by ICD-9-CM. Compared with participants without constipation, those with constipation had higher risks of incident coronary heart disease (HR=1.11; 95% CI: 1.08-1.14) and ischaemic stroke (HR=1.19; 95% CI:1.15-1.22). However, findings from the Nurse's Health Study (NHS) of 86 289 women (aged 30-55 years, followed-up ~30 years) showed no associations of BMF with incident coronary heart disease, incident stroke, and cardiovascular mortality.[21] Compared with women with daily bowel movements, those with BMF >1 time/day had a modest increase in the risk of total mortality (HR=1.10; 95% CI: 1.06-1.15).

The Ohsaki cohort including 45 112 Japanese aged 40-79 years (follow-up 13.3 years) showed that the risk of CVD mortality increased in participants reporting BMF of 1 time/2-3 days (HR=1.21; 95%: 1.08-1.35) and ≤1 time/4 days (HR=1.39; 95% CI: 1.06-1.81) compared with those reporting ≥1 time/day.[19] The respective HRs (95% CI) for death from ischaemic stroke were 1.27 (1.00-1.61) and 1.97 (1.21-3.21). The study was underpowered for analyses of IHD and stroke mortality of other subtypes. Another similar study based on the Japan Collaborative Cohort (JACC) of 72 014 Japanese aged 40-79 years did not observe significant associations of BMF with deaths from coronary heart disease, ischaemic and haemorrhagic stroke, but it was also underpowered to allow a firm conclusion.[20] However, this study indicated that laxative use, a potential reflection of severe constipation, was associated with increased risks of deaths from coronary heart disease and ischaemic stroke.

In the present study, we showed that low BMF was associated with increased

For peer review only - http://bmjopen.bmj.com/site/about/guidelines.xhtml

risks of MCEs and ischaemic stroke, in line with findings from most previous studies. With regard to the associations between BMF and other outcomes, only one retrospective cohort study conducted in US veterans examined the association between constipation and incident CKD.[23] In that study, veterans with constipation had higher risks of CKD (HR=1.13; 95% CI: 1.11-1.14) and end-stage renal disease (HR=1.09; 95% CI: 1.01-1.18) compared with those without constipation.

Previous studies have linked constipation or high frequency of bowel movements to the alterations of composition, diversity, and functions of gut microbiota.[15 33 34] Altered gut microbiota has been suggested to interact with the host through both metabolism dependent and independent processes and contribute to the development and progression of diseases such as atherosclerosis, heart failure, CKD, and T2DM.[35] Similarly, the gastrointestinal tract and respiratory tract, albeit separate organs, are part of a shared mucosal immune system (i.e. the gut-lung axis).[36] The microbiota and metabolites in the gut and the lungs can modulate systemic and local immunity and influence the pathogenesis of respiratory diseases.[36] Also, slow transit constipation is accompanied by reduced fermentation efficacy of complex carbohydrates, which diminishes the production of short-chain fatty acids, an energy source for colonocytes, leading to oxidative stress.[37] Oxidative stress has also been associated with the onset and progression of atherosclerosis, CVDs, neurological diseases, and kidney diseases.[38] Although several plausible mechanisms underlying these associations have been suggested, further research is warranted to provide convincing evidence of the

For peer review only

For peer review only - http://bmjopen.bmj.com/site/about/guidelines.xhtml

causal connection.

Dietary factors might influence BMF. Previous studies in CKB have shown that consumption of fresh fruits, fresh eggs, and spicy food was each associated with lower risks of CVDs[39-41] and diabetes[42]. In the present study, the relative risk estimates for multiple disease outcomes did not change appreciably after adjusting for these dietary factors, indicating that the associations between BMF and multiple diseases might be independent of dietary factors. Other established risk factors for chronic diseases that may affect BMF, such as physical inactivity and obesity[12 13], did not confound these associations either, except for obesity in the analysis of T2DM. Besides, most of the associations between BMF and multiple disease outcomes were consistent across baseline subgroups defined by sex, age, obesity status, and hypertension status in the present analyses. The significant increase in the risk of MCEs associated with constipation was only observed among obese participants. We assume that both obesity- and constipation-related oxidative stress act synergistically to increase the risk of MCEs. There is still a limited understanding of the sex difference in the association between constipation and ischaemic stroke.

To our knowledge, this is the first prospective study examining the associations of BMF with a range of disease outcomes outside the digestive system collected through linkages to multiple data systems. The present study had a large sample size and long-term follow-up, and hence was well powered for analyses of CVD subtypes and other outcomes. Participants included both men and women geographically

For peer review only - http://bmjopen.bmj.com/site/about/guidelines.xhtml

spread across urban and rural areas across China. Prospective design and long-term follow-up minimized the potential for reverse causation. A detailed collection of lifestyle factors and other covariates allowed adjustment for several potential confounders.

However, there are several limitations. First, BMF was self-reported, which may cause nondifferential misclassification and potentially attenuate the true associations. BMF and covariate information collected once at baseline might have changed during the follow-up. Second, although we controlled for several potential confounders, residual confounding may still exist. For example, we were not able to adjust for total energy intake, laxative use, opioids use, and other non-cardiovascular medications that might directly affect BMF. Third, due to the lack of detailed information about intestinal health (e.g. colonic transit time, stool consistency, gut microbiota, and blood biomarkers), there is a limited understanding of the relationship between the gut and multiple diseases.

In conclusion, we observed that BMF was associated with future risk of multiple vascular and nonvascular diseases, including CVD, COPD, T2DM, and CKD in this Chinese population. Our findings highlight that gastrointestinal health is related to the health of multiple systems and should stimulate future interest in elucidating the mechanisms underlying these associations. BMF is an easily assessed indicator and has been correlated with colonic transit time, an indicator of gastrointestinal function that is more accurate but not easy for use.[11] Based on the findings of the current and

For peer review only

For peer review only - http://bmjopen.bmj.com/site/about/guidelines.xhtml

previous studies,[16 17] people with abnormal BMF should consider the possibility of undiagnosed diseases and be aware of the potential future risks of various health conditions. The integration of BMF assessment and health counselling into primary care practice should be considered. Further studies are needed to evaluate factors that may influence BMF to improve and maintain digestive health.

Acknowledgements: The most important acknowledgment is to the participants in the study and the members of the survey teams in each of the 10 regional centres, as well as to the project development and management teams based at Beijing, Oxford and the 10 regional centres. The members of the steering committee and collaborative group are listed in the supplementary file. We thank Dr. Yuanjie Pang for her help in revising this article.

Contributors: JL and LL conceived and designed the paper. LL, ZC, and JC, as members of the CKB steering committee, designed and supervised the conduct of the whole study, obtained funding, and together with YG, ZB, LY, HD, YC, SYan, and YZ acquired the data. SYang, MF, CY, and JL analysed the data. SYang and JL drafted the manuscript. CY, YG, ZB, MF, LY, HD, YC, SYan, YZ, JC, ZC, JL, and LL contributed to the interpretation of the results and critical revision of the manuscript for important intellectual content. All authors contributed to and approved the final manuscript. JL is the study guarantor.

Funding: This work was supported by grants (2016YFC0900500, 2016YFC0900501, 2016YFC0900504) from the National Key R&D Program of China. The CKB baseline survey and the first re-survey were supported by a grant from the Kadoorie Charitable Foundation in Hong Kong. The long-term follow-up is supported by grants from the UK Wellcome Trust (202922/Z/16/Z, 088158/Z/09/Z, 104085/Z/14/Z), National Natural Science Foundation of China (81390540, 81390541, 81390544), and Chinese Ministry of Science and Technology (2011BAI09B01). The funders had no role in the study design, data collection, data analysis and interpretation, writing of the report, or the decision to submit the article for publication.

Competing interests: None declared.

For peer review only - http://bmjopen.bmj.com/site/about/guidelines.xhtml

For peer review only

Patient consent: Obtained.

Ethics approval: The Ethical Review Committee of the Chinese Center for Disease Control and Prevention (Beijing, China) (Ethics ID number of approval: 005/2004) and the Oxford Tropical Research Ethics Committee, University of Oxford (UK) (Ethics ID number of approval: OXTREC 025-04).

Data availability statement: The dataset supporting the conclusions of this article is available from the study website (http://www.ckbiobank.org), along with the access policy and procedures.

For peer review only

For peer review only - http://bmjopen.bmj.com/site/about/guidelines.xhtml

REFERENCES

1. Lee SB, Park G-M, Lee J-Y, et al. Association between non-alcoholic fatty liver disease and subclinical coronary atherosclerosis: An observational cohort study. *J Hepatol* 2018;68(5):1018-24. doi:10.1016/j.jhep.2017.12.012

2. Keller JJ, Wang J, Huang Y-L, et al. Increased risk of stroke among patients with ulcerative colitis: a population-based matched cohort study. *Int J Colorectal Dis* 2014;29(7):805-12. doi:10.1007/s00384-014-1862-6

3. Fan LL, Chen BH, Dai ZJ. The relation between gallstone disease and cardiovascular disease. *Sci Rep* 2017;7(1):15104. doi:10.1038/s41598-017-15430-5

4. Lv J, Yu C, Guo Y, et al. Gallstone Disease and the Risk of Type 2 Diabetes. *Sci Rep* 2017;7(1):15853. doi:10.1038/s41598-017-14801-2

5. Sinn DH, Kang D, Jang HR, et al. Development of chronic kidney disease in patients with non-alcoholic fatty liver disease: A cohort study. *J Hepatol* 2017;67(6):1274-80. doi:10.1016/j.jhep.2017.08.024

6. Lambeth SM, Carson T, Lowe J, et al. Composition, Diversity and Abundance of Gut Microbiome in Prediabetes and Type 2 Diabetes. *J Diabetes Obes* 2015;2(3):1-7. doi:10.15436/2376-0949.15.031

7. Yamashiro K, Tanaka R, Urabe T, et al. Gut dysbiosis is associated with metabolism and systemic inflammation in patients with ischemic stroke. *PLoS One* 2017;12(2):e0171521. doi:10.1371/journal.pone.0171521

8. Heianza Y, Ma W, Manson JE, et al. Gut Microbiota Metabolites and Risk of Major Adverse Cardiovascular Disease Events and Death: A Systematic Review and Meta-Analysis of Prospective Studies. *J Am Heart Assoc* 2017;6(7):e004947. doi:10.1161/JAHA.116.004947

9. Xu KY, Xia GH, Lu JQ, et al. Impaired renal function and dysbiosis of gut microbiota contribute to increased trimethylamine-N-oxide in chronic kidney disease patients. *Sci Rep* 2017;7(1):1445. doi:10.1038/s41598-017-01387-y

10. Pasini E, Aquilani R, Testa C, et al. Pathogenic Gut Flora in Patients With Chronic Heart Failure. *JACC Heart Fail* 2016;4(3):220-7. doi:10.1016/j.jchf.2015.10.009

11. Tornblom H, Van Oudenhove L, Sadik R, et al. Colonic Transit Time and IBS Symptoms: What's the Link. *Am J Gastroenterol* 2012;107(5):754-60. doi:10.1038/ajg.2012.5

12. Sanjoaquin MA, Appleby PN, Spencer EA, et al. Nutrition and lifestyle in relation to bowel movement frequency: a cross-sectional study of 20,630 men and women in EPIC-Oxford. *Public Health Nutr* 2004;7(1):77-83. doi:10.1079/Phn2003522

13. Nakaji S, Tokunaga S, Sakamoto J, et al. Relationship between lifestyle factors and defecation in a Japanese population. *Eur J Nutr* 2002;41(6):244-8. doi:10.1007/s00394-002-0380-4

14. Hadizadeh F, Walter S, Belheouane M, et al. Stool frequency is associated with gut microbiota composition. *Gut* 2017;66(3):559-60. doi:10.1136/gutjnl-2016-311935

15. Oki K, Toyama M, Banno T, et al. Comprehensive analysis of the fecal microbiota of healthy Japanese adults reveals a new bacterial lineage associated with a phenotype characterized by a high frequency of bowel movements and a lean body type. *BMC Microbiol* 2016;16(1):284. doi:10.1186/s12866-016-0898-x

16. Adams-Carr KL, Bestwick JP, Shribman S, et al. Constipation preceding Parkinson's disease: a systematic review and meta-analysis. *J Neurol Neurosurg Psychiatry* 2016;87(7):710-6. doi:10.1136/jnnp-2015-311680

17. Yang S, Shen Z, Yu C, et al. Association between the frequency of bowel movements and the risk of colorectal cancer in Chinese adults. *Chin J Epidemiol* 2019;40(4):382-8. doi:10.3760/cma.j.issn.0254-6450.2019.04.003

18. Salmoirago-Blotcher E, Crawford S, Jackson E, et al. Constipation and risk of cardiovascular disease among

For peer review only - http://bmjopen.bmj.com/site/about/guidelines.xhtml

postmenopausal women. *Am J Med* 2011;124(8):714-23. doi:10.1016/j.amjmed.2011.03.026
19. Honkura K, Tomata Y, Sugiyama K, et al. Defecation frequency and cardiovascular disease mortality in Japan: The Ohsaki cohort study. *Atherosclerosis* 2016;246:251-6. doi:10.1016/j.atherosclerosis.2016.01.007
20. Kubota Y, Iso H, Tamakoshi A, et al. Bowel Movement Frequency, Laxative Use, and Mortality From Coronary Heart Disease and Stroke Among Japanese Men and Women: The Japan Collaborative Cohort (JACC) Study. *J Epidemiol* 2016;26(5):242-8. doi:10.2188/jea.JE20150123
21. Ma WJ, Li YP, Heianza Y, et al. Associations of Bowel Movement Frequency with Risk of Cardiovascular Disease and Mortality among US Women. *Sci Rep* 2016;6:33005. doi:10.1038/srep33005
22. Sumida K, Molnar MZ, Potukuchi PK, et al. Constipation and risk of death and cardiovascular events. *Atherosclerosis* 2019;281:114-20. doi:10.1016/j.atherosclerosis.2018.12.021
23. Sumida K, Molnar MZ, Potukuchi PK, et al. Constipation and Incident CKD. *J Am Soc Nephrol* 2017;28(4):1248-58. doi:10.1681/ASN.2016060656
24. Chen ZM, Lee L, Chen JS, et al. Cohort profile: The Kadoorie study of chronic disease in China (KSCDC). *Int J Epidemiol* 2005;34(6):1243-9. doi:10.1093/ije/dyi1d74
25. Chen ZM, Chen JS, Collins R, et al. China Kadoorie Biobank of 0.5 million people: survey methods, baseline characteristics and long-term follow-up. *Int J Epidemiol* 2011;40(6):1652-66. doi:10.1093/ije/dyr120
26. Kurmi OP, Li LM, Wang J, et al. COPD and its association with smoking in the Mainland China: a cross-sectional analysis of 0.5 million men and women from ten diverse areas. *Int J Chron Obstruct Pulmon Dis* 2015;10:655-65. doi:10.2147/Copd.S75454
27. Li P. A Note on the Linearly and Quadratically Weighted Kappa Coefficients. *Psychometrika* 2016;81(3):795-801. doi:10.1007/s11336-016-9501-5
28. Du HD, Li LM, Whitlock G, et al. Patterns and socio-demographic correlates of domain-specific physical activities and their associations with adiposity in the China Kadoorie Biobank study. *BMC Public Health* 2014;14:826. doi:10.1186/1471-2458-14-826
29. Shen Q, Yu C, Guo Y, et al. Habitual Tea Consumption and Risk of Fracture in 0.5 Million Chinese Adults: A Prospective Cohort Study. *Nutrients* 2018;10(11):E1633. doi:10.3390/nu10111633
30. Bragg F, Holmes MV, Iona A, et al. Association Between Diabetes and Cause-Specific Mortality in Rural and Urban Areas of China. *J Am Med Assoc* 2017;317(3):280-89. doi:10.1001/jama.2016.19720
31. Division of Disease Control. Ministry of Health of the People's Republic of China. Guideline for prevention and control of overweight and obesity in Chinese adults. Beijing: People's Medical Publishing House 2006.
32. Xi B, Liang Y, He T, et al. Secular trends in the prevalence of general and abdominal obesity among Chinese adults, 1993-2009. *Obes Rev* 2012;13(3):287-96. doi:10.1111/j.1467-789X.2011.00944.x
33. Zhuang X, Tian Z, Li L, et al. Fecal Microbiota Alterations Associated With Diarrhea-Predominant Irritable Bowel Syndrome. *Front Microbiol* 2018;9:1600. doi:10.3389/fmicb.2018.01600
34. Mancabelli L, Milani C, Lugli GA, et al. Unveiling the gut microbiota composition and functionality associated with constipation through metagenomic analyses. *Sci Rep* 2017;7(1):9879. doi:10.1038/s41598-017-10663-w
35. Tang WHW, Kitai T, Hazen SL. Gut Microbiota in Cardiovascular Health and Disease. *Circ Res* 2017;120(7):1183-96. doi:10.1161/Circresaha.117.309715
36. Budden KF, Gellatly SL, Wood DLA, et al. Emerging pathogenic links between microbiota and the gut-lung axis. *Nat Rev Microbiol* 2017;15(1):55-63. doi:10.1038/nrmicro.2016.142
37. Vermorken AJ, Andres E, Cui Y. Bowel movement frequency, oxidative stress and disease prevention. *Mol*

For peer review only - http://bmjopen.bmj.com/site/about/guidelines.xhtml

Clin Oncol 2016;5(4):339-42. doi:10.3892/mco.2016.987

38. Pizzino G, Irrera N, Cucinotta M, et al. Oxidative Stress: Harms and Benefits for Human Health. *Oxid Med Cell Longev* 2017;2017:8416763. doi:10.1155/2017/8416763

39. Lv J, Qi L, Yu C, et al. Consumption of spicy foods and total and cause specific mortality: population based cohort study. *BMJ* 2015;351:h3942. doi:10.1136/bmj.h3942

40. Qin C, Lv J, Guo Y, et al. Associations of egg consumption with cardiovascular disease in a cohort study of 0.5 million Chinese adults. *Heart* 2018;104(21):1756-63. doi:10.1136/heartjnl-2017-312651

41. Du H, Li L, Bennett D, et al. Fresh Fruit Consumption and Major Cardiovascular Disease in China. *N Engl J Med* 2016;374(14):1332-43. doi:10.1056/NEJMoa1501451

42. Du H, Li L, Bennett D, et al. Fresh fruit consumption in relation to incident diabetes and diabetic vascular complications: A 7-y prospective study of 0.5 million Chinese adults. *PLoS Med* 2017;14(4):e1002279. doi:10.1371/journal.pmed.1002279

For peer review only

For peer review only - http://bmjopen.bmj.com/site/about/guidelines.xhtml

Table 1 | Baseline characteristics according to BMF at baseline for 487 198 participants

	More than once a day	Once a day	Once every 2-3 days	Less than 3 times a week
**Number of participants, n (%)**	46 426 (9.5)	373 054 (76.6)	46 570 (9.6)	21 148 (4.3)
**Women, %**	45.9	57.7	72.6	82.5
**Age, year**	53.0	51.5	50.6	51.0
**Urban area, %**	33.3	43.9	43.7	49.4
**Middle school and above, %**	46.7	49.5	49.1	48.4
**Agricultural and industrial workers, %**	58.2	57.4	57.9	57.5
**Household income ≥20 000 RMB/yr, %**	41.8	43.4	40.6	38.8
**Married, %**	91.4	91.0	90.2	89.2
**Current tobacco smoker, %**				
Men	68.2	67.5	70.4	72.5
Women	2.6	2.7	2.8	3.2
**Current daily alcohol drinker, %**				
Men	25.4	21.0	15.3	14.8
Women	1.0	1.0	0.8	0.7
**Physical activity, MET-h/d**	22.0	21.6	21.3	20.9
**Regular consumption of*, %**				
Red meat	47.0	47.4	46.9	45.3
Fresh vegetables	98.5	98.2	98.5	98.2
Fresh fruits	27.2	28.1	26.7	23.5
**Body mass index, kg/m²**	24.5	23.6	22.8	22.6
**Waist circumference, cm**	82.4	80.2	77.9	77.3
**Hypertension, %**	38.8	34.2	27.5	25.7
**Diabetes, %**	6.0	5.2	5.5	6.4
**Kidney disease†, %**	1.9	1.4	1.3	1.4
**COPD†, %**	8.5	6.9	7.0	7.7
**Family history of, %**				
Cancer	18.0	16.4	16.6	17.2
Diabetes	8.3	6.7	6.7	6.8
Heart attack	3.6	3.1	3.1	3.6
Stroke	19.4	17.5	17.0	17.2

BMF, bowel movement frequency; MET, metabolic equivalent of task; COPD, chronic obstructive pulmonary disease.

Baseline characteristics were presented as mean or percentage. Age and sex were not adjusted, and others were adjusted for age, sex, and survey sites as appropriate. Linear trend was assessed by assigning consecutive integers to four BMF categories in a separate model. All *P* values for trend were <0.001, except for agricultural and industrial workers (*P*=0.359), regular consumption of fresh vegetables (*P*=0.955), the prevalence of diabetes (*P*=0.024), family history of cancer (*P*=0.010) and heart attack (*P*=0.486).

For peer review only - http://bmjopen.bmj.com/site/about/guidelines.xhtml

For peer review only

*Regular consumption was defined as consumption at least 4-6 days per week.

†Participants with kidney disease or COPD were further excluded from the analyses of the corresponding outcome.

For peer review only

For peer review only - http://bmjopen.bmj.com/site/about/guidelines.xhtml

Table 2 | HRs (95% CIs) for associations between BMF and multiple vascular and nonvascular diseases

Diseases	More than once a day	Once a day	Once every 2-3 days	Less than 3 times a week	*P* value for trend*
**Vascular diseases**					
**IHD**					
No. of cases	4668	30 470	3448	1761	
Cases/PYs (1/1000)	10.43	8.45	7.60	8.59	
Multivariable adjusted	1.12 (1.09-1.16)	1.00	1.00 (0.96-1.03)	1.07 (1.02-1.12)	0.008
**MCEs**					
No. of cases	871	5960	765	376	
Cases/PYs (1/1000)	1.88	1.61	1.64	1.78	
Multivariable adjusted	1.03 (0.96-1.11)	1.00	1.10 (1.02-1.19)	1.22 (1.10-1.36)	0.002
**Haemorrhagic stroke**					
No. of cases	1028	6856	723	336	
Cases/PYs (1/1000)	2.22	1.85	1.56	1.60	
Multivariable adjusted	0.97 (0.90-1.03)	1.00	0.98 (0.91-1.06)	1.07 (0.95-1.19)	0.256
**Ischaemic stroke**					
No. of cases	4044	28 594	3291	1650	
Cases/PYs (1/1000)	9.00	7.91	7.24	8.03	
Multivariable adjusted	1.01 (0.98-1.05)	1.00	1.04 (1.00-1.08)	1.10 (1.05-1.16)	0.002
**Heart failure**					
No. of cases	535	3068	398	203	
Cases/PYs (1/1000)	1.16	0.83	0.86	0.96	
Multivariable adjusted	1.33 (1.22-1.46)	1.00	0.96 (0.86-1.06)	0.99 (0.86-1.15)	<0.001
**Nonvascular diseases**					
**COPD**					
No. of cases	1518	8167	926	443	
Cases/PYs (1/1000)	3.63	2.37	2.14	2.29	
Multivariable adjusted	1.28 (1.22-1.36)	1.00	0.89 (0.83-0.95)	0.92 (0.84-1.02)	<0.001
**T2DM**					
No. of cases	2207	11 514	1077	483	
Cases/PYs (1/1000)	5.14	3.30	2.46	2.46	
Multivariable adjusted	1.20 (1.15-1.26)	1.00	0.86 (0.81-0.92)	0.84 (0.77-0.92)	<0.001
**CKD**					
No. of cases	821	4786	605	314	
Cases/PYs (1/1000)	1.81	1.31	1.33	1.52	
Multivariable adjusted	1.15 (1.07-1.24)	1.00	1.07 (0.98-1.17)	1.20 (1.07-1.35)	0.577

HR: hazard ratio; CI: confidence interval; BMF: bowel movement frequency; IHD: ischaemic heart disease; MCEs: major coronary events; COPD: chronic obstructive pulmonary disease; T2DM: type 2 diabetes mellitus; CKD: chronic kidney disease.

Cox regression models were stratified by 5-year groups and ten survey sites, with age as the underlying time scale.

Multivariable models were adjusted for sex (men or women); level of education (no formal school, primary school, middle school,

For peer review only - http://bmjopen.bmj.com/site/about/guidelines.xhtml

high school, college, or university or higher); occupation (agricultural, industrial, administrative or managerial, professional or technical, sales or service, retired, house wife or husband, self-employed, unemployed, other); household income (<2500, 2500-4999, 5000-9999, 10 000-19 999, 20 000-34 999, ≥35 000 RMB per year); marital status (married, widowed, divorced or separated, or never married); family history of certain diseases (presence, absence, or unclear; for corresponding disease only; no adjustment for heart failure, COPD, and CKD); smoking status (non-smoker, former smoker, current smoker who smoked <15, 15-24, or ≥25 cigarettes or equivalents per day; those who had stopped smoking because of ill health were included in current smokers to avoid bias); total physical activity level (MET-h/day); alcohol consumption (non-drinker, former weekly drinker, weekly drinker, daily drinker with an intake of <15, 15-29, 30-59, or ≥60 g/day of pure alcohol); intake frequency of fresh vegetables, fresh fruit, and red meat (daily, 4-6 days/week, 1-3 days/week, monthly, or rarely or never); BMI (kg/m2); waist circumference (cm); prevalent hypertension and diabetes at baseline (presence or absence, no adjustment for T2DM).

*Assessed by assigning consecutive integers to four BMF categories in a separate model. A Bonferroni-corrected threshold was used, α=0.00625.

For peer review only

Table 3 | HRs (95% CIs) for associations between BMF and multiple vascular and nonvascular diseases stratified by obesity status

Diseases	Obesity subgroups	More than once a day		Once every 1-3 days*		Less than 3 times a week		*P* value for interaction†
		Cases	*HR (95%CI)*	Cases	*HR (95%CI)*	Cases	*HR (95%CI)*	
**Vascular diseases**								
**IHD**								0.383
	No	2771	1.15 (1.10-1.19)	22 109	1.00	1258	1.04 (0.98-1.11)	
	Yes	1897	1.10 (1.04-1.15)	11 809	1.00	503	1.13 (1.03-1.23)	
**MCEs**								0.001
	No	577	1.07 (0.98-1.17)	4478	1.00	238	1.07 (0.93-1.22)	
	Yes	294	0.91 (0.81-1.03)	2247	1.00	138	1.57 (1.31-1.86)	
**Haemorrhagic stroke**								0.624
	No	706	0.98 (0.90-1.06)	5543	1.00	269	1.08 (0.96-1.23)	
	Yes	322	0.93 (0.82-1.04)	2036	1.00	67	1.05 (0.82-1.34)	
**Ischaemic stroke**								0.688
	No	2367	1.02 (0.98-1.07)	20 903	1.00	1221	1.10 (1.03-1.16)	
	Yes	1677	1.01 (0.96-1.06)	10 982	1.00	429	1.07 (0.97-1.18)	
**Heart failure**								0.356
	No	360	1.36 (1.22-1.52)	2493	1.00	152	0.96 (0.81-1.13)	
	Yes	175	1.25 (1.06-1.47)	973	1.00	51	1.22 (0.92-1.62)	
**Nonvascular diseases**								
**COPD**								0.353
	No	1132	1.29 (1.21-1.37)	7070	1.00	371	0.98 (0.88-1.09)	
	Yes	386	1.21 (1.08-1.35)	2023	1.00	72	0.90 (0.71-1.14)	
**T2DM**								0.506
	No	1048	1.35 (1.27-1.44)	7069	1.00	338	0.80 (0.72-0.90)	

For peer review only

For peer review only - http://bmjopen.bmj.com/site/about/guidelines.xhtml

Diseases	Obesity	More than once a day		Once every 1-3 days*		Less than 3 times a week		*P* value for
	Yes	1159	1.25 (1.17-1.33)	5522	1.00	142	0.77 (0.65-0.91)	
**CKD**								0.051
	No	545	1.22 (1.11-1.33)	3789	1.00	252	1.23 (1.08-1.40)	
	Yes	276	1.01 (0.88-1.15)	1602	1.00	62	1.05 (0.81-1.35)	

HR: hazard ratio; CI: confidence interval; BMF: bowel movement frequency; IHD: ischaemic heart disease; MCEs: major coronary events; COPD: chronic obstructive pulmonary disease; T2DM: type 2 diabetes mellitus; CKD: chronic kidney disease; BMI: body mass index; WC: waist circumference.

Multivariable models adjusted for the same set of covariates as Table 2, except for BMI (kg/m2) and waist circumference (cm). Obesity status was defined according to BMI and WC, with "yes" as BMI≥28.0 kg/m^2 or WC≥90.0 cm (men)/≥85.0 cm (women).

*Combined group including "once a day" and "once every 2-3 days".

†A Bonferroni-corrected threshold was used, α=0.00625.

For peer review only

Supplementary Information

Bowel movement frequency and risks of major vascular and nonvascular diseases: a population-based cohort study among Chinese adults

Yang, S. *et al*

Supplementary Tables **1**
Supplementary Table 1 | HRs (95% CIs) for associations between BMF and multiple vascular and nonvascular diseases with stepwise adjustment 1
Supplementary Table 2 | HRs (95% CIs) for associations between BMF and multiple vascular and nonvascular diseases stratified by sex 3
Supplementary Table 3 | HRs (95% CIs) for associations between BMF and multiple vascular and nonvascular diseases stratified by baseline age 5
Supplementary Table 4 | HRs (95% CIs) for associations between BMF and multiple vascular and nonvascular diseases stratified by baseline hypertension 7

Supplementary Note **9**
1. CKB collaborative group 9
2. Food frequency questionnaire 11

For peer review only

Supplementary Tables

Supplementary Table 1 | HRs (95% CIs) for associations between BMF and multiple vascular and nonvascular diseases with stepwise adjustment

Diseases	More than once a day	Once a day	Once every 2-3 days	Less than 3 times a week	*P* value for trend*
**Vascular diseases**					
**IHD**					
No. of participants	46 426	373 054	46 570	21 148	
No. of cases	4668	30 470	3448	1761	
Cases/PYs (1/1000)	10.43	8.45	7.60	8.59	
Model 1	1.16 (1.13-1.20)	1.00	0.96 (0.93-0.99)	1.03 (0.98-1.08)	<0.001
Model 2	1.16 (1.13-1.20)	1.00	0.96 (0.93-1.00)	1.03 (0.98-1.08)	<0.001
Model 3	1.17 (1.13-1.20)	1.00	0.95 (0.92-0.99)	1.01 (0.96-1.06)	<0.001
Model 4	1.12 (1.09-1.16)	1.00	1.00 (0.96-1.03)	1.07 (1.02-1.12)	0.008
**MCEs**					
No. of participants	46 426	373 054	46 570	21 148	
No. of cases	871	5960	765	376	
Cases/PYs (1/1000)	1.88	1.61	1.64	1.78	
Model 1	1.05 (0.98-1.13)	1.00	1.12 (1.04-1.21)	1.28 (1.16-1.43)	<0.001
Model 2	1.05 (0.98-1.13)	1.00	1.09 (1.01-1.18)	1.23 (1.10-1.36)	0.005
Model 3	1.05 (0.98-1.13)	1.00	1.08 (1.00-1.16)	1.19 (1.07-1.32)	0.023
Model 4	1.03 (0.96-1.11)	1.00	1.10 (1.02-1.19)	1.22 (1.10-1.36)	0.002
**Haemorrhagic stroke**					
No. of participants	46 426	373 054	46 570	21 148	
No. of cases	1028	6856	723	336	
Cases/PYs (1/1000)	2.22	1.85	1.56	1.60	
Model 1	1.02 (0.95-1.09)	1.00	0.95 (0.88-1.03)	1.03 (0.92-1.15)	0.576
Model 2	1.01 (0.94-1.08)	1.00	0.93 (0.86-1.01)	1.00 (0.89-1.11)	0.306
Model 3	0.99 (0.93-1.06)	1.00	0.93 (0.86-1.01)	0.98 (0.88-1.10)	0.340
Model 4	0.97 (0.90-1.03)	1.00	0.98 (0.91-1.06)	1.07 (0.95-1.19)	0.256
**Ischaemic stroke**					
No. of participants	46 426	373 054	46 570	21 148	
No. of cases	4044	28 594	3291	1650	
Cases/PYs (1/1000)	9.00	7.91	7.24	8.03	
Model 1	1.07 (1.04-1.11)	1.00	0.99 (0.96-1.03)	1.05 (1.00-1.10)	0.178
Model 2	1.07 (1.03-1.10)	1.00	0.99 (0.95-1.03)	1.04 (0.99-1.09)	0.135
Model 3	1.06 (1.03-1.10)	1.00	0.99 (0.95-1.02)	1.03 (0.98-1.08)	0.073
Model 4	1.01 (0.98-1.05)	1.00	1.04 (1.00-1.08)	1.10 (1.05-1.16)	0.002
**Heart failure**					
No. of participants	46 426	373 054	46 570	21 148	
No. of cases	535	3068	398	203	

For peer review only - http://bmjopen.bmj.com/site/about/guidelines.xhtml

Diseases	More than once a day	Once a day	Once every 2-3 days	Less than 3 times a week	*P* value for trend*
Cases/PYs (1/1000)	1.16	0.83	0.86	0.96	
Model 1	1.35 (1.23-1.48)	1.00	0.98 (0.88-1.09)	1.04 (0.90-1.21)	<0.001
Model 2	1.34 (1.23-1.48)	1.00	0.97 (0.87-1.08)	1.02 (0.88-1.17)	<0.001
Model 3	1.35 (1.23-1.48)	1.00	0.95 (0.85-1.05)	0.98 (0.85-1.13)	<0.001
Model 4	1.33 (1.22-1.46)	1.00	0.96 (0.86-1.06)	0.99 (0.86-1.15)	<0.001
**Nonvascular diseases**					
**COPD**					
No. of participants	42 122	347 771	43 350	19 414	
No. of cases	1518	8167	926	443	
Cases/PYs (1/1000)	3.63	2.37	2.14	2.29	
Model 1	1.26 (1.19-1.33)	1.00	0.93 (0.87-1.00)	1.00 (0.91-1.11)	<0.001
Model 2	1.25 (1.19-1.33)	1.00	0.92 (0.86-0.99)	0.97 (0.88-1.07)	<0.001
Model 3	1.26 (1.19-1.34)	1.00	0.91 (0.85-0.97)	0.94 (0.86-1.04)	<0.001
Model 4	1.28 (1.22-1.36)	1.00	0.89 (0.83-0.95)	0.92 (0.84-1.02)	<0.001
**T2DM**					
No. of participants	43 703	353 573	44 037	19 724	
No. of cases	2207	11 514	1077	483	
Cases/PYs (1/1000)	5.14	3.30	2.46	2.46	
Model 1	1.44 (1.38-1.51)	1.00	0.73 (0.69-0.78)	0.68 (0.62-0.75)	<0.001
Model 2	1.42 (1.36-1.49)	1.00	0.73 (0.69-0.78)	0.68 (0.62-0.75)	<0.001
Model 3	1.43 (1.36-1.50)	1.00	0.73 (0.69-0.78)	0.68 (0.62-0.74)	<0.001
Model 4	1.20 (1.15-1.26)	1.00	0.86 (0.81-0.92)	0.84 (0.77-0.92)	<0.001
**CKD**					
No. of participants	45 706	368 119	45 846	20 749	
No. of cases	821	4786	605	314	
Cases/PYs (1/1000)	1.81	1.31	1.33	1.52	
Model 1	1.20 (1.11-1.29)	1.00	1.05 (0.96-1.14)	1.20 (1.07-1.35)	0.754
Model 2	1.20 (1.11-1.29)	1.00	1.05 (0.96-1.14)	1.20 (1.07-1.35)	0.755
Model 3	1.20 (1.12-1.30)	1.00	1.03 (0.95-1.12)	1.16 (1.04-1.31)	0.360
Model 4	1.15 (1.07-1.24)	1.00	1.07 (0.98-1.17)	1.20 (1.07-1.35)	0.577

HR: hazard ratio; CI: confidence interval; BMF: bowel movement frequency; IHD: ischaemic heart disease; MCEs: major coronary events; COPD: chronic obstructive pulmonary disease; T2DM: type 2 diabetes mellitus; CKD: chronic kidney disease.

Model 1 was adjusted for sex.

Model 2 was additionally adjusted for level of education, occupation, household income, marital status, smoking status, and total physical activity level, and family history (for corresponding disease only; no adjustment for heart failure, COPD, and CKD).

Model 3 was additionally adjusted for alcohol consumption, and intake frequency of fresh vegetables, fresh fruits, and red meat.

Model 4 was additionally adjusted for body mass index, waist circumference, prevalent hypertension and diabetes at baseline (no adjustment for T2DM).

*Assessed by assigning consecutive integers to four BMF categories in a separate model. A Bonferroni-corrected threshold was used, $\alpha=0.00625$.

For peer review only - http://bmjopen.bmj.com/site/about/guidelines.xhtml

Supplementary Table 2 | HRs (95% CIs) for associations between BMF and multiple vascular and nonvascular diseases stratified by sex

Diseases	Subgroups	More than once a day		Once every 1-3 days*		Less than 3 times a week		*P* value for interaction†
		Cases	*HR (95%CI)*	Cases	*HR (95%CI)*	Cases	*HR (95%CI)*	
**Vascular diseases**								
**IHD**								0.099
	Men	2335	1.09 (1.04-1.14)	13 822	1.00	385	1.11 (1.00-1.23)	
	Women	2333	1.16 (1.11-1.21)	20 096	1.00	1376	1.05 (0.99-1.11)	
**MCEs**								0.756
	Men	553	1.00 (0.91-1.09)	3762	1.00	137	1.26 (1.06-1.50)	
	Women	318	1.05 (0.93-1.18)	2963	1.00	239	1.20 (1.05-1.37)	
**Haemorrhagic stroke**								0.322
	Men	645	0.97 (0.89-1.05)	3950	1.00	117	1.19 (0.99-1.43)	
	Women	383	0.96 (0.86-1.07)	3629	1.00	219	1.01 (0.88-1.16)	
**Ischaemic stroke**								0.006
	Men	2234	1.01 (0.97-1.06)	14 229	1.00	460	1.25 (1.13-1.37)	
	Women	1810	1.01 (0.96-1.06)	17 656	1.00	1190	1.04 (0.98-1.11)	
**Heart failure**								0.559
	Men	268	1.26 (1.11-1.44)	1575	1.00	53	0.99 (0.75-1.30)	
	Women	267	1.42 (1.25-1.62)	1891	1.00	150	0.98 (0.83-1.16)	
**Nonvascular diseases**								
**COPD**								0.212
	Men	885	1.25 (1.16-1.35)	4390	1.00	120	1.00 (0.83-1.20)	
	Women	633	1.37 (1.26-1.49)	4703	1.00	323	0.89 (0.80-1.00)	
**T2DM**								0.997
	Men	1035	1.20 (1.12-1.29)	4721	1.00	74	0.89 (0.70-1.12)	
	Women	1172	1.22 (1.15-1.30)	7870	1.00	409	0.84 (0.76-0.93)	
**CKD**								0.249
	Men	423	1.13 (1.01-1.25)	2250	1.00	75	1.38 (1.09-1.74)	
	Women	398	1.16 (1.05-1.29)	3141	1.00	239	1.13 (0.99-1.29)	

For peer review only

For peer review only - http://bmjopen.bmj.com/site/about/guidelines.xhtml

HR: hazard ratio; CI: confidence interval; BMF: bowel movement frequency; IHD: ischaemic heart disease; MCEs: major coronary events; COPD: chronic obstructive pulmonary disease; T2DM: type 2 diabetes mellitus; CKD: chronic kidney disease.

Multivariable models adjusted for same set of covariates as Table 2, except for the stratified variable of interest.

* Combined group including “once a day” and “once every 2-3 days”.

† A Bonferroni-corrected threshold was used, α=0.00625.

For peer review only

Supplementary Table 3 | HRs (95% CIs) for associations between BMF and multiple vascular and nonvascular diseases stratified by baseline age

Diseases	Subgroups	More than once a day		Once every 1-3 days*		Less than 3 times a week		*P* value for interaction†
		Cases	*HR (95%CI)*	Cases	*HR (95%CI)*	Cases	*HR (95%CI)*	
**Vascular diseases**								
**IHD**								0.362
	<60 yr	2531	1.14 (1.09-1.19)	18 064	1.00	810	1.04 (0.97-1.12)	
	⩾60 yr	2137	1.10 (1.06-1.16)	15 854	1.00	951	1.09 (1.02-1.17)	
**MCEs**								0.014
	<60 yr	357	1.08 (0.96-1.21)	2377	1.00	81	1.08 (0.86-1.35)	
	⩾60 yr	514	0.97 (0.88-1.06)	4348	1.00	295	1.25 (1.11-1.41)	
**Haemorrhagic stroke**								0.094
	<60 yr	521	0.96 (0.87-1.05)	3568	1.00	106	0.93 (0.77-1.14)	
	⩾60 yr	507	0.97 (0.88-1.06)	4011	1.00	230	1.15 (1.01-1.32)	
**Ischaemic stroke**								0.902
	<60 yr	2212	1.02 (0.97-1.06)	16 347	1.00	717	1.08 (1.00-1.16)	
	⩾60 yr	1832	1.00 (0.95-1.05)	15 538	1.00	933	1.11 (1.04-1.19)	
**Heart failure**								0.761
	<60 yr	204	1.38 (1.19-1.61)	1269	1.00	52	0.88 (0.66-1.16)	
	⩾60 yr	331	1.31 (1.16-1.47)	2197	1.00	151	1.04 (0.88-1.23)	
**Nonvascular diseases**								
**COPD**								0.309
	<60 yr	706	1.27 (1.17-1.38)	4180	1.00	182	0.96 (0.82-1.11)	
	⩾60 yr	812	1.33 (1.24-1.44)	4913	1.00	261	0.91 (0.81-1.04)	
**T2DM**								0.331
	<60 yr	1549	1.19 (1.13-1.26)	8826	1.00	308	0.83 (0.74-0.94)	
	⩾60 yr	658	1.25 (1.15-1.36)	3765	1.00	175	0.92 (0.79-1.07)	
**CKD**								0.567
	<60 yr	537	1.20 (1.10-1.32)	3446	1.00	189	1.19 (1.02-1.38)	

For peer review only - http://bmjopen.bmj.com/site/about/guidelines.xhtml

For peer review only

Diseases	Subgroups	More than once a day		Once every 1-3 days*		Less than 3 times a week		*P* value for interaction†
		Cases	*HR (95%CI)*	Cases	*HR (95%CI)*	Cases	*HR (95%CI)*	
	⩾60 yr	284	1.06 (0.94-1.20)	1945	1.00	125	1.17 (0.98-1.41)	

HR: hazard ratio; CI: confidence interval; BMF: bowel movement frequency; IHD: ischaemic heart disease; MCEs: major coronary events; COPD: chronic obstructive pulmonary disease; T2DM: type 2 diabetes mellitus; CKD: chronic kidney disease.

Multivariable models adjusted for same set of covariates as Table 2, except for the stratified variable of interest.

* Combined group including “once a day” and “once every 2-3 days”.

† A Bonferroni-corrected threshold was used, α=0.00625.

For peer review only

Supplementary Table 4 | HRs (95% CIs) for associations between BMF and multiple vascular and nonvascular diseases stratified by baseline hypertension

Diseases	Subgroups	More than once a day		Once every 1-3 days*		Less than 3 times a week		*P* value for interaction†
		Cases	*HR (95%CI)*	Cases	*HR (95%CI)*	Cases	*HR (95%CI)*	
**Vascular diseases**								
**IHD**								0.192
	No	2019	1.17 (1.12-1.23)	16 300	1.00	995	1.07 (1.00-1.14)	
	Yes	2649	1.09 (1.04-1.13)	17 618	1.00	766	1.06 (0.99-1.14)	
**MCEs**								0.102
	No	328	1.12 (1.00-1.26)	2498	1.00	157	1.22 (1.04-1.44)	
	Yes	543	0.96 (0.88-1.05)	4227	1.00	219	1.20 (1.05-1.38)	
**Haemorrhagic stroke**								0.059
	No	266	1.00 (0.87-1.13)	2108	1.00	128	1.17 (0.98-1.41)	
	Yes	762	0.95 (0.88-1.03)	5471	1.00	208	1.01 (0.88-1.17)	
**Ischaemic stroke**								0.134
	No	1441	1.03 (0.97-1.09)	13 429	1.00	845	1.10 (1.02-1.18)	
	Yes	2603	0.99 (0.95-1.03)	18 456	1.00	805	1.09 (1.02-1.17)	
**Heart failure**								0.432
	No	230	1.47 (1.27-1.69)	1570	1.00	102	0.95 (0.78-1.16)	
	Yes	305	1.25 (1.10-1.41)	1896	1.00	101	1.05 (0.85-1.28)	
**Nonvascular diseases**								
**COPD**								0.654
	No	779	1.29 (1.20-1.40)	5191	1.00	278	0.92 (0.81-1.04)	
	Yes	739	1.30 (1.20-1.41)	3902	1.00	165	0.96 (0.82-1.13)	
**T2DM**								0.011
	No	925	1.26 (1.18-1.36)	6063	1.00	264	0.82 (0.72-0.93)	
	Yes	1282	1.16 (1.10-1.24)	6528	1.00	219	0.96 (0.84-1.10)	
**CKD**								0.255
	No	355	1.20 (1.08-1.35)	2605	1.00	170	1.12 (0.96-1.32)	

For peer review only - http://bmjopen.bmj.com/site/about/guidelines.xhtml

Diseases	Subgroups	More than once a day		Once every 1-3 days*		Less than 3 times a week		*P* value for interaction†
		Cases	*HR (95%CI)*	Cases	*HR (95%CI)*	Cases	*HR (95%CI)*	
	Yes	466	1.10 (0.99-1.21)	2786	1.00	144	1.29 (1.09-1.53)	

HR: hazard ratio; CI: confidence interval; BMF: bowel movement frequency; IHD: ischaemic heart disease; MCEs: major coronary events; COPD: chronic obstructive pulmonary disease; T2DM: type 2 diabetes mellitus; CKD: chronic kidney disease..

Multivariable models adjusted for same set of covariates as Table 2, except for the stratified variable of interest.

* Combined group including “once a day” and “once every 2-3 days”.

† A Bonferroni-corrected threshold was used, α=0.00625.

For peer review only

Supplementary Note

1. CKB collaborative group

International Steering Committee: Junshi Chen, Zhengming Chen (PI), Robert Clarke, Rory Collins, Yu Guo, Liming Li (PI), Jun Lv, Richard Peto, Robin Walters. **International Co-ordinating Centre, Oxford:** Daniel Avery, Ruth Boxall, Derrick Bennett, Yumei Chang, Yiping Chen, Zhengming Chen, Robert Clarke, Huaidong Du, Simon Gilbert, Alex Hacker, Mike Hill, Michael Holmes, Andri Iona, Christiana Kartsonaki, Rene Kerosi, Ling Kong, Om Kurmi, Garry Lancaster, Sarah Lewington, Kuang Lin, John McDonnell, Iona Millwood, Qunhua Nie, Jayakrishnan Radhakrishnan, Paul Ryder, Sam Sansome, Dan Schmidt, Paul Sherliker, Rajani Sohoni, Becky Stevens, Iain Turnbull, Robin Walters, Jenny Wang, Lin Wang, Neil Wright, Ling Yang, Xiaoming Yang. **National Co-ordinating Centre, Beijing:** Zheng Bian, Yu Guo, Xiao Han, Can Hou, Jun Lv, Pei Pei, Chao Liu, Yunlong Tan, Canqing Yu. **10 Regional Co-ordinating Centres: Qingdao CDC:** Zengchang Pang, Ruqin Gao, Shanpeng Li, Shaojie Wang, Yongmei Liu, Ranran Du, Yajing Zang, Liang Cheng, Xiaocao Tian, Hua Zhang, Yaoming Zhai, Feng Ning, Xiaohui Sun, Feifei Li. **Licang CDC:** Silu Lv, Junzheng Wang, Wei Hou. **Heilongjiang Provincial CDC:** Mingyuan Zeng, Ge Jiang, Xue Zhou. **Nangang CDC:** Liqiu Yang, Hui He, Bo Yu, Yanjie Li, Qinai Xu,Quan Kang, Ziyan Guo. **Hainan Provincial CDC:** Dan Wang, Ximin Hu, Jinyan Chen, Yan Fu, Zhenwang Fu, Xiaohuan Wang. **Meilan CDC:** Min Weng, Zhendong Guo, Shukuan Wu,Yilei Li, Huimei Li, Zhifang Fu. **Jiangsu Provincial CDC:** Ming Wu, Yonglin Zhou, Jinyi Zhou, Ran Tao, Jie Yang, Jian Su. **Suzhou CDC:** Fang liu, Jun Zhang, Yihe Hu, Yan Lu, , Liangcai Ma, Aiyu Tang, Shuo Zhang, Jianrong Jin, Jingchao Liu. **Guangxi Provincial CDC:** Zhenzhu Tang, Naying Chen, Ying Huang. **Liuzhou CDC:** Mingqiang Li, Jinhuai Meng, Rong Pan, Qilian Jiang, Jian Lan,Yun Liu, Liuping Wei, Liyuan Zhou, Ningyu Chen Ping Wang, Fanwen Meng, Yulu Qin,, Sisi Wang. **Sichuan Provincial CDC:** Xianping Wu,

For peer review only

Ningmei Zhang, Xiaofang Chen,Weiwei Zhou. **Pengzhou CDC:** Guojin Luo, Jianguo Li, Xiaofang Chen, Xunfu Zhong, Jiaqiu Liu, Qiang Sun. **Gansu Provincial CDC:** Pengfei Ge, Xiaolan Ren, Caixia Dong. **Maiji CDC:** Hui Zhang, Enke Mao, Xiaoping Wang, Tao Wang, Xi zhang. **Henan Provincial CDC:** Ding Zhang, Gang Zhou, Shixian Feng, Liang Chang, Lei Fan. **Huixian CDC:** Yulian Gao, Tianyou He, Huarong Sun, Pan He, Chen Hu, Xukui Zhang, Huifang Wu, Pan He. **Zhejiang Provincial CDC:** Min Yu, Ruying Hu, Hao Wang. Tongxiang CDC: Yijian Qian, Chunmei Wang, Kaixu Xie, Lingli Chen, Yidan Zhang, Dongxia Pan, Qijun Gu. **Hunan Provincial CDC:** Yuelong Huang, Biyun Chen, Li Yin, Huilin Liu, Zhongxi Fu, Qiaohua Xu. **Liuyang CDC:** Xin Xu, Hao Zhang, Huajun Long, Xianzhi Li, Libo Zhang, Zhe Qiu.

For peer review only

For peer review only - http://bmjopen.bmj.com/site/about/guidelines.xhtml

2. Food frequency questionnaire

Section 5: Diet

5.1 During the past 12 months, about how often did you eat the following foods?

	Daily	4-6 days per week	1-3 days per week	Monthly	Never /rarely
Rice					
Wheat					
Other staple food (corn, millet etc.)					
Meat					
Poultry					
Fish/sea food					
Fresh eggs					
Fresh vegetables					
Soybean products					
Preserved vegetables					
Fresh fruit					
Dairy products (milk, yogurt)					

5.2 During the past 12 months, have you taken the following supplements regularly?

Yes	No	
		Fish oil/cod liver oil
		Vitamins
		Calcium/iron/zinc
		Ginseng (at least 5 or more times during a year)
		Other herbal products

(5.3 to 5.6 are omitted)

5.7 During the past month, about how often did you eat hot spicy food?

- ☐ Never or almost never → Go to section 6
- ☐ Only occasionally → Go to section 6
- ☐ 1-2 days/week
- ☐ 3-5 days/week
- ☐ Daily or almost every day

The complete questionnaire is available below:
https://www.ckbiobank.org/site/Study+Resources/Baseline+Data

The categories of covariates in Cox models: intake frequency of rice, wheat, other staple food (daily, 4-6 days/week, 1-3 days/week, monthly, or rarely or never); spicy food (6-7, 3-5, 1-2 days/week, occasionally, never); consumption of nutritional supplements (fish oil/cod liver oil, vitamins, calcium/iron/zinc, ginseng and other herbal products; yes or no);

For peer review only - http://bmjopen.bmj.com/site/about/guidelines.xhtml

STROBE Statement—Checklist of items that should be included in reports of ***cohort studies***

	Item No	Recommendation	Page No
**Title and abstract**	1	(*a*) Indicate the study's design with a commonly used term in the title or the abstract	1
		(*b*) Provide in the abstract an informative and balanced summary of what was done and what was found	2-3
**Introduction**			
Background/rationale	2	Explain the scientific background and rationale for the investigation being reported	4
Objectives	3	State specific objectives, including any prespecified hypotheses	5
**Methods**			
Study design	4	Present key elements of study design early in the paper	5
Setting	5	Describe the setting, locations, and relevant dates, including periods of recruitment, exposure, follow-up, and data collection	5-8
Participants	6	(*a*) Give the eligibility criteria, and the sources and methods of selection of participants. Describe methods of follow-up	5-6, 7-8
		(*b*) For matched studies, give matching criteria and number of exposed and unexposed	N/A
Variables	7	Clearly define all outcomes, exposures, predictors, potential confounders, and effect modifiers. Give diagnostic criteria, if applicable	6-8
Data sources/ measurement	8*	For each variable of interest, give sources of data and details of methods of assessment (measurement). Describe comparability of assessment methods if there is more than one group	6-7
Bias	9	Describe any efforts to address potential sources of bias	5-6, 8-9
Study size	10	Explain how the study size was arrived at	5
Quantitative variables	11	Explain how quantitative variables were handled in the analyses. If applicable, describe which groupings were chosen and why	7, 10
Statistical methods	12	(*a*) Describe all statistical methods, including those used to control for confounding	8, 9
		(*b*) Describe any methods used to examine subgroups and interactions	10
		(*c*) Explain how missing data were addressed	5
		(*d*) If applicable, explain how loss to follow-up was addressed	7
		(*e*) Describe any sensitivity analyses	9
**Results**			
Participants	13*	(a) Report numbers of individuals at each stage of study—eg numbers potentially eligible, examined for eligibility, confirmed eligible, included in the study, completing follow-up, and analysed	5-6
		(b) Give reasons for non-participation at each stage	N/A
		(c) Consider use of a flow diagram	—
Descriptive data	14*	(a) Give characteristics of study participants (eg demographic, clinical, social) and information on exposures and potential confounders	11, 23
		(b) Indicate number of participants with missing data for each variable of interest	N/A
		(c) Summarise follow-up time (eg, average and total amount)	11
Outcome data	15*	Report numbers of outcome events or summary measures over time	11

For peer review only - http://bmjopen.bmj.com/site/about/guidelines.xhtml

Main results	16	(*a*) Give unadjusted estimates and, if applicable, confounder-adjusted estimates and their precision (eg, 95% confidence interval). Make clear which confounders were adjusted for and why they were included	11
		(*b*) Report category boundaries when continuous variables were categorized	10
		(*c*) If relevant, consider translating estimates of relative risk into absolute risk for a meaningful time period	—
Other analyses	17	Report other analyses done—eg analyses of subgroups and interactions, and sensitivity analyses	12
**Discussion**			
Key results	18	Summarise key results with reference to study objectives	13
Limitations	19	Discuss limitations of the study, taking into account sources of potential bias or imprecision. Discuss both direction and magnitude of any potential bias	17
Interpretation	20	Give a cautious overall interpretation of results considering objectives, limitations, multiplicity of analyses, results from similar studies, and other relevant evidence	13-16
Generalisability	21	Discuss the generalisability (external validity) of the study results	16-17
**Other information**			
Funding	22	Give the source of funding and the role of the funders for the present study and, if applicable, for the original study on which the present article is based	18

*Give information separately for exposed and unexposed groups.

Note: An Explanation and Elaboration article discusses each checklist item and gives methodological background and published examples of transparent reporting. The STROBE checklist is best used in conjunction with this article (freely available on the Web sites of PLoS Medicine at http://www.plosmedicine.org/, Annals of Internal Medicine at http://www.annals.org/, and Epidemiology at http://www.epidem.com/). Information on the STROBE Initiative is available at http://www.strobe-statement.org.

For peer review only

For peer review only - http://bmjopen.bmj.com/site/about/guidelines.xhtml
